# Leveraging Multiproton-Coupled Electron Transfer to Improve Ir(III) Photocatalyst Efficiency

**DOI:** 10.1021/acs.jpcc.5c08623

**Published:** 2026-02-05

**Authors:** Eris Villalona, Rodrigo E. Domínguez, Edwin J. Gonzalez Lopez, Walter D. Guerra, Daniel A. Heredia, Anton Y. Khmelnitskiy, Daniel G. Oblinsky, Yohana Palacios, Thomas A. Moore, Gregory D. Scholes, Robert R. Knowles, Ana L. Moore

**Affiliations:** † Department of Chemistry, 6740 Princeton University, Princeton, New Jersey 08544, United States; ‡ School of Molecular Sciences, 7864 Arizona State University, Tempe, Arizona 85287, United States; § Instituto Para el Desarrollo Agroindustrial y de la Salud (IDAS-CONICET). Departamento de Química, Facultad de Ciencias Exactas, Físico-Químicas y Naturales, Universidad Nacional de Río Cuarto, Ruta Nacional 36 Km 601, X5804BYA Río Cuarto, Córdoba, Argentina

## Abstract

In photoredox reactions, charge recombination (CR) limits quantum yields, hindering the efficient conversion of light energy into catalytic activity. To address this, we drew inspiration from redox relays in photosystem II (PSII) and developed a new series of iridium­(III) complexes featuring covalently attached benzimidazole-phenol-pyridine (BIP-Py) groups to facilitate intramolecular multiproton-coupled electron transfer (MPCET). Herein, we evaluate the effects of MPCET through an extended and well-defined hydrogen-bond network to improve photocatalytic activity and mitigate rapid charge recombination. Infrared spectroelectrochemistry reveals pyridine protonation upon phenol oxidation, while visible spectroelectrochemistry and transient absorption spectroscopy confirm the electro- and photochemical formation of charge-separated states (CSS) involving oxidized BIP, resulting from intramolecular proton-coupled electron transfer (PCET). The application of the BIP-Py platform in a photocatalytic *N*-hydroxyphthalimide ester reduction reaction resulted in a ∼106-fold reduction in CR rate and a quantum yield enhancement of up to 157%. Our findings suggest that incorporating MPCET-based redox relays into photocatalyst frameworks is an effective strategy to enhance the efficiency of photocatalytic systems.

## Graphical abstract

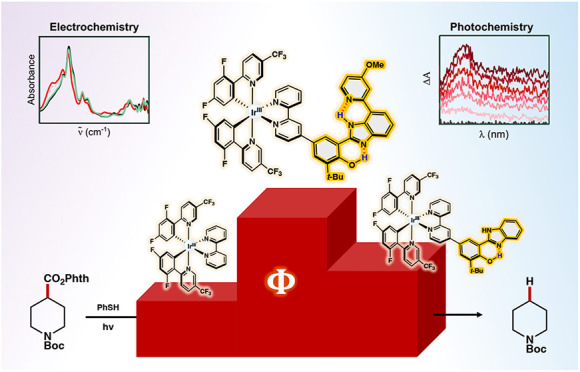

## 1 Introduction

Photoredox catalysis utilizes light energy to drive chemical transformations, offering an alternative to traditional thermal activation methods.^1,2^ By leveraging photoinduced electron transfer (ET) between a photocatalyst and a substrate, photoredox catalysis generates reactive radical intermediates under mild conditions, facilitating a broad range of synthetic transformations that are challenging or inaccessible using conventional methodologies.^3,4^ These advantages make photoredox catalysis an attractive platform for chemical synthesis, with applications spanning from small-molecule activation to complex bond-forming reactions. However, the broad application of photoredox catalysis is fundamentally constrained by charge recombination (CR) processes, which limit quantum efficiency.^5^ After excitation, the photocatalyst generates radical or radical ion species, which can undergo undesired recombination with the oxidized or reduced form of the catalyst before productive reactivity occurs.^5–9^ As shown in Figure 1A, this charge recombination competes with the desired forward reaction, resulting in energy losses and reduced catalytic efficiency. Therefore, strategies to suppress charge recombination and extend the lifetimes of charge-separated states offer an opportunity to improve the performance of photoredox catalysis.

**Figure 1:**
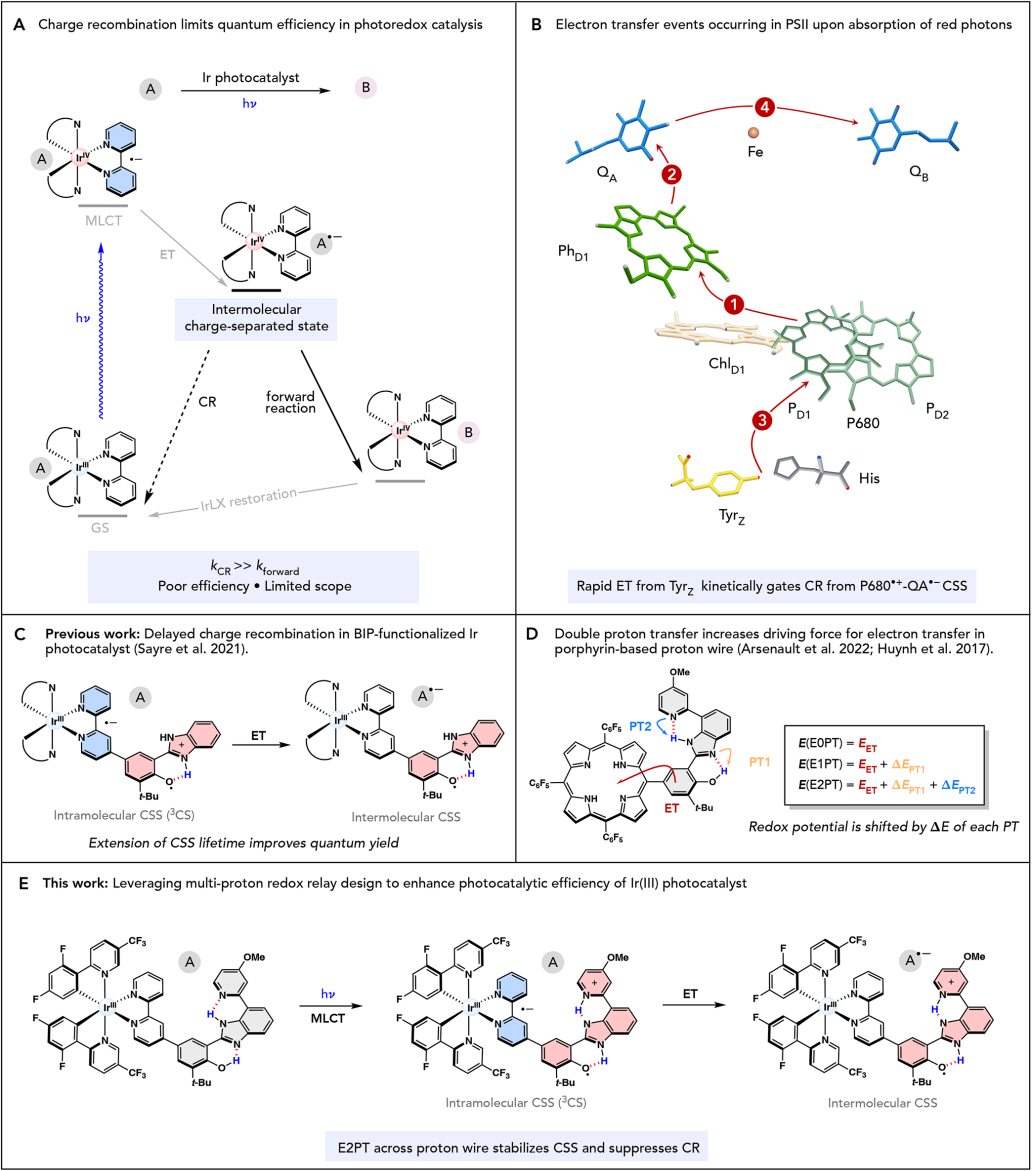
(A) Charge recombination from the charge-separated state leads to loss of chemical potential, limiting quantum efficiency in photoredox catalysis. (B) Partial, simplified scheme of PSII showing order (numbered circles) of electron transfer events (red arrows) upon excitation. Adapted with permission from ref 10. Copyright 2012 Elsevier. (C) Appending a benzimidazole–phenol (BIP) PCET ligand to an Ir­(III) photocatalyst produces a long-lived intermolecular charge-separated state, slowing charge recombination. (D) Biomimetic construct bearing a BIP-Py platform undergoes photoinduced PCET and long-range proton transport across an extended proton relay scaffold. (E) BIP-derived proton wire enables a concerted one-electron, two-proton transfer (E2PT) that further stabilizes the charge-separated state, suppresses recombination, and delivers enhanced photocatalytic efficiency.

Efforts to address these limitations have drawn inspiration from oxygenic photosynthesis. In all oxygenic photosynthetic organisms, water oxidation is carried out by a light-driven enzyme complex, photosystem II (PSII). Photochemical processes in PSII begin with excitation of a chlorophyll complex, P680, creating a singlet-excited state (P680*). P680* then engages in rapid electron transfer, reducing neighboring chlorophyll (Chl) molecules, ultimately generating a charge-separated state complex, [P680^•+^-QA^•–^], with a quinone cofactor from which back electron transfer could kinetically compete with forward relay of the reducing equivalent through the electron transport chain.^10^ To mitigate competitive charge recombination, PSII employs a redox relay system involving a hydrogen-bonded tyrosine-histidine (Tyr_*z*_–H190) amino acid complex that rapidly reduces P680^•+^ via a proton-coupled electron transfer (PCET) event (Figure 1B).^10–13^ In this way, the Tyr_Z_–H190 redox relay serves as an intermediate electron–proton transfer bridge, coupling the fast photochemical charge separation at P680 with the slower downstream electron transfer steps, thereby maximizing the photochemical quantum yield.^14,15^

Inspired by this mechanism, artificial constructs such as the benzimidazole-phenol (BIP) platform have been developed to mimic the structural and redox properties of the Tyr_Z_–H190 pair,^14–19^ serving as a versatile system to probe PCET thermodynamics and enhance quantum efficiency in photoredox catalysis.^20–22^ Beyond redox relays, molecular dyads, covalently linked photosensitizer–acceptor conjugates that funnel the photosensitizer-localized excitation into a long-lived triplet on the acceptor, and Coulombic dyads, oppositely charged ion-paired chromophores that enable static energy transfer/ET can prolong excited-state lifetimes and favor productive reactivity.^23–27^ Separately, recent work shows that cage-escape efficiency often governs photoredox rates and quantum yields and can be tuned via solvent polarity/viscosity, ion-pairing, and catalyst-dependent in-cage back-electron transfer.^28–32^ In this study, we present an alternative strategy focusing on covalently integrating a PCET-based relay within the photocatalyst.

Previously, we demonstrated that functionalizing an Ir­(III) photocatalyst with a BIP motif enabled an intramolecular PCET mechanism that improved charge separation and quantum yield in a model photocatalytic reaction (Figure 1C).^22^ Notably, these improvements were observed despite the shorter excited-state lifetime of the Ir-BIP catalyst (**2**) compared to [Ir­(dF­(CF_3_)­ppy)_2_(bpy)]­[PF_6_] (**1**). This apparent contradiction can be reconciled by considering the formation of a longer-lived intermolecular charge-separated complex, allowing the singly reduced substrate to proceed toward productive chemistry. Here, the efficiency gains arise not from extended excited-state lifetime but from the effective decoupling of unproductive charge recombination and chemical transformation via the stabilization of charge-separated species.

These findings inspired the development of new ligand architectures designed to suppress charge recombination further and enhance photocatalytic efficiency. To improve the catalytic performance of the Ir-BIP platform, we incorporated design principles derived from multiproton-coupled electron transfer (MPCET) systems.^19,33–35^ Specifically, we introduced a Brønsted base as a terminal proton acceptor (TPA) at the C7-position of the benzimidazole moiety in our BIP-functionalized Ir­(III) photocatalyst, thereby establishing an extended hydrogen-bonded network spanning from the phenolic proton to the distal TPA group. In studies by Huynh and co-workers, BIP constructs bearing a TPA were shown to undergo concerted two-proton-coupled electron transfer (E2PT) upon phenol oxidation, facilitating rapid Grotthuss-type proton translocation across a proton wire.^19^ Notably, these studies reveal that inclusion of a distal TPA, such as a tertiary amine or imine group, lowers the redox potential of the phenol, depending on the identity of the TPA and the number of benzimidazole bridging units. However, this could be prevented by using lower p*K*_a_ TPAs and by substituting the benzimidazole bridging units with electron-withdrawing groups.^36,37^ This redox modulation reduces the activation barrier for PCET, promoting faster reaction kinetics. Moreover, studies by Arsenault and co-workers (Figure 1D) have shown that E2PT architectures significantly impact the thermodynamics of PCET. Using a porphyrin covalently functionalized with a benzimidazole-phenol-pyridine (BIP-Py) proton wire, they demonstrated that E2PT product formation exhibits a more favorable driving force compared to that of an E1PT (one-proton-coupled electron transfer) analog as supported by both electrochemical measurements and quantum chemical calculations.^35^

Building on these experimental insights, herein we present the design, synthesis, and characterization of a new family of Ir­(III) complexes aimed at promoting two-proton redox relay pathways. We hypothesized that appending a BIP-Py group to an Ir­(III) photosensitizer would enhance photocatalytic performance by enabling an E2PT mechanism. Specifically, we anticipate that functionalization with a distal proton acceptor would lower the oxidation potential of the phenol moiety, accelerating PCET kinetics aided by proton translocation through an extended hydrogen-bond network. In addition, spatial separation of the oxidized and protonated sites is expected to further stabilize the charge-separated state relative to the parent Ir-BIP catalyst, thereby raising the barrier for charge recombination and promoting catalytic cycling (Figure 1E). To this end, we synthesized three novel heteroleptic Ir­(III) BIP-Py complexes, where the BIP-bpy ligand is substituted at the C7-position of the BIP backbone with isomeric methoxypyridine groups to afford the following catalysts: (**3**) *o*-OMe-2-Py-BIP, (**4**) *p*-OMe-2-Py-BIP, and (**5**) *o*-OMe-4-Py-BIP, as shown in Figure 2. By leveraging this rational ligand design, we aim to establish a framework for extending the lifetime of charge-separated states through multiproton redox relays, thereby improving the efficiency of photocatalytic transformations.

**Figure 2:**
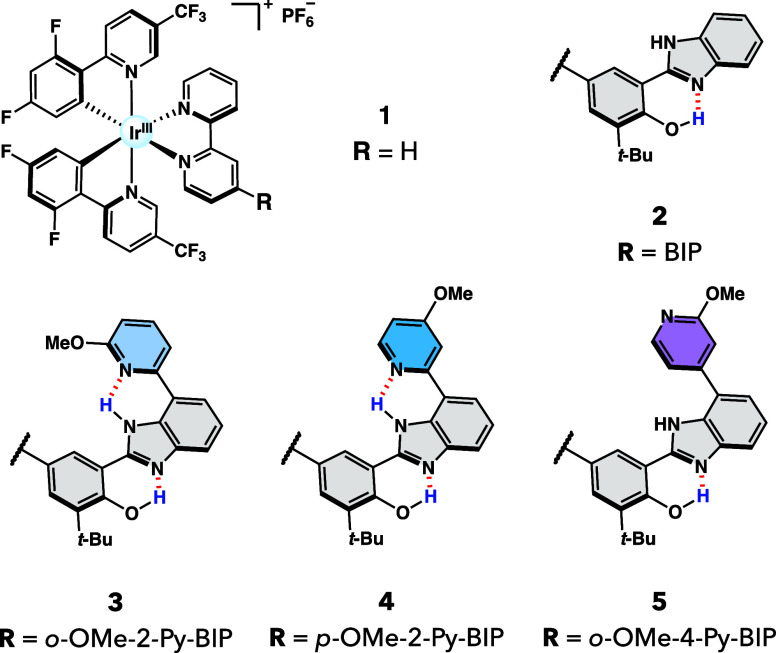
Molecular structures of Ir­(III) photocatalysts in this study.

## 2 Methods

### 2.1 Synthesis of Photocatalysts

Iridium­(III) photocatalysts **1**–**5** were synthesized according to previously reported procedures.^22,38,39^ The detailed synthetic routes, conditions, and characterization of photocatalysts Ir-BIP-Py **3**–**5** are outlined in Scheme S1 (see the Supporting Information, Section S1.2).

### 2.2 Steady-State Absorption and Emission Spectroscopy

UV–visible absorption and steady-state emission spectra for photocatalysts **1**–**5** were collected at room temperature in dry tetrahydrofuran (THF) and acetonitrile (MeCN) (Figures S31–S32). Absorption spectra were recorded in 1 cm quartz cuvettes, with solutions diluted as needed to maintain comparable absorbance in the visible region across the series. Emission spectra were obtained using dilute solutions to minimize inner-filter effects, with excitation wavelengths chosen to overlap the visible absorption bands of each photocatalyst.

### 2.3 Electrochemical Measurements

Electrochemical characterization was carried out by cyclic voltammetry using a three-electrode setup in dry MeCN containing 0.1 M tetrabutylammonium hexafluorophosphate (nBu_4_NPF_6_) as the supporting electrolyte. Measurements were conducted under inert atmosphere using a glassy carbon working electrode, a platinum counter electrode, and a silver pseudoreference electrode. Potentials were referenced to the ferrocenium/ferrocene (Fc^+^/Fc) couple as an internal standard, and voltammograms were collected at a scan rate of 1 V s^–1^.

### 2.4 Spectroelectrochemistry

Infrared spectroelectrochemistry (IRSEC) experiments were performed in MeCN containing 0.1 M nBu_4_NPF_6_. Solutions were prepared at millimolar concentrations and analyzed at a spectral resolution of 2 cm^–1^. Visible-range spectroelectrochemistry was performed in an analogous electrolyte solution under inert conditions to track electrochemically generated states by UV–visible absorption.

### 2.5 Transient Absorption Spectroscopy

The photophysical properties of photocatalysts **3–5** were investigated by visible ultrafast and nanosecond transient absorption spectroscopy (TAS) in anhydrous, degassed MeCN and THF. Samples were prepared under inert atmosphere and transferred to sealed quartz cuvettes. Photoexcitation was performed at 440 nm, and broadband probe detection was used to monitor transient absorption features and decay kinetics across the visible region. Data analysis was performed using Surface Xplorer and Glotaran software (see Section S1.9).^40^

### 2.6 Photon Flux Determination and Quantum Yield Measurements

Photon flux for 440 nm irradiation was determined using potassium ferrioxalate actinometry under conditions matched to preparative photoredox experiments. Aqueous potassium ferrioxalate was irradiated at a fixed distance from a 440 nm Kessil lamp equipped with a 440 nm bandpass interference filter (FWHM = 10 nm). Aliquots were developed using a 1,10-phenanthroline reagent system to generate the Fe­(phen)_3_^2+^ chromophore, and absorbance at 510 nm was used for quantification.

Initial-rate experiments used to determine internal quantum yields were conducted in a sealed quartz cuvette equipped with a micro stir bar. Reactions were assembled under inert atmosphere using an *N*-hydroxyphthalimide ester substrate, thiophenol as the hydrogen atom donor, photocatalyst (adjusted to an absorbance of OD_440 nm_ = 1.1), and THF as solvent (0.1 M). The cuvette was positioned 4 cm from the 40 W 440 nm Kessil lamp and maintained at 25 °C using a water bath and active air cooling. Reaction aliquots were removed at defined time points and analyzed by GC using an internal standard to obtain initial rates for quantum yield calculations.

## 3 Results and Discussion

### 3.1 Design

In designing new BIP-based photocatalysts, we selected pyridyl groups as terminal proton acceptors (TPAs) based on several key advantages. Pyridyl groups are robust under a wide range of conditions, including hydrolysis, offering a stability advantage over previously explored BIP-imine systems.^19,34,36,38^ Furthermore, their p*K*_a_ values closely match those of the benzimidazole bridge, enabling favorable proton affinity matching and promoting efficient proton transfer across the hydrogen-bond network.^41,42^ Additionally, the distinct vibrational features of neutral and protonated pyridine forms enable the direct monitoring of proton transfer events using infrared spectroelectrochemistry (IRSEC).^43^

To systematically evaluate the impact of TPA identity and proton relay architecture on catalytic performance, we designed three Ir­(III) BIP-Py complexes featuring distinct hydrogen-bond networks. Compounds **3** and **4** feature an extended hydrogen-bond network spanning from the phenol to the pyridine TPA and are designed to undergo a one-electron, two-proton transfer process upon photoexcitation. Compound **3** has the methoxy group located at the ortho-position relative to the nitrogen in the pyridine. This substitution is known to decrease the basicity of the pyridine nitrogen relative to the para-methoxy location in compound **4**.^44^ As a result, the terminal proton acceptor in **4** is expected to exhibit higher proton affinity, as evidenced by the downfield shifts of both the phenolic O–H and the benzimidazole N–H ^1^H NMR resonances in **4** versus **3** (see Section S1.3). This stronger hydrogen-bond network in **4** should facilitate a more favorable E2PT process.^33,38,39,45^ By contrast, in compound **5**, the hydrogen-bond network has been modified to disrupt its continuity, preventing the E2PT event. Previous electrochemical studies on this interrupted topology revealed a chemically reversible oxidation process at high scan rates, with an E_1/2_ characteristic of a one-proton-coupled electron transfer (E1PT) mechanism. At slower scan rates, the process becomes chemically irreversible, reflecting a sequential two-step pathway in which intramolecular E1PT is followed by a slower, likely intermolecular, proton transfer.^39^ These three catalyst architectures establish a platform for directly probing how the hydrogen-bond network and pyridine basicity govern MPCET efficiency and influence photocatalytic performance.

### 3.2 Steady-State Absorption and Emission Spectroscopy

The spectra of **1** and **2** are consistent with literature reports.^22,45^ Compounds **1**–**5** exhibit typical Ir­(III) absorption features, including π–π* transitions in the UV region and a mixed ligand-centered (LC) and metal-to-ligand charge transfer (MLCT) transition at lower energy (assigned to ppy π → π* and Ir­(d) → ppy­(π*) character, respectively).^22^ The main difference in the absorption spectra of the novel photocatalysts **3**–**5** compared to **2** lies in the presence of a distinctive shoulder at approximately 360 nm. This shoulder exhibits a slight blue shift in compounds **4** and **5** at around 350 nm. This spectral feature can be attributed to the addition of the methoxypyridine group, as suggested by the absorption spectra of the pyridinyl-benzothiadiazoles intermediates (**15a** and **15c**, Figure S31B), and is assigned to a π-π* electronic transition within the conjugated system of this group.^46^

Previously, it was shown that compound **2** exhibits a significantly quenched emissive excited state compared to compound **1**. This emission is recovered upon the addition of 10 mM phenylphosphoric acid, indicating that protonation of the imidazole proton acceptor suppresses PCET. The recovery of emission upon acid addition suggests that PCET quenches the emission of compound **2**.^22^ In the case of compounds **3**–**5**, no detectable emission was observed under neutral conditions, indicating that the PCET is likely operative and responsible for quenching the excited state. This interpretation is further supported by the partial recovery of emission upon the addition of 10 mM phenylphosphoric acid (Figure S32B), suggesting protonation of the TPA imidazole and/or pyridine in acidic media suppresses the PCET process.

### 3.3 Electrochemical Characterization

Cyclic voltammetry (CV) experiments were conducted to investigate the influence of structural modifications on the electrochemical properties of photocatalysts **3**–**5**. The resulting voltammograms and the experimental midpoint potentials (*E*_1/2_, calculated as the average of the anodic and cathodic peaks) are presented in Figure 3 and summarized in Table 1. All compounds show a reversible one-electron reduction wave, which was attributed to the reduction of the bipyridyl π* orbital^47,48^ (see the Supporting Information, Section S1.6). The reduction potentials of reference compounds **1** and **2** were consistent with previously reported values.^22^ Compared to **1**, compound **3** displays a similar reduction potential (*E*_1/2_ = −1.65 V vs Fc+/Fc), while compound **4** shows a slightly more negative reduction potential (*E*_1/2_ = −1.67 V vs Fc+/Fc). Compound **5** exhibits a reduction potential that is 40 mV more positive (*E*_1/2_ = −1.60 V vs Fc+/Fc) than that of compound **1**. These differences indicate that the covalent attachment of the BIP or BIP-Py moiety to the periphery of photocatalysts **2**–**5** does not produce any consistent shift in the ligand-based bpy^0/–^ redox couple. By contrast, oxidation potentials track accordingly with respect to changes in the proton-relay architecture and TPA identity. As previously reported for compound **2**, with an experimental oxidation midpoint potential at +0.72 V vs Fc+/Fc, compounds **3** and **4** exhibit analogous quasi-reversible one-electron oxidation waves corresponding to the phenoxyl radical/phenol (PhO^•^/PhOH) redox couple at +0.69 and +0.66 V vs Fc+/Fc, respectively (Figure 3). These cathodically shifted potentials relative to compound **2** are consistent with increased thermodynamic favorability resulting from coupling the one-electron oxidation to a second proton translocation, characteristic of a concerted E2PT process. In contrast, compound **5**, with its interrupted proton relay, exhibits an oxidation wave at +0.72 V vs Fc+/Fc, nearly identical to that of compound **2**, suggesting an E1PT-type process is operative. Further characterization of the oxidation process, using IRSEC (*vide infra*), was employed to confirm whether E2PT products are generated for compounds **3** and **4** and to clarify the proposed PCET process for compound **5**.

**Table 1:** Electrochemical Potentials of Photocatalysts **1–5** and Uncoordinated **BIP-bpy L**

photocatalyst	*E*_1/2_ [BIP^•+^/BIP]^a^	*E*_1/2_ [bpy^0^/bpy^–^]^a^
**1**	n/a	–1.64
**2**	0.72	–1.62
**3**	0.69	–1.65
**4**	0.66	–1.67
**5**	0.72	–1.60
**BIP-bpy L**	0.71	–1.99

a Reported in V vs Fc^+^/Fc in MeCN.

**Figure 3:**
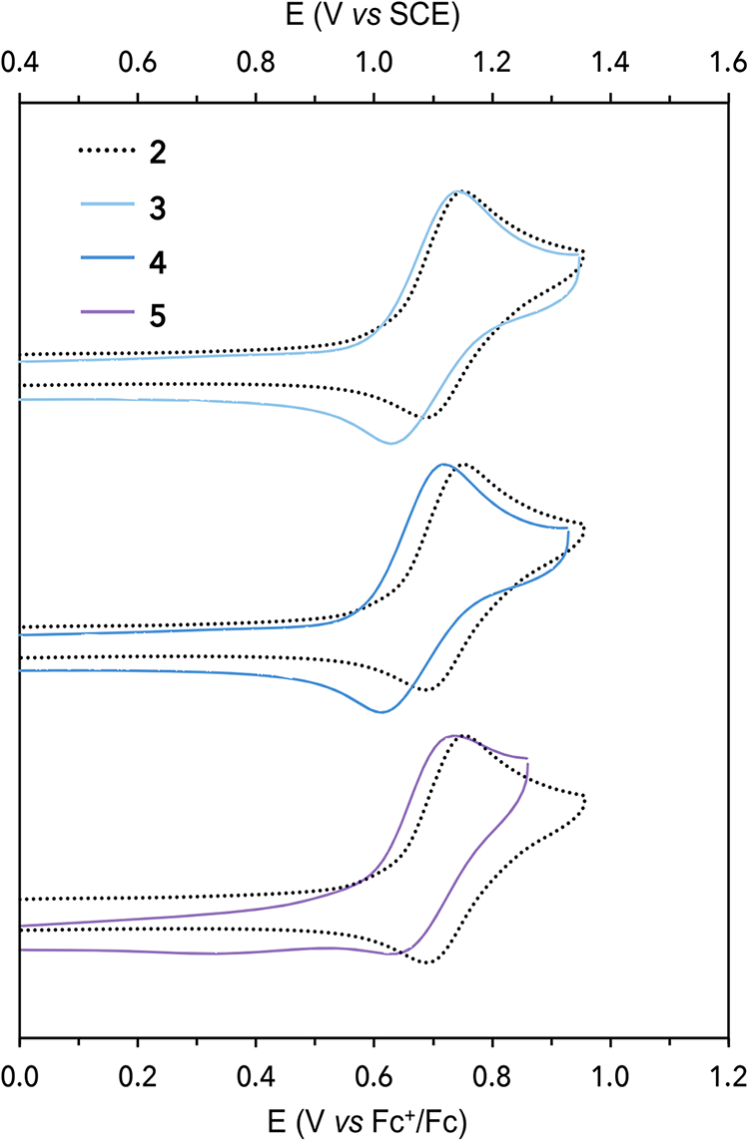
Cyclic voltammograms of **2–5**. Electrochemical measurements were performed in a 0.1 M nBu_4_NPF_6_ as supporting electrolyte in degassed MeCN containing 1 mM of the photocatalyst. A glassy carbon working electrode, a silver wire pseudoreference electrode (with ferrocene as internal reference), and a platinum wire counter electrode were used with a scan rate of 1 V s^–1^.

### 3.4 Infrared Spectroelectrochemistry (IRSEC)

IRSEC is a powerful technique for monitoring vibrational mode changes in transition metal photocatalysts during oxidation and reduction processes. Here, it was employed to provide spectroscopic evidence for PCET processes. Figure 4 displays the IRSEC spectra of compounds **3**, **4**, and **5** in acetonitrile under oxidative conditions within the 1650–1450 cm^–1^ region. IRSEC analysis provided evidence consistent with the product of E2PT processes occurring in the three compounds, **3**, **4**, and **5**, characterized by the protonation of the pyridyl group upon phenol oxidation. The emergence of new IR bands at ∼ 1636 and 1626 cm^–1^ assigned to vibrational modes of the pyridinium cation, is indicative of this process in **3**.^43,49^ Concurrently, the gradual decrease in intensity of bands at 1603 and 1577 cm^–1^, associated with ring-stretching modes of the pyridyl group, further confirms the translocation of two protons (one initially on the phenol oxygen and the other initially on the imidazole) and formation of a chemically stable E2PT product.^39^ A small band growing at ∼1515 cm^–1^ is in the region of the C–O stretching mode of the phenoxyl radical.^22^ Compound **4** exhibits a similar trend with growing new bands at ∼1636, 1616, and 1534 cm^–1^ associated with the protonated pyridine, a decrease of the bands at 1596 and 1564 cm^–1^ due to the neutral pyridine moiety, and a small growing band at ∼1515 cm^–1^ in the region of the C–O stretching of the phenoxyl radical. Compound **5** exhibited growing bands at 1637 and 1627 cm^–1^, suggesting again protonation to the pyridine and a decrease of the band at 1603 cm^–1^, corresponding to the neutral pyridine. The band growing at 1510 cm^–1^ is possibly due to the formation of the phenoxyl radical. As observed in a related compound where the hydrogen bond network is interrupted,^39^ the data on compound **5** supports the formation of an E2PT product, which must take place by a two-step process, involving an initial intramolecular proton transfer to the benzimidazole (forming an E1PT product) most likely followed by an intermolecular proton transfer to the pyridine, yielding an E2PT product. A search for the benzimidazolium ion signal was conducted to infer the presence of an E1PT product by the appearance of a band at ∼1556 cm^–1^, which corresponds to the in-plane bending vibrational mode of the NH of the benzimidazolium ion; a band in this region was not found in the IRSEC of **3**, **4**, and **5** consistent with the formation of an E2PT product in every case.^19^

**Figure 4:**
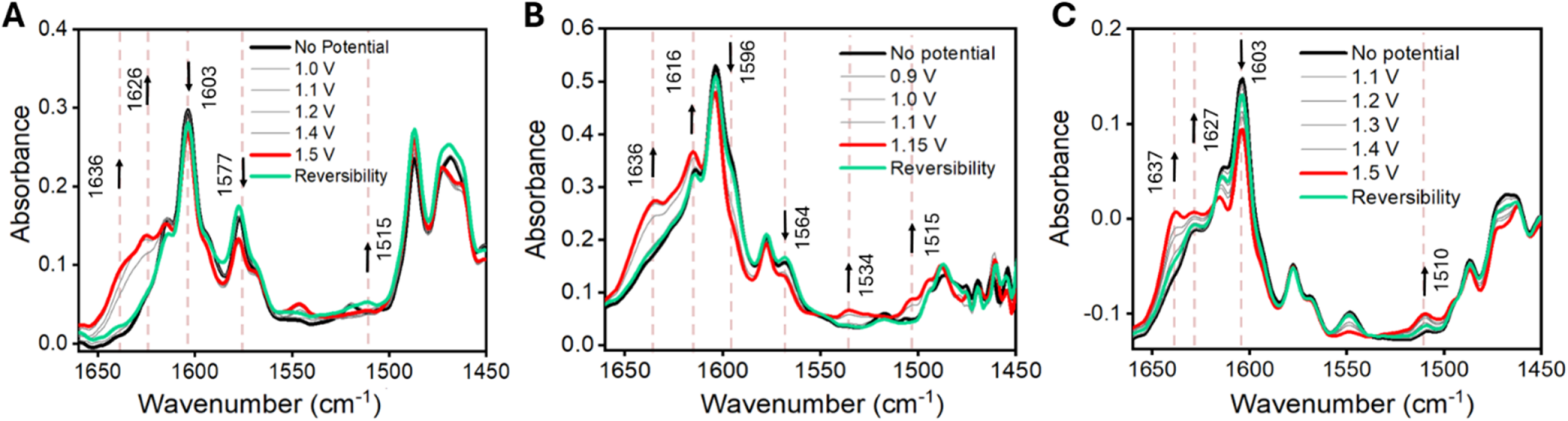
IRSEC spectra of **3**–**5** (8 mM) in MeCN solution containing 0.1 M nBu_4_NPF_6_ as supporting electrolyte at a resolution of 2 cm^–1^. Panels A, B, and C display the oxidative species of **3**, **4**, and **5**, respectively, within the 1650–1450 cm^–1^ region. The black line represents the spectrum at resting potential (no polarization), the red line corresponds to the oxidized state, the green line indicates reversibility, and the gray lines depict spectral evolution during polarization.

Finally, IRSEC control experiments with compound **1** exhibited no significant changes in the 1550–1500 cm^–1^ region upon electrochemical oxidation. In contrast, the electro-oxidation of compound **2** resulted in the formation of new IR bands characteristic of phenoxyl radical formation and intramolecular PCET-induced benzimidazole nitrogen protonation. Specifically, the bands at ∼ 1556 cm^–1^ associated with the NH in-plane bending mode of the benzimidazolium ion and at ∼1515 cm^–1^ of the ν_7a_(C–O) mode of phenoxyl radical were detected.^22^ The Supporting Information (Section S1.7) provides experimental specifications for this technique and details the photocatalysts’ reductive species of **3**–**5** in the 1650–1450 cm^–1^ region.

The ability to reverse complex processes through oxidation–reduction cycles, as illustrated by the green lines in Figure 4, is a crucial attribute for artificial photosynthesis systems. This level of reversibility, a significant hurdle in mimicking natural photosynthetic constructs, suggests the system’s potential applicability in energy conversion technologies.

### 3.5 Visible Spectroelectrochemistry (VISSEC)

VISSEC was conducted to electrochemically characterize the oxidized and reduced species of photocatalysts **3**–**5** in the visible region (Section S1.8). Comparing the transient absorption features observed in transient absorption spectroscopy (TAS) with the known spectra of the electrochemically generated species allows for the confident assignment of redox states to transient intermediates, ultimately aiding in the elucidation of reaction mechanisms and the role of specific redox events. In these Ir­(III) complexes, the reduced species dominate the characteristics of the excited state, as shown in the TAS spectra (Section S1.9).

### 3.6 Visible Transient Absorption Spectroscopy

Photoexcitation of compounds **3**–**5** exhibits similar excited-state absorption features to those of **2**, characterized by a broad maximum at 520–555 nm and a significantly wider band between 600 and 760 nm in both MeCN and THF. These excited species rise at around 2 ps (Figure S41) and decay back to the ground state with lifetimes within 120 ns in MeCN (Figure S42). The similarity of the transient absorption spectra of **3**–**5** to the reduced states of **1** and **2** suggests that the negative charge localizes on the bpy­(π*) LUMO. Also, the resemblance of the transient absorption spectra of **3**–**5** to that of **2** suggests that an internal charge-separated state is formed by a PCET process in <400 fs followed in a few ps by an LLCT process. Together, these processes form the pyridinium ion/phenoxyl radical, and the radical anion localized on the bipyridine ligand (see Scheme S4).^22^

### 3.7 Limited Charge Recombination

The catalytic performance of the novel Ir-BIP-Py catalysts was evaluated in the decarboxylative reduction of *N*-hydroxyphthalimide ester **S**, a reaction commonly used for generating alkyl radicals.^50–57^ As illustrated in Figure 5A, the formation of alkyl radical intermediate occurs upon single electron transfer to phthalimide ester **S** with concomitant bond cleavage to release phthalimide anion and carbon dioxide. While acid additives are commonly used to enhance the rate of this fragmentation, their omission in this experiment was crucial to ensure that fragmentation occurs in kinetic competition with CR, as well as to avoid protonation of the BIP or BIP-Py ligand. Upon irradiation of these solutions with a 440 nm light source over 5 h, initial rate measurements revealed the following trend in increasing reaction rate: **5** < **1** < **2** < **3** < **4** (Figure 5B, see Section S2.1.3 for detailed initial rate values). The internal quantum yields were calculated to be 4.0 × 10^–4^, 1.2 × 10^–3^, 1.5 × 10^–3^, 2.6 × 10^–3^, and 3.1 × 10^–3^ for catalysts **5**, **1**, **2**, **3**, and **4**, respectively. Relative to catalyst **1**, the quantum yields of catalysts **3** and **4** represent enhancements of 118% and 157%, respectively (Figure 5C). Using the quantum yield measurements in combination with excited-state lifetime quenching data, charge recombination ratios were calculated for **1**–**5** (Section S2.3). Relative to catalyst **1**, catalyst **3** afforded a ∼28-fold reduction in the charge recombination rate, while catalyst **4** delivered the most pronounced effect, suppressing charge recombination by approximately 106-fold.

**Figure 5:**
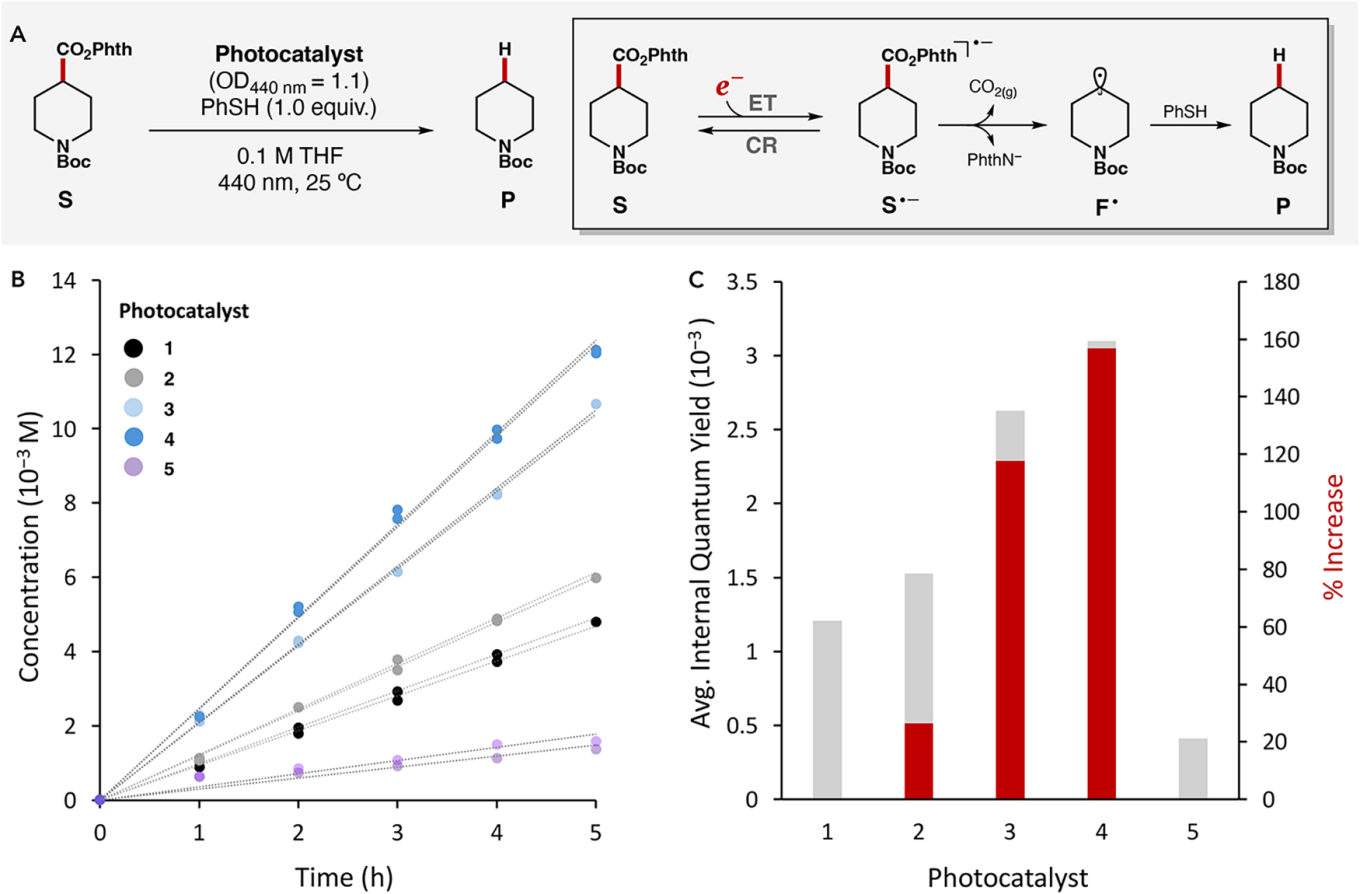
(A) Photoexcitation of the iridium-based photocatalysts triggers electron transfer (ET) to the *N*-hydroxyphthalimide ester substrate (**S**), generating its corresponding radical anion (**S** ^•–^). This intermediate can subsequently either fragment to yield the desired product (**P**) or undergo CR with the oxidized photocatalyst. (B) Comparative analysis of initial rate for the formation of **P** and (C) Left *y*-axis (bars): quantum yield for the reduction of **S**, catalyzed by photocatalysts **1–5**; right *y*-axis (red overlay): % increase in quantum yield for each catalyst with respect to **1**. Conditions: 0.3 mmol **S** (1.0 equiv 0.1 M THF); photocatalyst OD_440 nm_ = 1.1; 1.0 equiv thiophenol; 40 W, 440 nm Kessil lamp with attached 440 nm bandpass interference filter, FWHM of 10 nm; 25 °C. Product yield was determined by GC-FID with an internal standard. Quantum yields are the average of two trials.

Control experiments from our previous study^22^ established that fragmentation of the radical anion intermediate (**S** ^•–^) is the turnover-limiting step under standard catalytic conditions. This mechanistic assignment provides a critical foundation for interpreting improvements in quantum efficiency across the new catalyst series. Specifically, initial rate measurements showed a zero-order dependence on thiophenol concentration for both parent photocatalysts, indicating that neither hydrogen atom transfer (HAT) nor catalyst turnover is kinetically relevant. Additionally, solvent kinetic isotope effect (KIE) studies revealed only a minor effect (*k*_THF_/*k*_THF‑d8_ = 1.2), indicating that solvent participation is not significant in the rate-determining step. The reaction rate was also found to be linear with respect to light intensity, ruling out two-photon excitation as a contributing factor. Together, these findings suggest that fragmentation is the rate-limiting step and support the hypothesis that charge recombination competes with product-forming bond scission. Therefore, the enhanced catalytic performance observed for the new BIP-Py photocatalysts can be attributed to reduced charge recombination, which allows the longer-lived intermolecular charge-separated state to access the productive fragmentation pathway more effectively.

## 4 Conclusion

In summary, we have developed and characterized a new family of heteroleptic Ir­(III) photocatalysts bearing BIP-Py ligands, which are engineered to support concerted two-proton, one-electron transfer via an extended hydrogen-bond network, serving as a complementary platform for improving the quantum efficiency of photoredox reactions. By strategically introducing a pyridinyl terminal proton acceptor at C7 of the BIP core of **2**, we achieved favorable modulation of the potential for phenol oxidation and the generation of characterizable charge-separated states, featuring greater spatial separation of the oxidized and protonated sites. Relative to catalyst **1**, catalyst **3** delivered a ∼28-fold reduction in charge-recombination rate, while catalyst **4** suppresses recombination by ∼106-fold. In the case of **4**, this pronounced decrease in recombination directly translates to enhanced quantum yields, up to a 157% increase versus **1** and 103% relative to the parent Ir-BIP, **2**. Together, these results establish multiproton, redox-relay ligand platforms as an effective strategy to decouple forward electron transfer from deleterious back-electron transfer, thereby pushing the boundaries of photoredox quantum efficiency. We anticipate that applying this MPCET-inspired design framework to other transition-metal and organic photosensitizers will unlock new, low-energy transformations with improved efficiency.

## Supplementary Material



## Notes

Experimental details, electrochemistry, spectroscopic studies, and reaction characterization, NMR data (PDF)
