# Supplementary material for: Leveraging Multiproton-Coupled Electron Transfer to Improve Ir(III) Photocatalyst Efficiency
Source: J Phys Chem C Nanomater Interfaces. 2026 Feb 5;130(7):2492–500. doi: 10.1021/acs.jpcc.5c08623 (PMC12927018; doi:10.1021/acs.jpcc.5c08623)
Supplement: Supplementary file 1 [file jp5c08623_si_001.pdf]

Supplementary Information

Leveraging Multi-Proton-Coupled Electron Transfer to Improve Ir(III) Photocatalyst Efficiency

Eris Villalona[a], Rodrigo E. Domínguez[b], Edwin J. Gonzalez Lopez[b] ,Walter D. Guerra[b], Daniel A. Heredia[c], Anton Y. Khmelnitskiy[b], Daniel G. Oblinsky[a], Yohana Palacios[b], Thomas A. Moore[b], Gregory D. Scholes[a], Robert R. Knowles[a]*, and Ana L. Moore[b]*

[a] Department of Chemistry, Princeton University, Princeton, New Jersey 08544, United States.
[b]School of Molecular Sciences, Arizona State University, Tempe, Arizona 85287, United States.
[c]Instituto para el Desarrollo Agroindustrial y de la Salud (IDAS-CONICET). Departamento de Química, Facultad de Ciencias Exactas, Físico-Químicas y Naturales, Universidad Nacional de Río Cuarto, Ruta Nacional 36 Km 601, X5804BYA Río Cuarto, Córdoba, Argentina

* Correspondence should be addressed to Robert Knowles at rknowles@princeton.edu and Ana Moore at amoore@asu.edu

Table of Contents

1. Experimental Section S2
1.1. Materials and Methods S2
1.2. Synthesis and Structural Characterization S3
1.3. Nuclear Magnetic Resonance (NMR) and Mass Spectrometry (HRMS) Data S12
1.4. Steady-State Absorption Spectroscopy S30
1.5. Steady-State Emission Spectroscopy S30
1.6. Electrochemistry Data S31
1.7. Infrared Spectroelectrochemistry S34
1.8. Visible Spectroelectrochemistry S35
1.9. Ultrafast and Nanosecond Visible Transient Absorption Spectroscopy S37
2. Preparative Scale Photoredox Catalysis S43
2.1. Quantum Yield Determination S44
2.1.1. Measurement of Photon Flux using Ferrioxalate Actinometry S44
2.1.2. Data Analysis for Photon Flux Measurement S45
2.1.3. Initial Rates Kinetics for Quantum Yield Determination at 440 nm S45
2.1.4. UV/VIS Absorption spectra of Photocatalysts at Preparative Scale Concentrations S49
2.1.5. Calculation of Internal Quantum Yield S49
2.2. Catalytic Cycle S50
2.3. Determination of Charge Recombination Ratios S52

2.4. Static vs. Dynamic Quenching of the Excited State of 4 .... S54
3. References S55

1. Experimental Section

1.1. Materials and Methods

All chemicals were purchased from commercial sources unless otherwise noted. Reagents were obtained from Sigma-Aldrich, Combi-Blocks, Alfa Aesar, Oakwood Chemical, and Thermo Fisher Scientific, while solvents were acquired from VWR and Combi-Blocks. Acetonitrile (MeCN) was purified by distillation to remove impurities, including water, acetic acid, and other volatile contaminants. Tetrahydrofuran (THF) underwent a distillation process with sodium and benzophenone in an argon atmosphere before its use. Both MeCN and THF were subsequently stored over 3 Å molecular sieves and potassium carbonate and degassed by Freeze-Pump-Thaw technique. Other solvents used in reactions and purification processes were obtained from commercial sources: hexane, ethyl acetate (EtOAc), dichloromethane (DCM), acetone, extra-dry ethanol (EtOH), toluene, nitrobenzene, 2-ethoxyethanol, dimethoxyethane (DME), and extra-dry dioxane were used as received and kept over activated molecular sieves (3 Å).

Thin-layer chromatography (TLC) was performed on Sigma-Aldrich silica gel aluminum foil plates with a fluorescent indicator (F-254). Therefore, developed chromatograms were visualized under UV light irradiation (λ = 254 or 355 nm). Normal-phase silica gel column chromatography was carried out using silica gel F60 (230-400 mesh) from Silicycle.

Reaction progress was monitored using a Bruker microflex LRF matrix-assisted laser desorption/ionization time-of-flight (MALDI-TOF) mass spectrometer. The spectra were acquired in positive ion reflector mode using *trans,trans*-1,4-diphenyl-1,3-butadiene as the matrix.

High-resolution mass spectra (HRMS) were acquired on an Agilent 6530 Accurate-Mass Q-TOF LC-MS system equipped with an electrospray ionization (ESI) source operating in positive ion mode. A sample of each compound was dissolved in 100 μL of dry DCM and diluted to 1 mL with HPLC-grade methanol. One microliter of each sample was introduced directly into the mass spectrometer without chromatographic separation (no column was employed) using 0.1% formic acid in methanol as a mobile phase at a flow rate of 0.4 mL/min. Mass spectrometer instrument parameters were optimized as follows: capillary voltage 3500 V, gas temperature 300 °C, drying gas flow rate 8 L/min, nebulizer pressure 35 psi, sheath gas temperature 350 °C, sheath gas flow rate 11 L/min, fragmented voltage 175 V, skimmer voltage 60 V, and octupole RF at 750 V, and the mass range was scanned from 100 to 3000 m/z. Data acquisition

and processing were performed using Agilent MassHunter Data Acquisition and Agilent MassHunter Qualitative Analysis software, versions B.08.00 and B.07.00, respectively.

^{1}H-NMR and ^{19}F-NMR spectra were recorded at 25 °C using a Bruker 500 AVANCE spectrometer (500 MHz), employing standard pulse techniques. Samples were dissolved in deuterated chloroform ($CDCl_3$), and chemical shifts were measured relative to internal tetramethylsilane (TMS) standard (0.05% v/v) and were further referenced to residual solvent signals (^{1}H-NMR: $CHCl_3$ at 7.26 ppm). ^{1}H-NMR and ^{19}F-NMR data are reported as follows: chemical shift (ppm), multiplicity (s = singlet, br s = broad singlet, d = doublet, t = triplet, dd = doublet of doublets, and m = multiplet), and coupling constant (Hz).

1.2. Synthesis and Structural Characterization

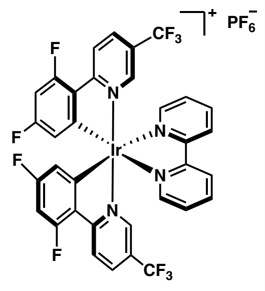

1

[Ir(dF(CF$_3$)ppy)$_2$(bpy)][PF$_6$] (**1**). Compound **1** was prepared according to established literature procedures, and its spectroscopic data were consistent with previously reported values.[1] ^{1}H NMR (500 MHz, acetone-d$_6$): δ 8.90 (d, *J* = 8.2 Hz, 2H), 8.62 (d, *J* = 8.8 Hz, 2H), 8.41 (d, *J* = 7.2 Hz, 4H), 8.30 (d, *J* = 5.2 Hz, 2H), 7.98 (s, 2H), 7.82 – 7.79 (m, 2H), 6.88 – 6.83 (m, 2H), 5.97 (dd, *J* = 8.4, 2.0 Hz, 2H).^{19}F NMR (500 MHz, acetone-d$_6$): δ -63.57 (s, 6F), -71.88 (s, 3F), -73.38 (s, 3F), -104.72 (d, *J* = 12.3 Hz, 2F), -107.97 (d, *J* = 12.4 Hz, 2F). HRMS (ESI) exact mass for $C_{34}H_{18}F_{10}IrN_4^+$ requires m/z = 865.1001; found m/z = 865.1023, (Figures S1-S3).

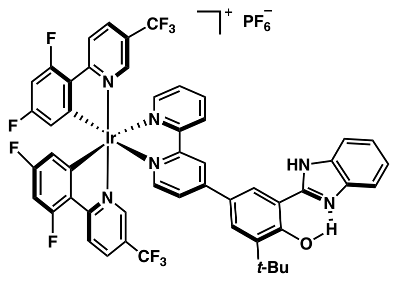

2

[Ir(dF(CF$_3$)ppy)$_2$(BIP-bpy)][PF$_6$] (**2**). Compound **2** was prepared following previously reported procedures.[2] NMR spectroscopic and mass spectrometry data confirmed the structural assignment based on literature.[2] ^{1}H NMR (500 MHz, $CDCl_3$): δ 15.14 (br s, 1H), 10.86 (br s, 1H), 9.20 (d, *J* = 6.9 Hz, 1H), 9.03 (s, 1H), 8.50 – 8.39 (m, 3H), 8.40 (t, *J* = 7.8 Hz, 1H), 8.07 (t, *J* = 9.6 Hz, 2H), 7.95 (d, *J* = 5.0 Hz, 1H), 7.88 (t, *J* = 8.3 Hz, 2H), 7.82 (d, *J* = 4.7 Hz, 1H), 7.77 (s, 1H), 7.70 (d, *J* = 7.7 Hz, 1H), 7.65 (s, 1H), 7.61 – 7.56 (m, 2H), 7.33 – 7.28 (m, 2H), 6.68 (t, *J* = 9.7 Hz, 2H), 5.68 (d, *J* = 7.1 Hz, 2H), 1.51 (s, 9H). ^{19}F NMR (500 MHz, $CDCl_3$): δ -62.71 (s, 3F), -62.87 (s, 3F), -70.17 (s, 3F), -71.69 (s, 3F), -101.18 (d, *J* = 12.5 Hz, 2F), -105.72 (dd, *J* = 38.4, 12.6 Hz, 2F). HRMS (ESI) exact mass for $C_{51}H_{34}F_{10}IrN_6O^+$ requires m/z = 1129.2264; found m/z = 1129.2304, (Figures S4-S6).

The procedure employed for the synthesis of photocatalysts **Ir-BIP-Pyr 3-5** is outlined in Scheme S1, adapting previously reported methods.[2–4] It started with the commercially available brominated *tert*-butyl hydroxybenzaldehyde (**6**) via a Miyaura borylation reaction. This was accomplished using bis(pinacolato)diboron, potassium acetate as a base, and Pd(dppf)Cl_2 as a catalyst in 1,4-dioxane at 110 °C for 3 hours. The resulting boronate ester (**7**) was subsequently coupled with 4-bromo-2,2'-bipyridine through a Miyaura-Suzuki cross coupling reaction catalyzed by $Pd(PPh_3)_4$ and using sodium carbonate as the base. This reaction was carried out in a 1:1 mixture of 1,2-dimethoxyethane (DME) and water at 90 °C for 18 hours, getting the compound (**8**) in a 90% yield.

The iridium(III) dimer (**10**) was prepared by heating a mixture of the precursor (**9,** dF(CF_3)ppy) and $IrCl_3$•nH_2O in 2-ethoxyethanol/water (1:1) at 90 °C overnight. Subsequent cleavage of the dimer was achieved by stirring a mixture of (**10**), silver hexafluorophosphate ($AgPF_6$), DCM, and acetonitrile at 55 °C overnight, also affording the iridium acetonitrile complex (**11, Ir-MeCN**) in excellent yield. The iridium-aldehyde intermediate complex (**12**, **Ir-CHO**) was obtained by coordinating the ligand (**8**) to the iridium acetonitrile complex (**11**) in anhydrous DCM at 40 °C for 48 hours.

Commercially available 4-bromobenzo[c][1,2,5]thiadiazole (**13**) was converted to the corresponding boronate ester (**14**) via a Miyaura borylation reaction following the conditions described above. Subsequent Suzuki-Miyaura cross-coupling reaction with the appropriate pyridine derivatives, following established protocols, afforded the pyridinyl-benzothiadiazoles **15a**, **15b**, and **15c** in 40%, 65%, and 46% yield, respectively[3]. The unstable diaminobenzene derivatives (**16a-c**) were prepared through sulfur extrusion of the **Py-BTD** intermediates (**15a-c**) using a reported method[3]. This reduction involved treatment with sodium borohydride and cobalt chloride under reflux in a mixture of ethanol and THF for 3 hours.

Finally, the synthesis of target photocatalysts **Ir-BIP-Pyr (3-5)** was completed through a Phillips-Ladenburg condensation[3,5,6]. This final step involved the reaction between the **Ir-CHO** intermediate (**12**) and the corresponding diaminobenzene derivative (**16a-c**) in nitrobenzene at 90-110 °C for 48 hours. Photocatalysts **Ir-BIP-Py 3-5** were isolated as yellow-orange solids in 26%, 43%, and 39% yield, respectively.

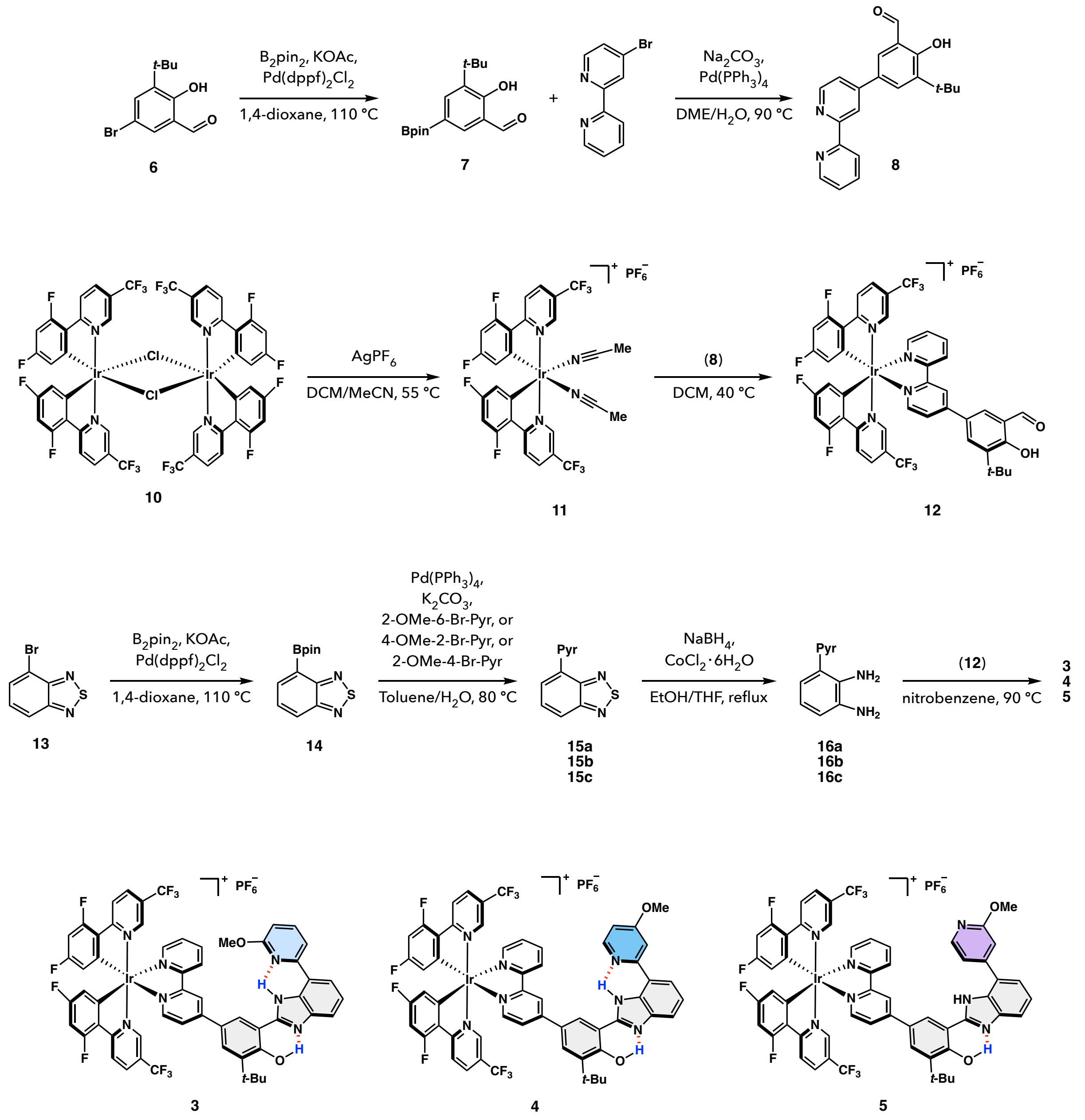

Scheme S1. Synthetic route employed for the synthesis of photocatalysts **3-5.**

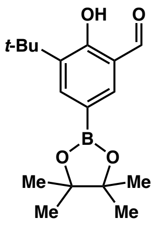

7

3-(*Tert*-butyl)-2-hydroxy-5-(4,4,5,5-tetramethyl-1,3,2-dioxaborolan-2-yl)benzaldehyde (7). The title compound was prepared according to a previously reported procedure[2]. The boronic ester intermediate (**7**) was synthesized via a Miyaura borylation of commercially available 5-bromo-3-(tert-butyl)-2-hydroxybenzaldehyde (**6**, 1 g, 3.88 mmol). This reaction was carried out using [1,1'-bis(diphenylphosphino)ferrocene] dichloropalladium(II), ($Pd(dppf)Cl_2$, 102 mg, 0.14 mmol) as the catalyst, potassium acetate (1.076 g, 10.96 mmol) as the base, and bis(pinacolato)diboron (1.086 g, 4.28 mmol) in 1,4-dioxane (16 mL) under argon atmosphere at reflux for 3 hours. Purification of the crude reaction mixture by flash chromatography (silica gel, hexanes/EtOAc = 95:05 to 80:20) afforded compound **7** (0.836 g, 2.74 mmol) in 70% yield as a white solid. ^{1}H NMR (500 MHz, $CDCl_3$): δ 11.99 (s, 1H), 9.90 (s, 1H), 7.91 (d, J = 11.3 Hz, 2H), 1.44 (s, 9H), 1.35 (s, 12H). HRMS (ESI) exact mass for $C_{17}H_{26}BO_4^+$ requires m/z = 305.1924; found m/z = 305.1936, $(M+H)^+$ (Figures S7 and S8).

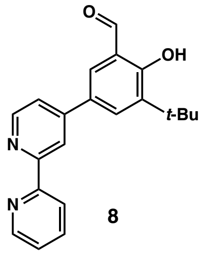

8

5-([2,2'-Bipyridin]-4-yl)-3-(*tert*-butyl)-2-hydroxybenzaldehyde (8). The title compound was prepared according to a previously reported procedure[2]. A mixture of boronic ester **7** (320 mg, 1 mmol), 4-bromo-2,2'-bipyridine (235 mg, 1 mmol), Na_2CO_3 (1 mL of a 2 M aqueous solution, 0.98 mmol), and $Pd(PPh_3)_4$ (24 mg, 0.02 mmol) in 1,2- dimethoxyethane (DME, 16 mL) and water (4 mL) was stirred under argon at reflux for 18 h. The reaction mixture was dried under reduced pressure, diluted with water (30 mL) and DCM (90 mL), and extracted with EtOAc (3x50 mL). The combined organic layers were dried over $MgSO_4$ and purified by column chromatography (silica gel, hexanes/EtOAc = 50:50 to 20:80) to yield compound **8** (300 mg, 0.88 mmol, 94%) as a pale brown solid. ^{1}H NMR (500 MHz, $CDCl_3$): δ 11.94 (s, 1H), 10.01 (s, 1H), 8.73 (t, J = 4.4 Hz, 2H), 8.64 (d, J = 1.0 Hz, 1H), 8.47 (d, J = 8.0 Hz, 1H), 7.90 (d, J = 2.1 Hz, 1H), 7.88 – 7.83 (m, 2H), 7.51 (dd, J = 5.1, 1.7 Hz, 1H), 7.35 (dd, J = 6.6, 5.0 Hz, 1H), 1.50 (s, 9H). HRMS (ESI) exact mass for $C_{21}H_{21}N_2O_2^+$ requires m/z = 333.1603; found m/z = 333.1627, $(M+H)^+$ (Figures S9 and S10).

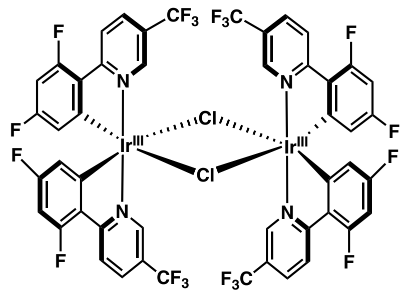

10

Ir(III) μ$_2$-chloride dimer (10). The title compound was prepared according to a previously reported procedure.[2] Ir(III) μ$_2$-chloride dimer (**10**) was prepared by refluxing a mixture of 5-(2,4-difluorophenyl)-2-(trifluoromethyl)pyridine (3.05 g, 11.8 mmol, 2.05 equiv) and iridium(III) chloride hydrate (1.82 g, 5.7 mmol, 1 equiv) in a degassed 2-ethoxyethanol/water (75/25 mL) solvent system overnight. The resulting precipitate was purified by filtration, washed with water, and dried to afford the target dimer in 86 % yield as a yellow solid. The crude product was carried forward without further purification.

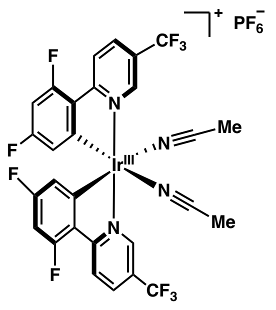

11

[Ir(dF(CF$_3$)ppy)$_2$(MeCN)$_2$][PF$_6$], (Ir-MeCN), (11). The title compound was prepared according to a previously reported procedure.[2] Compound **10** (0.730 g, 0.493 mmol) and silver hexafluorophosphate (0.250 g, 0.989 mmol) were combined in DCM and acetonitrile (12 mL/4 mL). The reaction mixture was stirred at 55 °C under an inert atmosphere overnight. After filtration and concentration compound **11** was afforded as a pale-yellow solid in 85% yield. The crude product was carried forward without further purification.

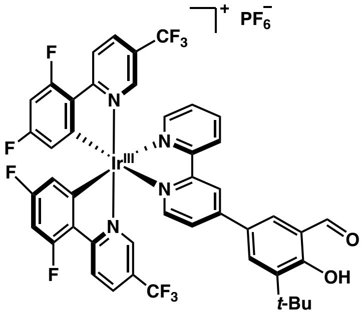

12

Ir-CHO (12). To synthesize the key intermediate **Ir-CHO** (**12**), compound **8** (5-([2,2'-bipyridin]-4-yl)-3-(tert-butyl)-2-hydroxybenzaldehyde, 200 mg, 0.602 mmol, 1 equiv.) and compound **11** (**Ir-MeCN**, 619 mg, 0.662 mmol, 1.1 equiv.) were stirred in anhydrous DCM (20 mL), in the dark at 40 °C for 16 hours. The reaction progress was monitored by mass spectrometry. After 24 hours, an additional 10 mol% of the **Ir-MeCN** precursor was added, and the reaction mixture was stirred for another 24 hours. The crude product was subjected to two consecutive silica gel column chromatography purifications, eluting initially with a gradient of hexane and ethyl acetate (90:10 to 30:70), followed by DCM and methanol (90:10). Subsequently, 3-5 successive recrystallizations using a mixture of hexane and DCM were performed to afford the pure Ir-CHO compound as a bright yellow solid (555 mg, 54%). ^{1}H NMR (500 MHz, $CDCl_3$): δ 12.43 (s, 1H), 10.16 (s, 1H), 9.19 (d, J = 8.2 Hz, 1H), 9.03 (s, 1H), 8.51 (dd, J = 10.5, 4.5 Hz, 2H), 8.41 – 8.34 (m, 2H), 8.07 (t, J = 8.9 Hz, 2H), 7.93 (dd, J = 11.1, 5.5 Hz, 2H), 7.81 (d, J = 1.8 Hz, 1H), 7.76 (d, J = 4.6 Hz, 1H), 7.71 (s, 1H), 7.64 – 7.57 (m, 1H), 7.56 (s, 1H), 6.72 – 6.63 (m, 2H), 5.67 (dd, J = 9.1, 3.7 Hz, 2H), 1.44 (s, 9H). ^{19}F NMR (500 MHz, $CDCl_3$): δ -62.70 (s, 3F), -62.83 (s, 3F), -71.31 (s, 3F), -72.82 (s, 3F), -101.23 (dd, *J* = 49.2, 13.3 Hz, 2F), -105.60 (dd, *J* = 31.6, 13.3 Hz, 2F). HRMS (ESI) exact mass for $C_{45}H_{30}F_{10}IrN_4O_2^+$ requires m/z = 1041.1838; found m/z = 1041.1876, (Figures S11-S13).

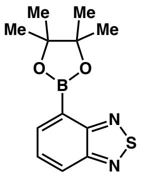

14

(4-(4,4,5,5-Tetramethyl-1,3,2-dioxaborolan-2-yl)benzo[c][1,2,5]thiadiazole (B-BTD) (14) The title compound was prepared according to a previously reported procedure[3]. The borylation of 4-bromobenzo[c][1,2,5]thiadiazole (**13**) was conducted according to the procedure outlined in *Step 1*. Specifically, a mixture of (**13**), (1 g, 4.7 mmol), bis(pinacolato)diboron (1.42 g, 5.6 mmol), potassium acetate (1.33 g, 13.3 mmol), and Pd(dppf)Cl_2 (190 mg, 0.27 mmol) in anhydrous 1,4-dioxane (20 mL) was purged with argon and stirred at 110 °C for 3 hours. The reaction mixture was extracted with water and DCM (3 x 100 mL), washed with saturated $NaHCO_3$ solution and brine, dried over Na_2SO_4, and concentrated under reduced pressure to afford (**14**), (53%) as a dark yellow solid. ^{1}H NMR (500 MHz, $CDCl_3$): δ 8.27 (d, J = 6.9 Hz, 1H), 8.11 (d, J = 8.8 Hz, 1H), 7.80 (dd, J = 8.6, 7.1 Hz, 1H), 1.26 (s, 12H). HRMS (ESI) exact mass for $C_{12}H_{16}BN_2O_2S^+$ requires m/z = 263.1026; found m/z = 263.1052, $(M+H)^+$ (Figures S14-S15).

Suzuki-Miyaura cross-coupling reaction for the synthesis of Py-BTD

The title compounds were prepared according to a previously reported procedure.[3] The Suzuki-Miyaura coupling of the previously synthesized boronic ester **14** (262 mg, 1 mmol) with 4-OMe-2-bromopyridine, 2-OMe-6-bromopyridine, or 2-OMe-4-bromopyridine (1.2 mmol) was performed using palladium tetrakis(triphenylphosphine) ($Pd(PPh_3)_4$, 58 mg, 0.05 mmol) as the catalyst, and potassium carbonate (415 mg, 3 mmol) as the base in a toluene/water (4/1 mL) mixture. The reaction was stirred under argon for 15 minutes at room temperature before heating at 80°C for 48 hours. Standard aqueous workup (extraction using EtOAc, 30 mL x 3), filtration, and evaporation under reduced pressure yielded the desired Py-BTD product (**15a**, **15b**, or **15c** in 40%, 65%, and 46% yield, respectively).

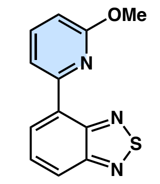

15a

4-(6-Methoxypyridin-2-yl)benzo[c][1,2,5]thiadiazole (15a). ^1H NMR (500 MHz, $CDCl_3$): δ 8.60 (dd, J = 7.1, 1.0 Hz, 1H), 8.47 (d, J = 7.4 Hz, 1H), 8.06 (dd, J = 8.7, 1.0 Hz, 1H), 7.76 (dd, J = 15.9, 8.5 Hz, 2H), 6.80 (d, J = 8.2 Hz, 1H), 4.08 (s, 3H). HRMS (ESI) exact mass for $C_{12}H_{10}N_3OS^+$ requires m/z = 244.0545; found m/z = 244.0564, $(M+H)^+$ (Figures S16-S17).

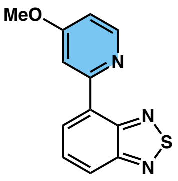

15b

4-(4-Methoxypyridin-2-yl)benzo[c][1,2,5]thiadiazole (15b). ^1H NMR (500 MHz, $CDCl_3$): δ 8.59 (d, J = 5.7 Hz, 1H), 8.45 (d, J = 7.0 Hz, 1H), 8.28 (d, J = 2.3 Hz, 1H), 8.07 (d, J = 8.7 Hz, 1H), 7.74 (dd, J = 8.6, 7.2 Hz, 1H), 6.87 (dd, J = 5.6, 2.4 Hz, 1H), 3.97 (s, 3H). HRMS (ESI) exact mass for $C_{12}H_{10}N_3OS^+$ requires m/z = 244.0545; found m/z = 244.0564, $(M+H)^+$ (Figures S18-S19).

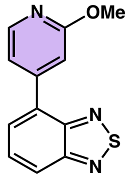

15c

4-(2-Methoxypyridin-4-yl)benzo[c][1,2,5]thiadiazole (15c). ^1H NMR (500 MHz, $CDCl_3$): δ 8.32 (d, J = 5.3 Hz, 1H), 8.08 (d, J = 8.7 Hz, 1H), 7.78 (d, J = 6.8 Hz, 1H), 7.70 (dd, J = 8.5, 7.1 Hz, 1H), 7.45 (dd, J = 5.2, 1.0 Hz, 1H), 7.37 (s, 1H), 4.02 (s, 3H). HRMS (ESI) exact mass for $C_{12}H_{10}N_3OS^+$ requires m/z = 244.0545; found m/z = 244.0563, $(M+H)^+$ (Figures S20-S21).

Sulfur Extrusion

The diaminobenzene derivatives (**16a-c**) were prepared via sulfur extrusion of the corresponding Py-BTD precursors (**15a-c**) according to a previously reported procedure[3]. Briefly, a suspension of the Py-BTD derivative (0.2 mmol) and $CoCl_2{\cdot}6H_2O$ (0.01 mmol, 2.4 mg) in a mixture of ethanol (5 mL) and THF (0.5 mL) was stirred for 15 minutes before the addition of $NaBH_4$ (0.8 mmol, 30 mg). The reaction mixture was heated to reflux at 90 °C for 3 hours, cooled to room temperature, and filtered through Celite. The filtrate was concentrated under reduced pressure, and the residue was extracted with ethyl acetate. The combined

organic layers were dried over $MgSO_4$ and concentrated under reduced pressure to afford the crude diaminobenzene derivatives (**16a-c**), which were used immediately without further purification due to instability.

Phillips-Ladenburg condensation

Finally, the synthesis of photocatalysts **Ir-BIP-Py 3-5** was completed via the Phillips-Ladenburg condensation reaction. To this end, **Ir-CHO** (**12**, 350 mg, 0.3 mmol, 1 equiv.) and the corresponding diamino-derivative (**16a-c**, 75 mg, 0.35 mmol, 1.2 equiv.) were combined in 5 mL of nitrobenzene and stirred under argon for 30 minutes and then heated at 100 °C for 48 - 72 hours. The solvent was removed under reduced pressure, and the residue was purified by silica gel column chromatography (DCM/acetone 100:0 to 80:20). Subsequent multiple recrystallizations from a DCM/hexane mixture afforded the desired photocatalysts **Ir-BIP-Py 3-5** (26%, 43%, and 39% yield, respectively).

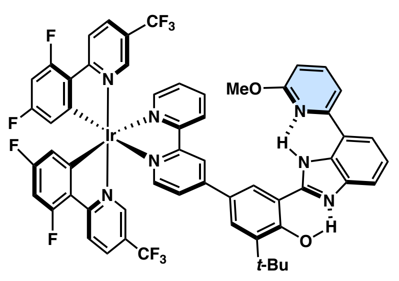

3

[Ir(dF(CF$_3$)ppy)$_2$(BIP-*o*-OMe-*o*-Py)][PF$_6$] (3). ^{1}H NMR (500 MHz, $CDCl_3$): δ 15.27 (s, 1H), 10.94 (s, 1H), 9.17 (d, J = 8.1 Hz, 1H), 9.00 (s, 1H), 8.51 (t, J = 6.9 Hz, 3H), 8.47 (s, 1H), 8.38 (t, J = 7.4 Hz, 1H), 8.07 (m, J = 15.6, 7.3 Hz, 5H), 7.95 (d, J = 5.2 Hz,1H), 7.90 (t, J = 7.1 Hz, 2H), 7.83 – 7.80 (m, 1H), 7.78 (d, J = 7.6 Hz, 1H), 7.66 (s, 1H), 7.61 – 7.56 (m, 2H), 7.43 (t, J = 7.8 Hz, 1H), 6.76 (d, J = 8.2 Hz, 1H), 6.68 (t, J = 10.3 Hz, 2H), 5.68 (d, J = 7.8 Hz, 2H), 4.12 (s, 3H), 1.52 (s, 9H). ^{19}F NMR (500 MHz, $CDCl_3$) δ -62.70 (s, 3F), -62.87 (s, 3F), -70.27 (s, 3F), -71.79 (s, 3F), -101.18 (dd, *J* = 12.5, 4.6 Hz, 2F), -105.72 (dd, *J* = 39.6, 12.6 Hz, 2F). HRMS (ESI) exact mass for $C_{57}H_{39}F_{10}IrN_7O_2^+$ requires m/z = 1236.2635; found m/z = 1236.2649, $(M)^+$ (Figures S22-S24).

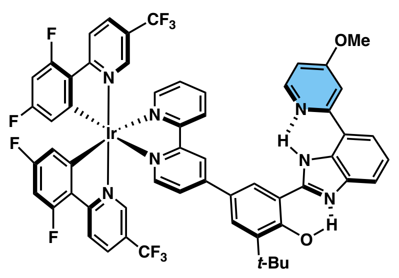

4

[Ir(dF(CF$_3$)ppy)$_2$(BIP-*p*-OMe-*o*-Py)][PF$_6$] (4). ^{1}H NMR (500 MHz, $CDCl_3$): δ 15.35 (s, 1H), 10.98 (s, 1H), 9.18 (d, J = 7.1 Hz, 1H), 9.01 (s, 1H), 8.56 (d, J = 5.7 Hz, 1H), 8.53 – 8.48 (m, 3H), 8.39 (t, J = 7.2 Hz, 1H), 8.22 (s, 1H), 8.12 (d, J = 7.1 Hz, 1H), 8.07 (t, J = 9.5 Hz, 3H), 7.95 (d, J = 3.8 Hz, 1H), 7.91 (t, J = 6.9 Hz, 2H), 7.81 (dd, J = 6.0, 1.2 Hz, 1H), 7.77 (s, 1H), 7.66 (s, 1H), 7.58 (s, 1H), 7.44 (d, J = 7.5 Hz, 1H), 6.84 (dd, J = 5.6, 2.3 Hz, 1H), 6.72 – 6.65 (m, 2H), 5.72 – 5.64 (m, 2H), 4.07 (s, 3H), 1.50 (s, 9H). ^{19}F NMR (500 MHz, $CDCl_3$) δ -63.38 (s, 3F), -63.49 (s, 3F), -71.88 (s, 3F), -73.38 (s, 3F), -104.63 (dd, *J* = 39.9, 12.4 Hz, 2F), -107.95 (d, *J* = 11.1 Hz, 2F). HRMS (ESI) exact mass for $C_{57}H_{39}F_{10}IrN_7O_2^+$ requires m/z = 1236.2635; found m/z = 1236.2651, $(M)^+$ (Figures S25-S27).

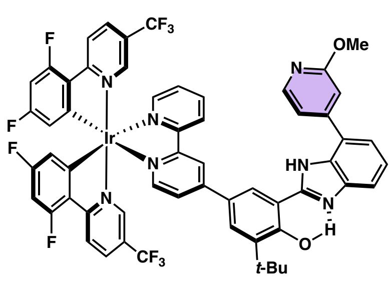

5

[Ir(dF(CF_3)ppy)$_2$(BIP-*o*-OMe-*p*-Py)][PF_6] (5). 1H NMR (500 MHz, CDCl3) δ 14.81 (s, 1H), 11.16 (s, 1H), 9.26 (s, 1H), 9.09 (s, 1H), 8.55 (s, 1H), 8.53 – 8.47 (m, 2H), 8.42 (t, J = 7.6 Hz, 1H), 8.33 (d, J = 5.3 Hz, 1H), 8.07 (t, J = 9.8 Hz, 2H), 7.96 (s, 1H), 7.94 (d, J = 3.7 Hz, 1H), 7.90 (d, J = 6.0 Hz, 1H), 7.83 (dd, J = 6.1, 1.9 Hz, 1H), 7.77 (s, 1H), 7.68 (d, J = 2.3 Hz, 1H), 7.63 – 7.58 (m, 1H), 7.57 (d, J = 5.4 Hz, 2H), 7.48 (d, J = 7.5 Hz, 1H), 7.41 (t, J = 7.9 Hz, 1H), 7.38 (s, 1H), 6.68 (t, J = 10.5 Hz, 2H), 5.68 (d, J = 6.6 Hz, 2H), 4.05 (s, 3H), 1.50 (s, 9H). ^{19}F NMR (500 MHz, $CDCl_3$) δ -62.71 (s, 3F), -62.87 (s, 3F), -70.63 (s, 3F), -72.15 (s, 3F), -101.17 (dd, *J* = 12.5, 4.6 Hz, 2F), -105.67 (dd, *J* = 39.6, 12.6 Hz, 2F). HRMS (ESI) exact mass for $C_{57}H_{39}F_{10}IrN_7O_2^+$ requires m/z = 1236.2635; found m/z = 1236.2583, $(M)^+$ (Figures S28-S30).

1.3. Nuclear Magnetic Resonance (NMR) and Mass Spectrometry (HRMS) Data

Compound **1**

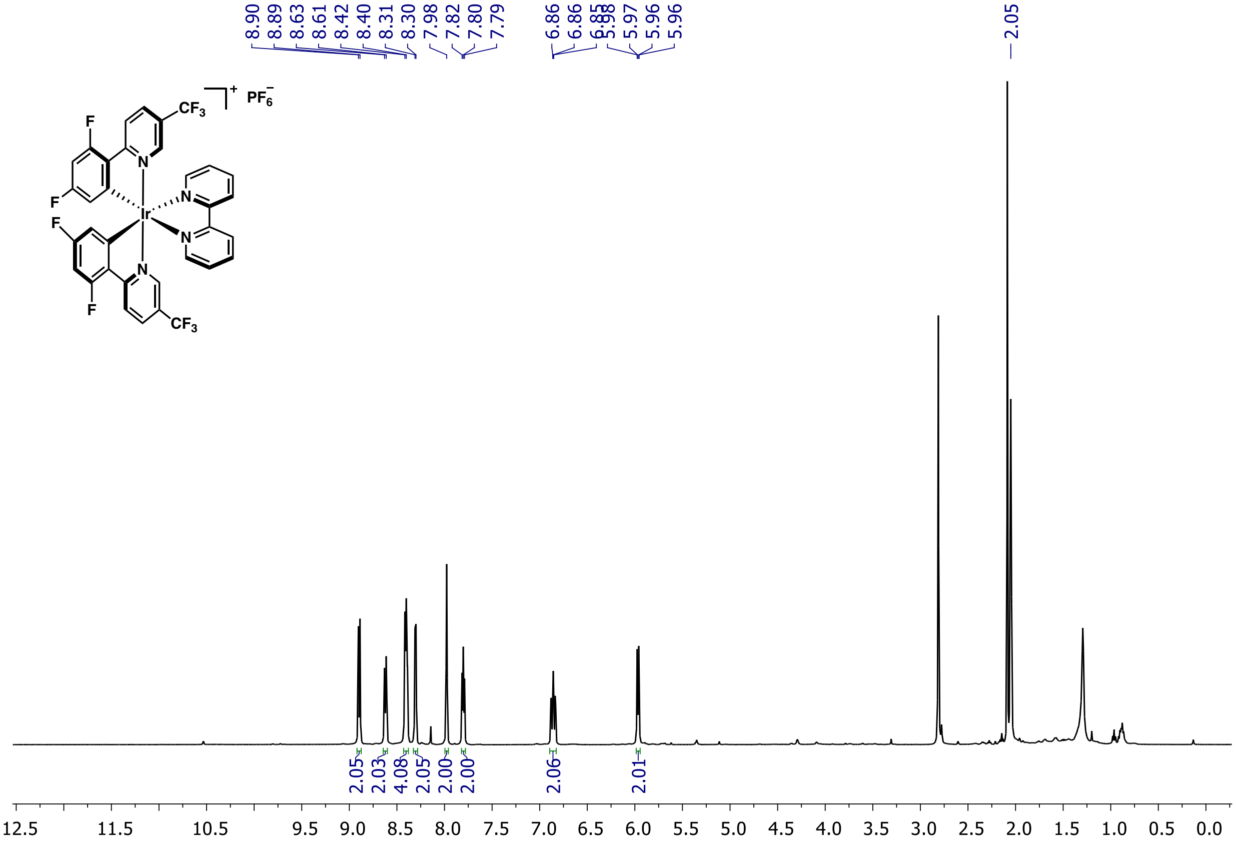

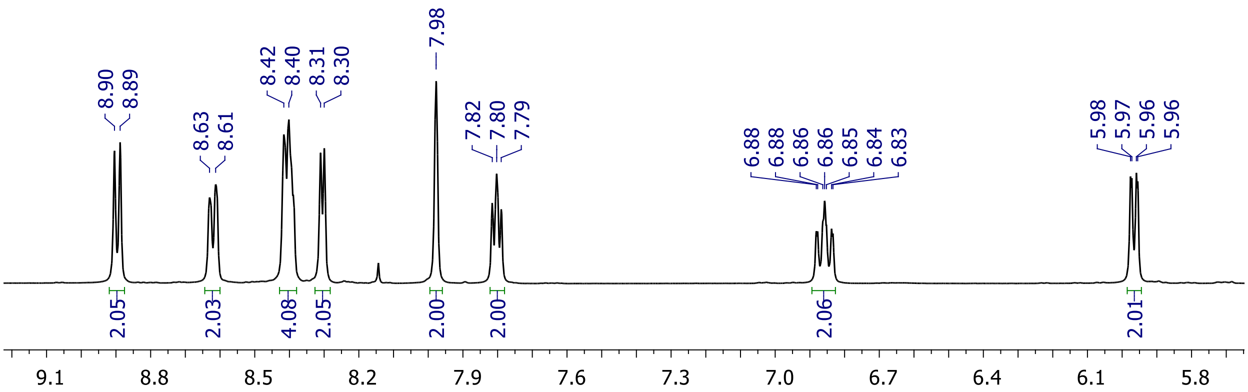

Figure S1. ^{1}H NMR (500 MHz, acetone-d_6) of $[Ir(dF(CF_3)ppy)_2(bpy)][PF_6]$ (**1**). Complete spectrum (top) and expansion of selected regions (bottom).

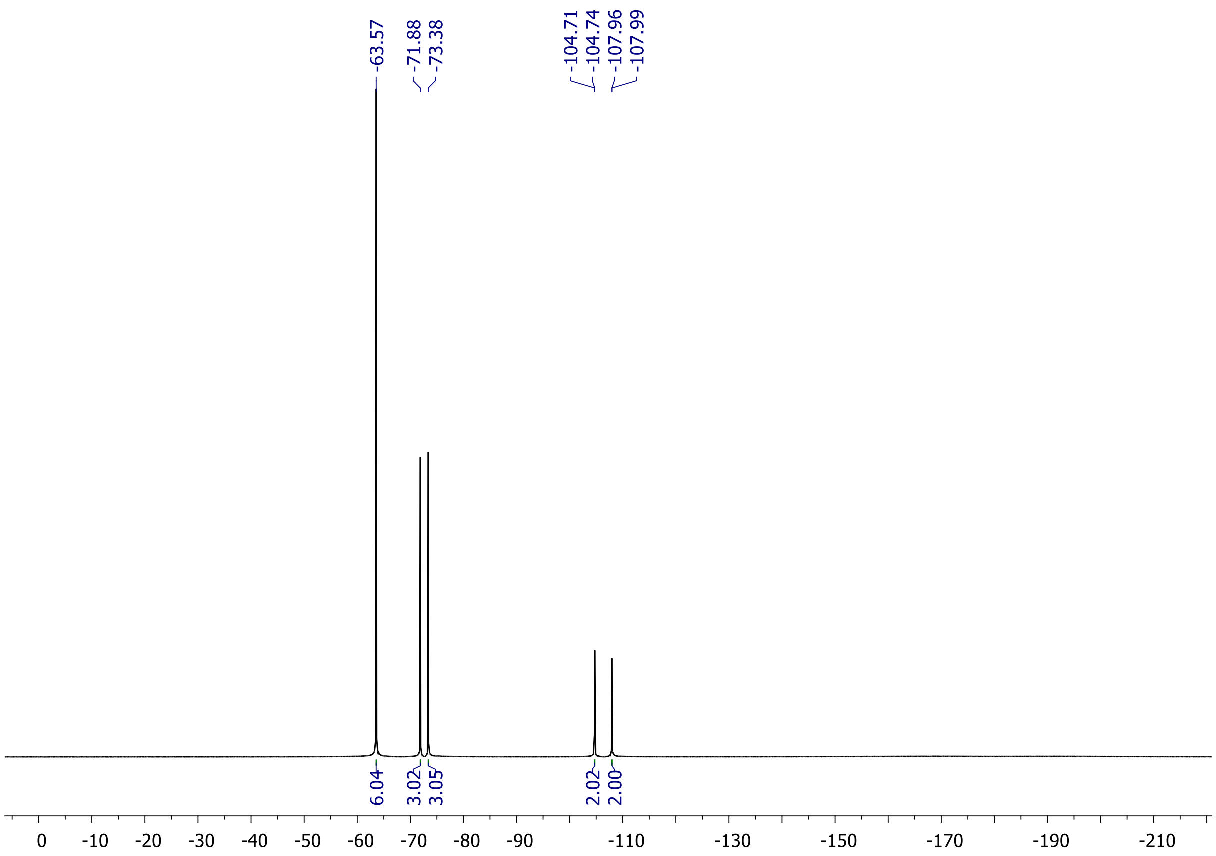

Figure S2. ^{19}F NMR (500 MHz, acetone-d$_6$) spectrum of [Ir(dF(CF$_3$)ppy)$_2$(bpy)][PF$_6$] (**1**).

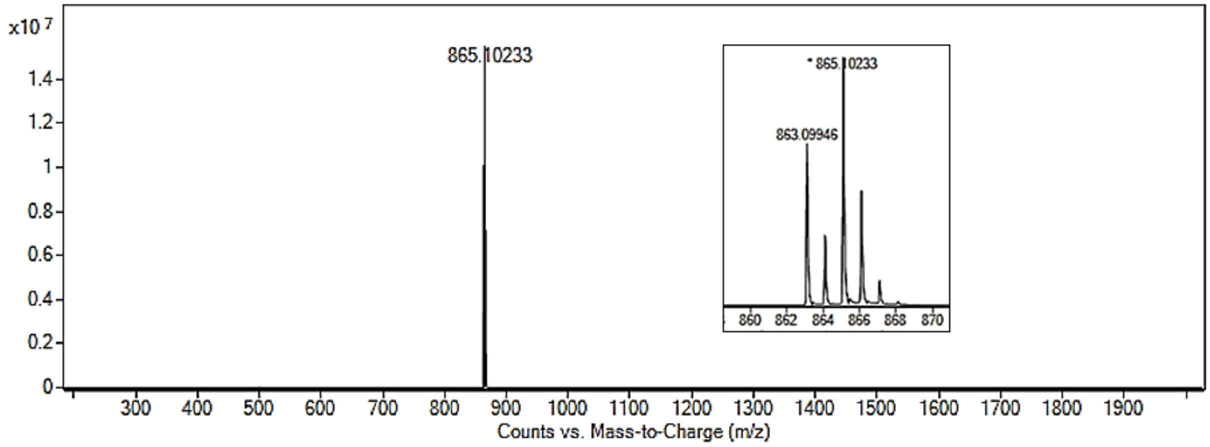

Figure S3. High-resolution mass spectrum of **1**, [Ir(dF(CF$_3$)ppy)$_2$(bpy)]$^+$, (M$^+$).

Compound **2**

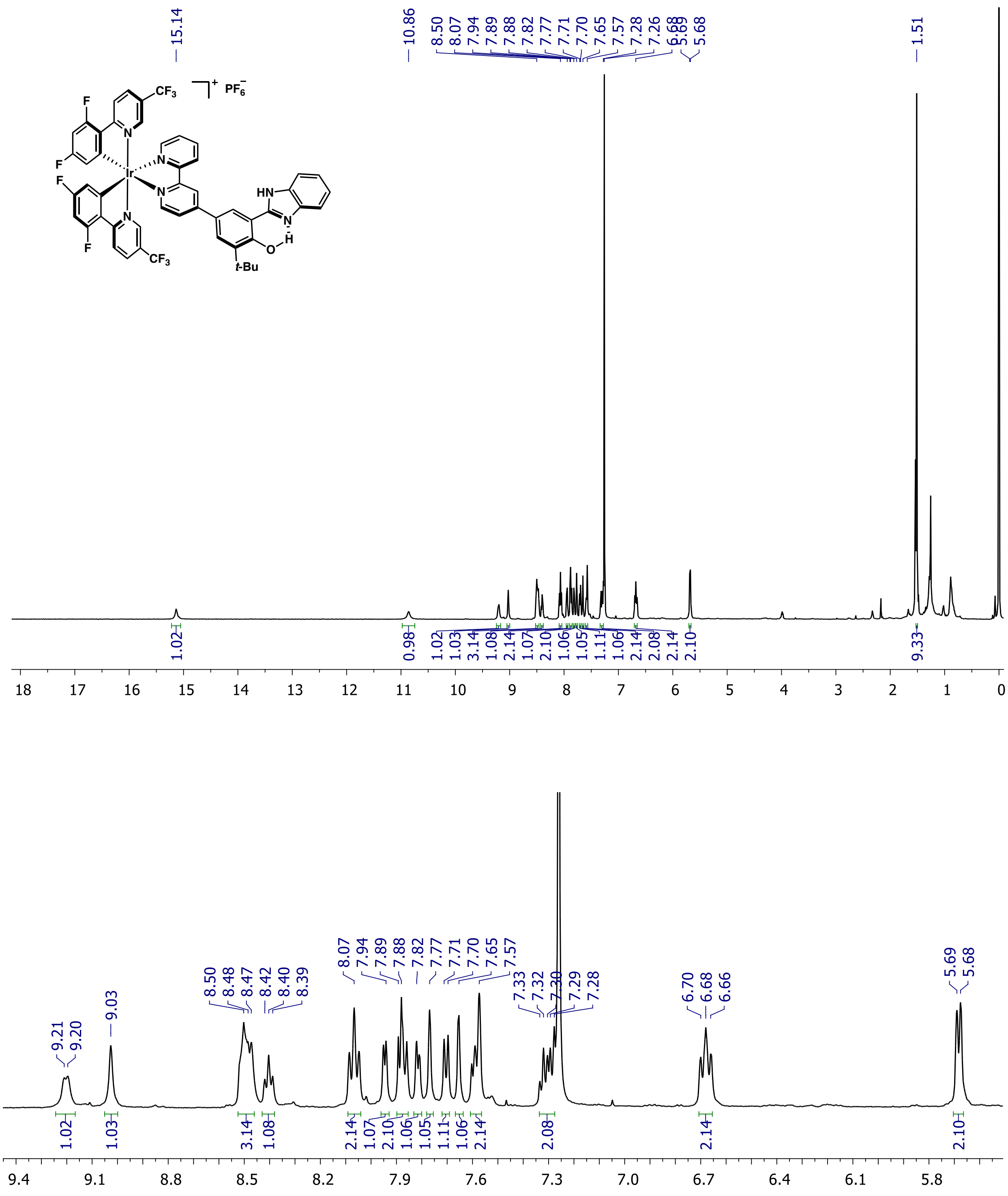

Figure S4. (top) ^{1}H NMR (500 MHz, $CDCl_3$) spectrum of **2**, [Ir(dF(CF_3)ppy)$_2$(BIP-bpy)][PF_6] and (bottom) expansion of selected regions.

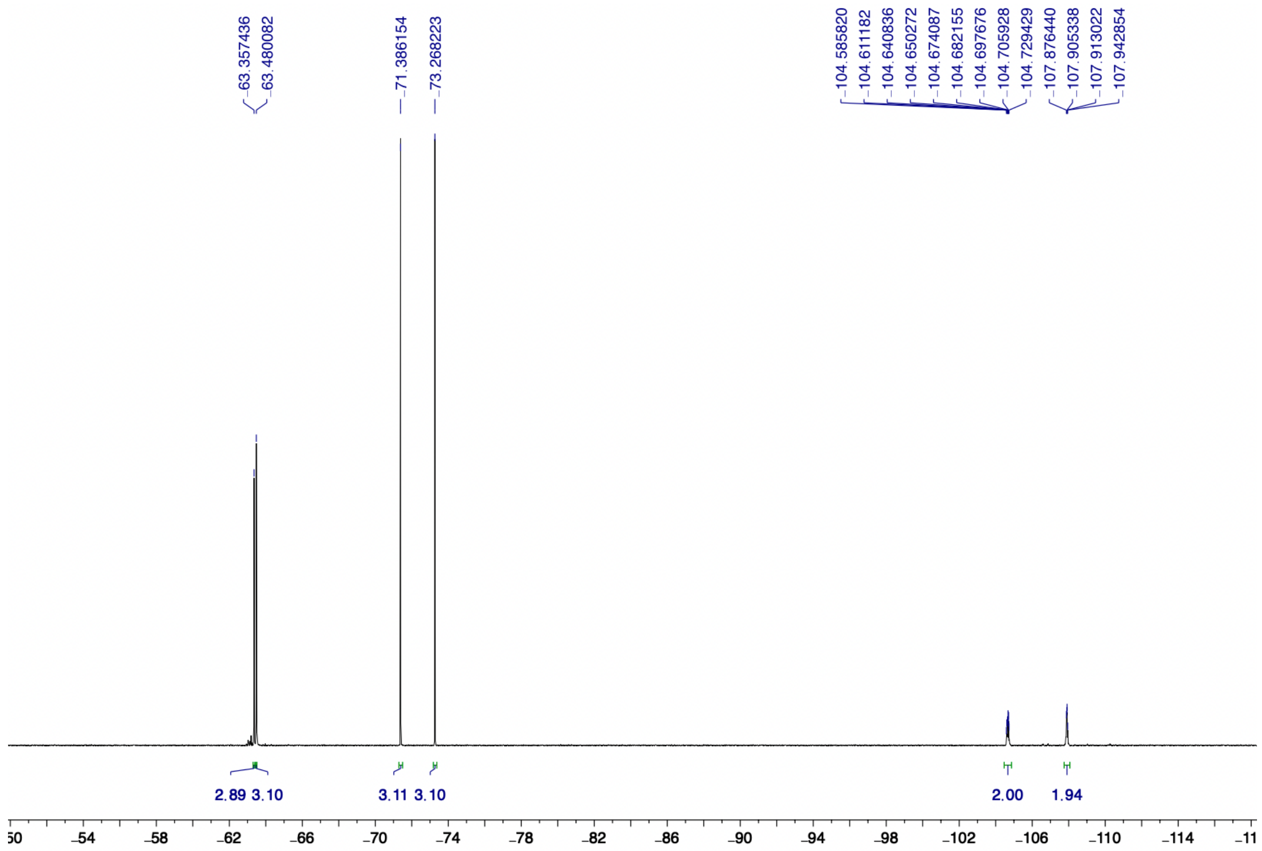

Figure S5. ^{19}F NMR (500 MHz, $CDCl_3$), (top) spectrum of **2**, [Ir(dF(CF_3)ppy)$_2$(BIP-bpy)][PF_6], (insets) expansion of selected regions.

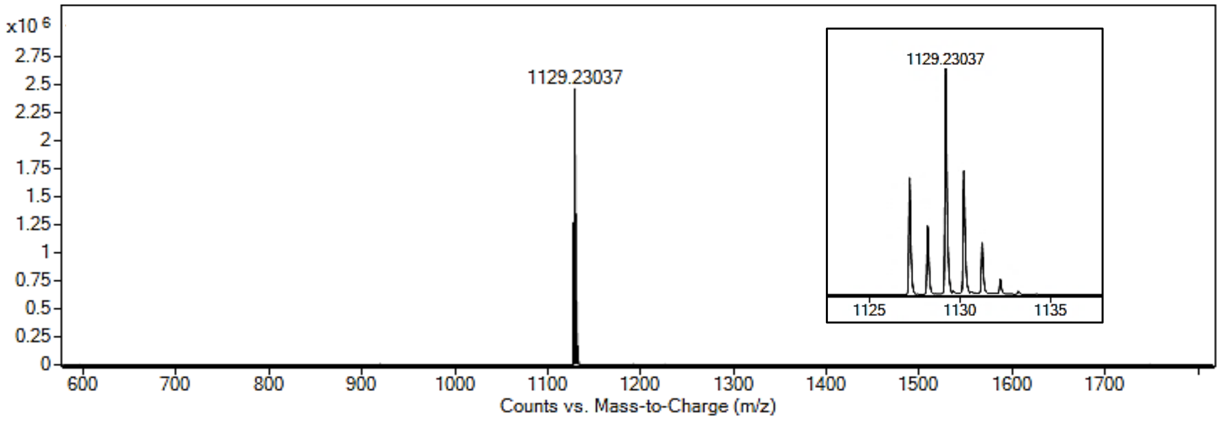

Figure S6. High-resolution mass spectrum of **2**, [Ir(dF(CF_3)ppy)$_2$(BIP-bpy)]$^+$, (M^+).

Compound **7**

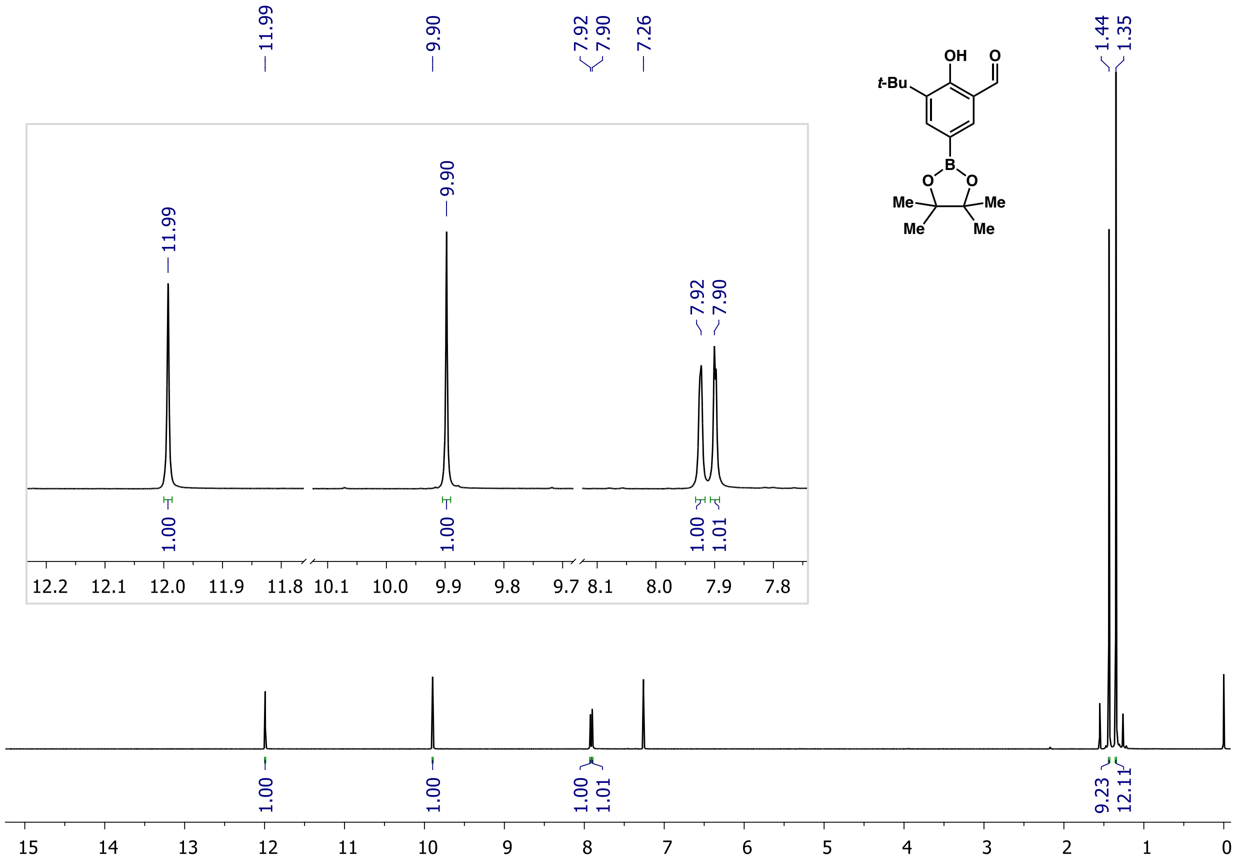

Figure S7. 500 MHz ^{1}H NMR spectrum of **7** in $CDCl_3$, (insets) expansion of selected regions.

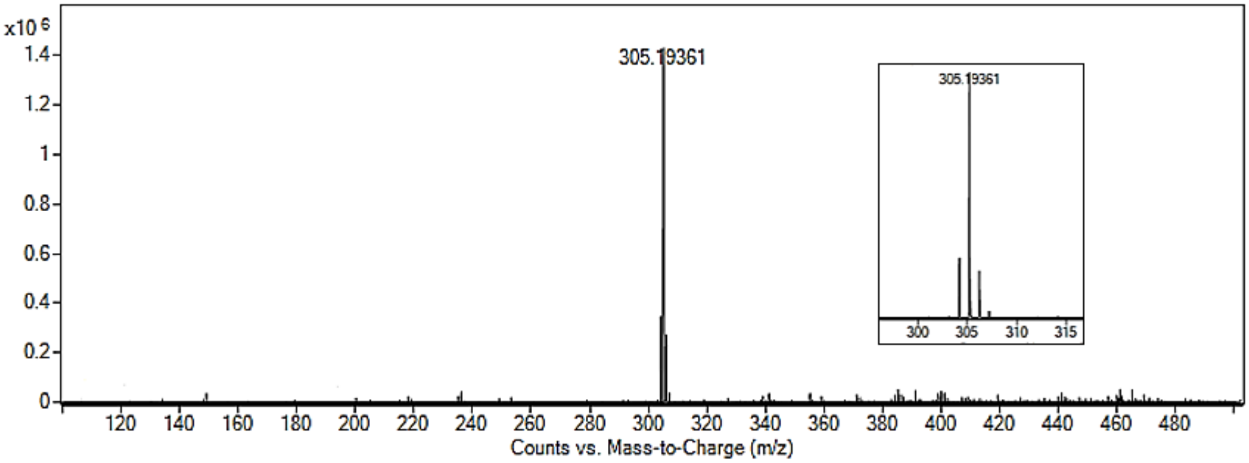

Figure S8. High-resolution mass spectrum of **7**.

Compound **8**

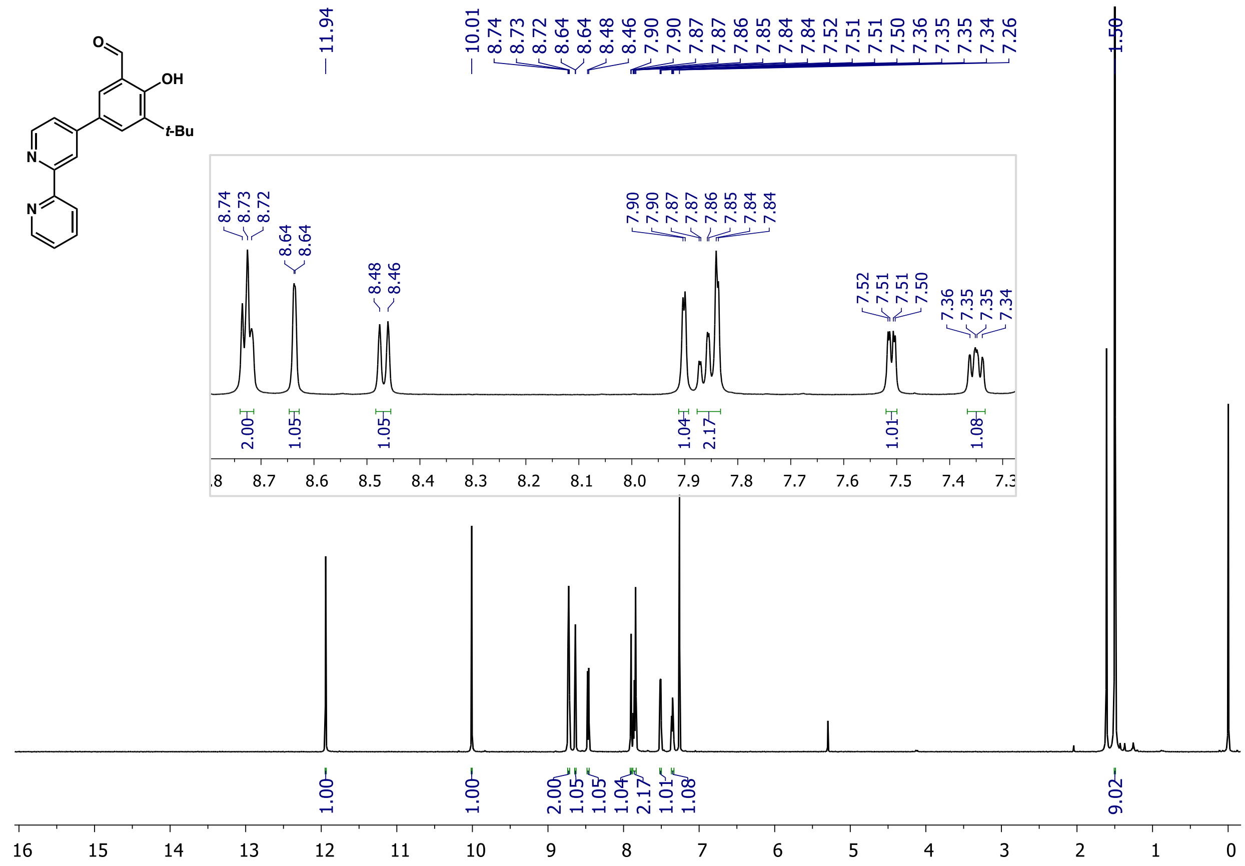

Figure S9. 500 MHz ^{1}H NMR spectrum of **8** in $CDCl_3$, (insets) expansion of selected regions.

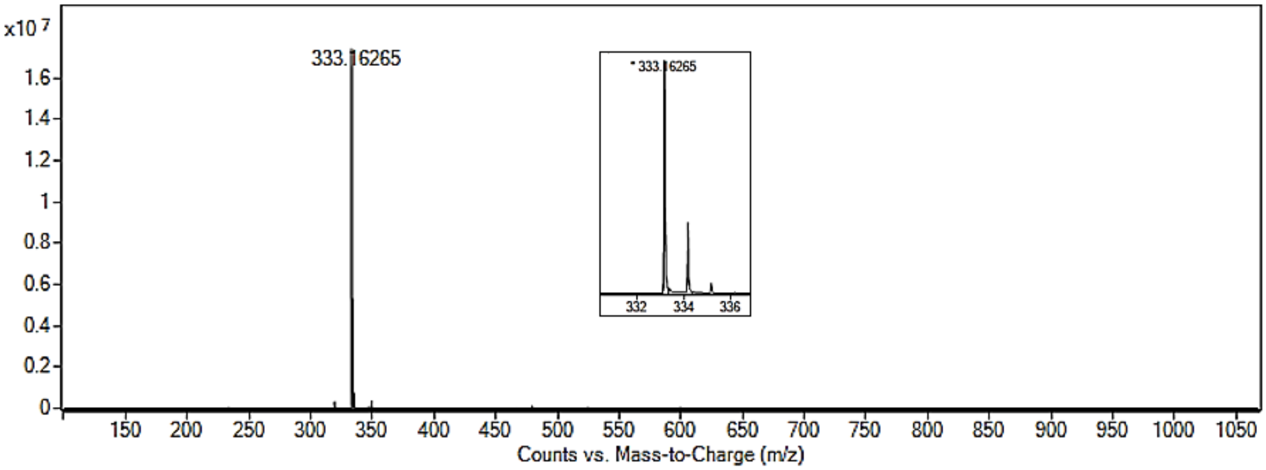

Figure S10. High-resolution mass spectrum of **8**.

Compound **12** (**Ir-CHO)**

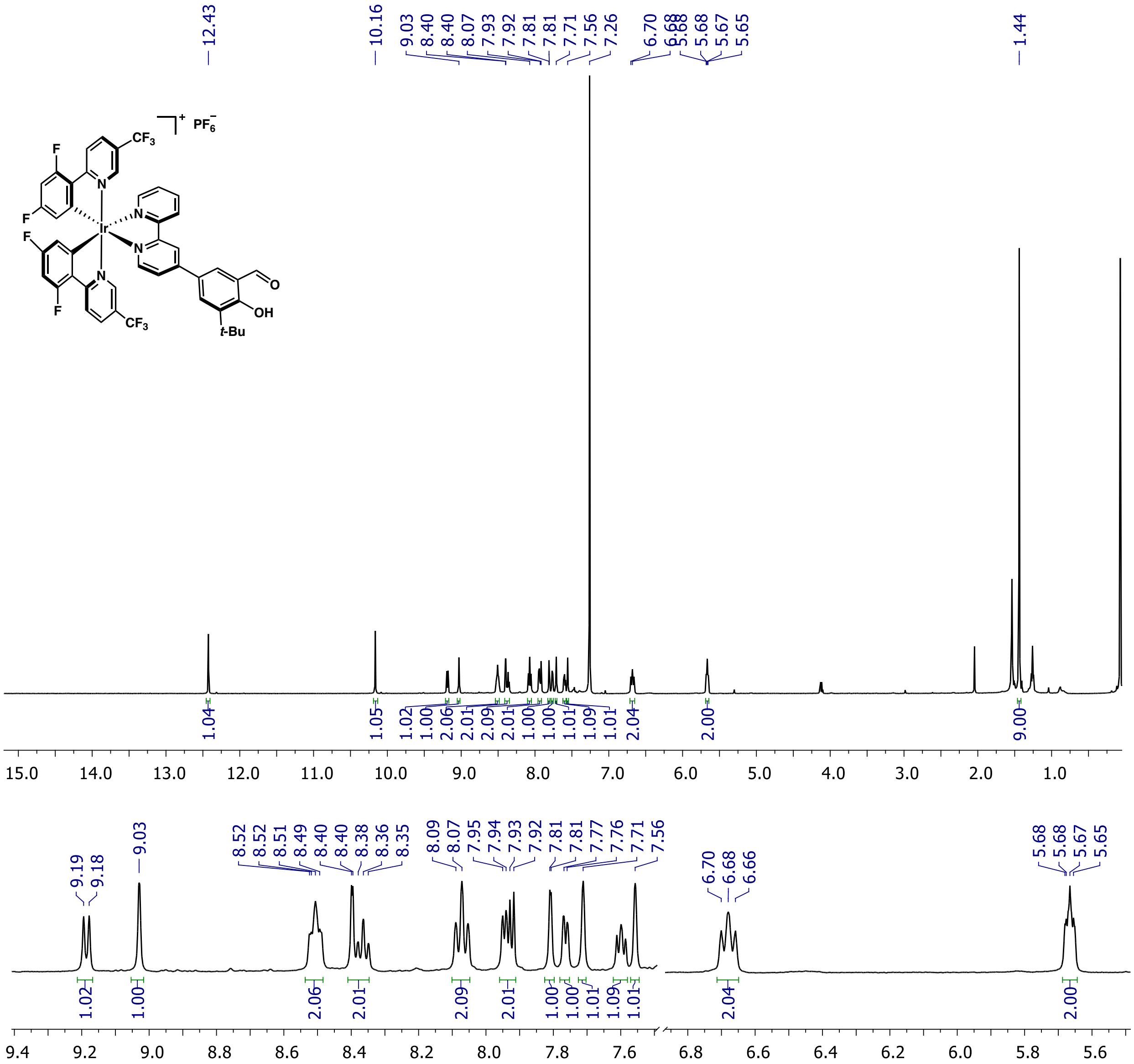

Figure S11. (top) ^{1}H NMR (500 MHz, $CDCl_3$) spectrum of **12** (**Ir-CHO**) in $CDCl_3$, (bottom) expansion of selected regions.

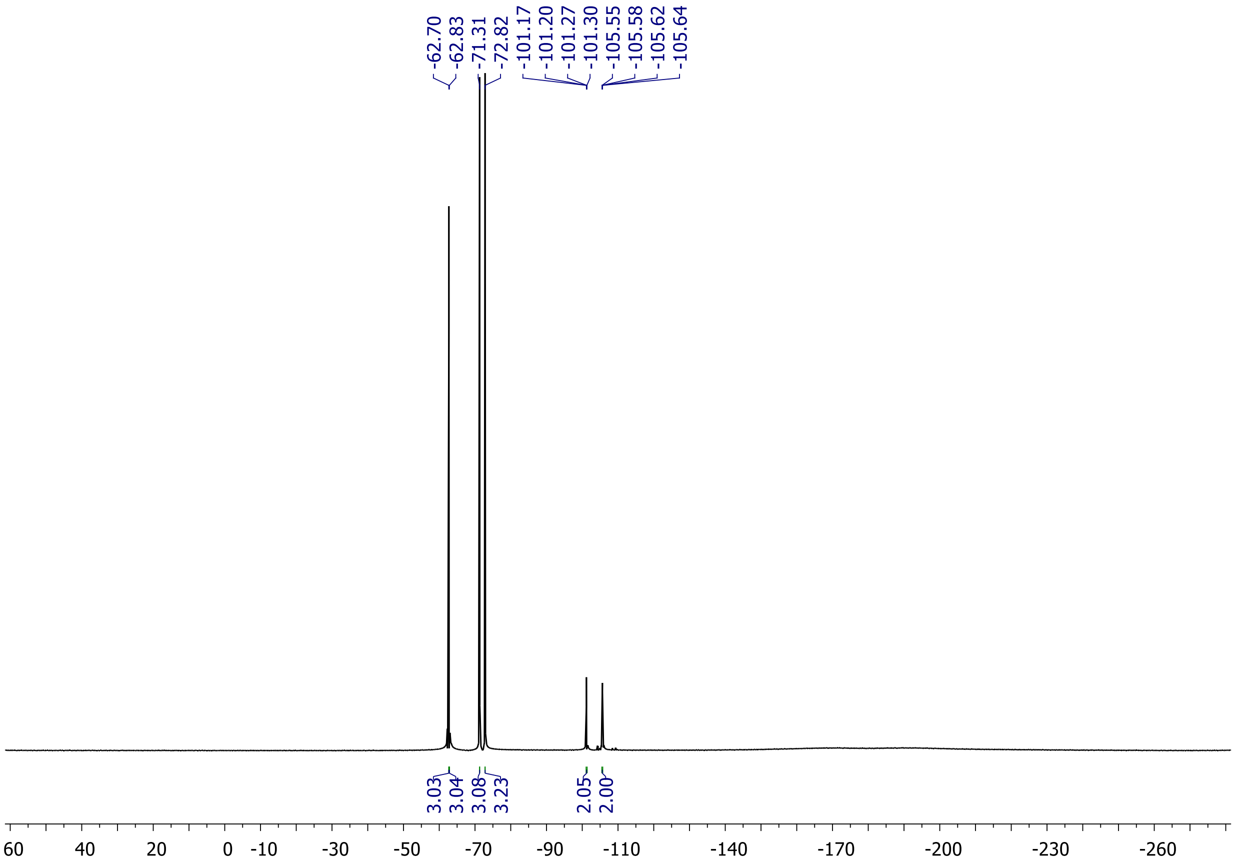

Figure S12. 500 MHz ^{19}F NMR spectrum of **12** (**Ir-CHO**) in $CDCl_3$, (insets) expansion of selected regions.

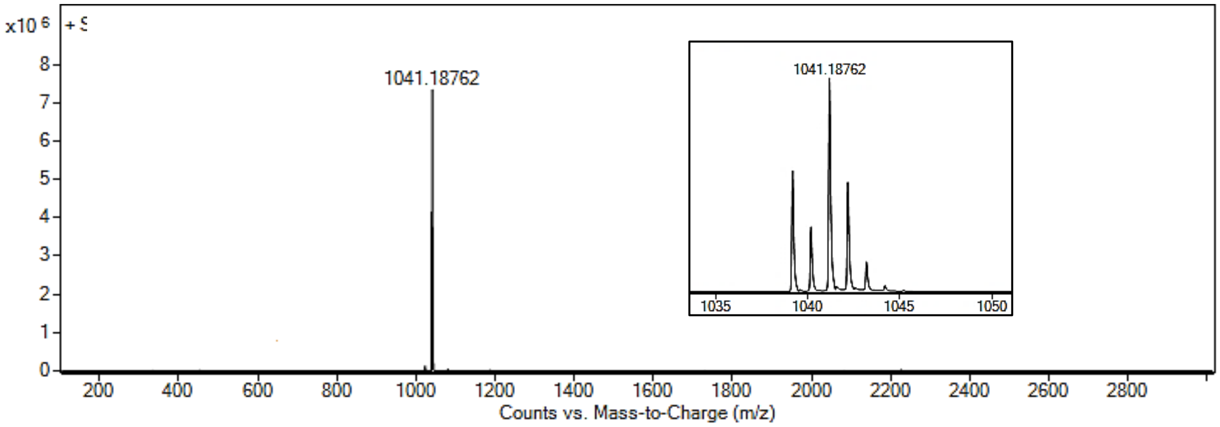

Figure S13. High-resolution mass spectrum of **12** (**Ir-CHO**).

Compound **14** (**B-BTD**)

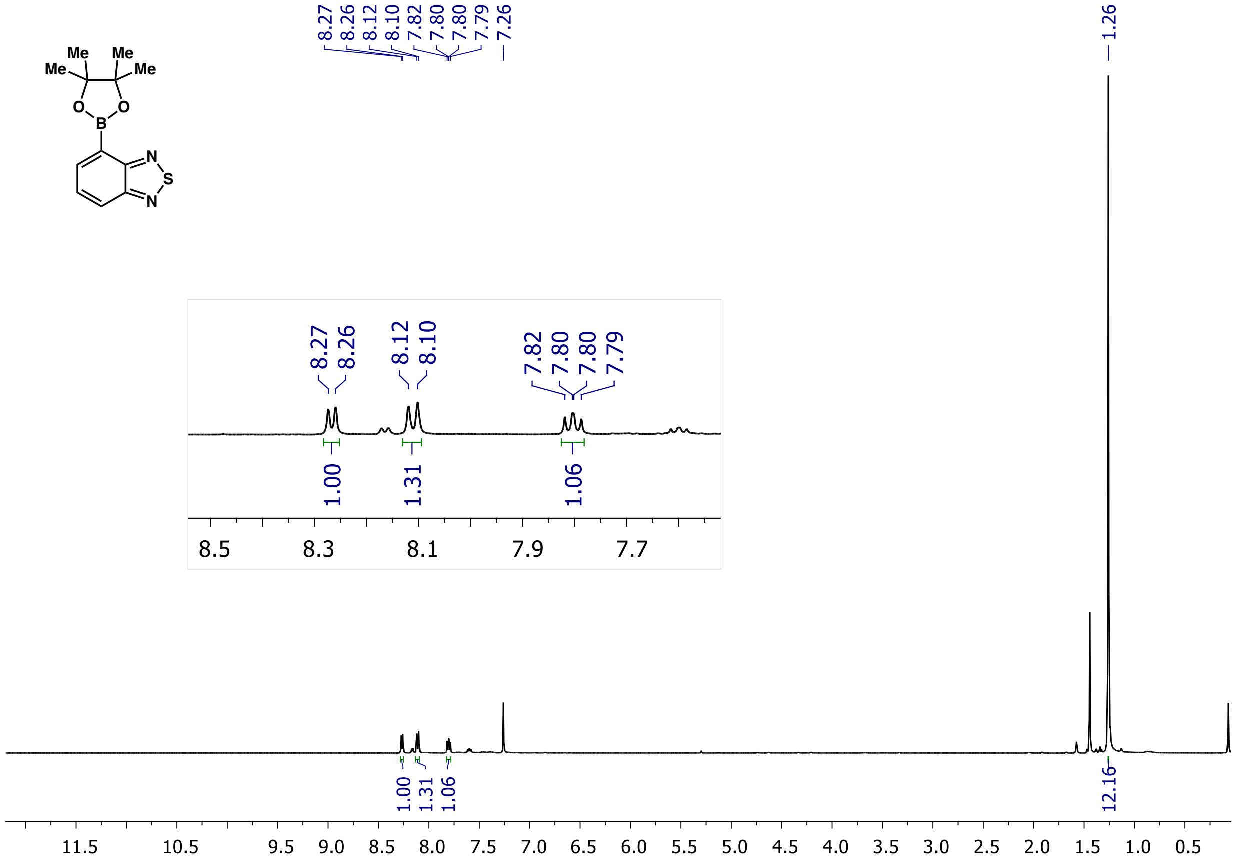

Figure S14. 500 MHz ^{1}H NMR spectrum of **14** (**B-BTD**) in $CDCl_3$, (insets) expansion of selected regions.

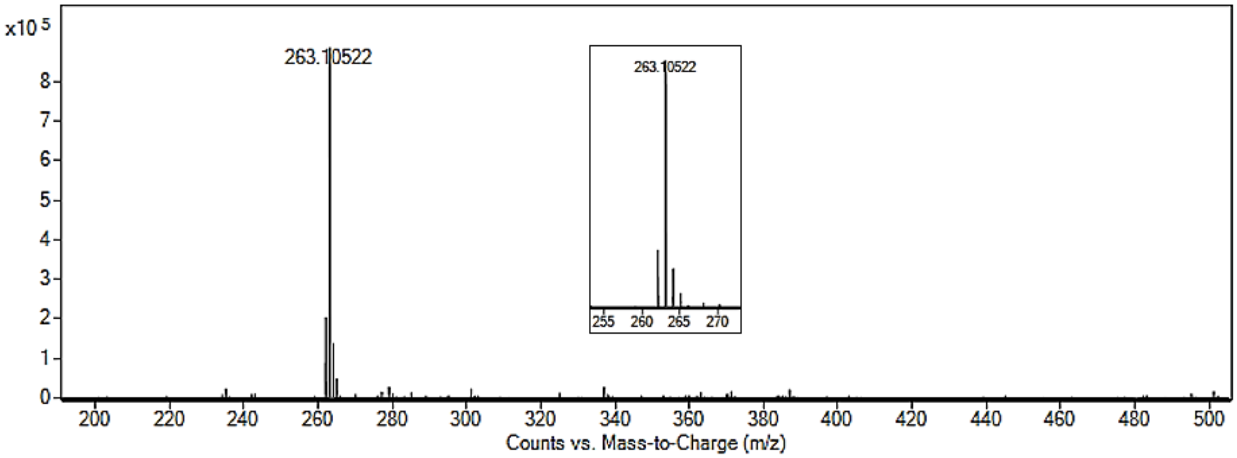

Figure S15. High-resolution mass spectrum of **14** (**B-BTD**).

Compound **15a**

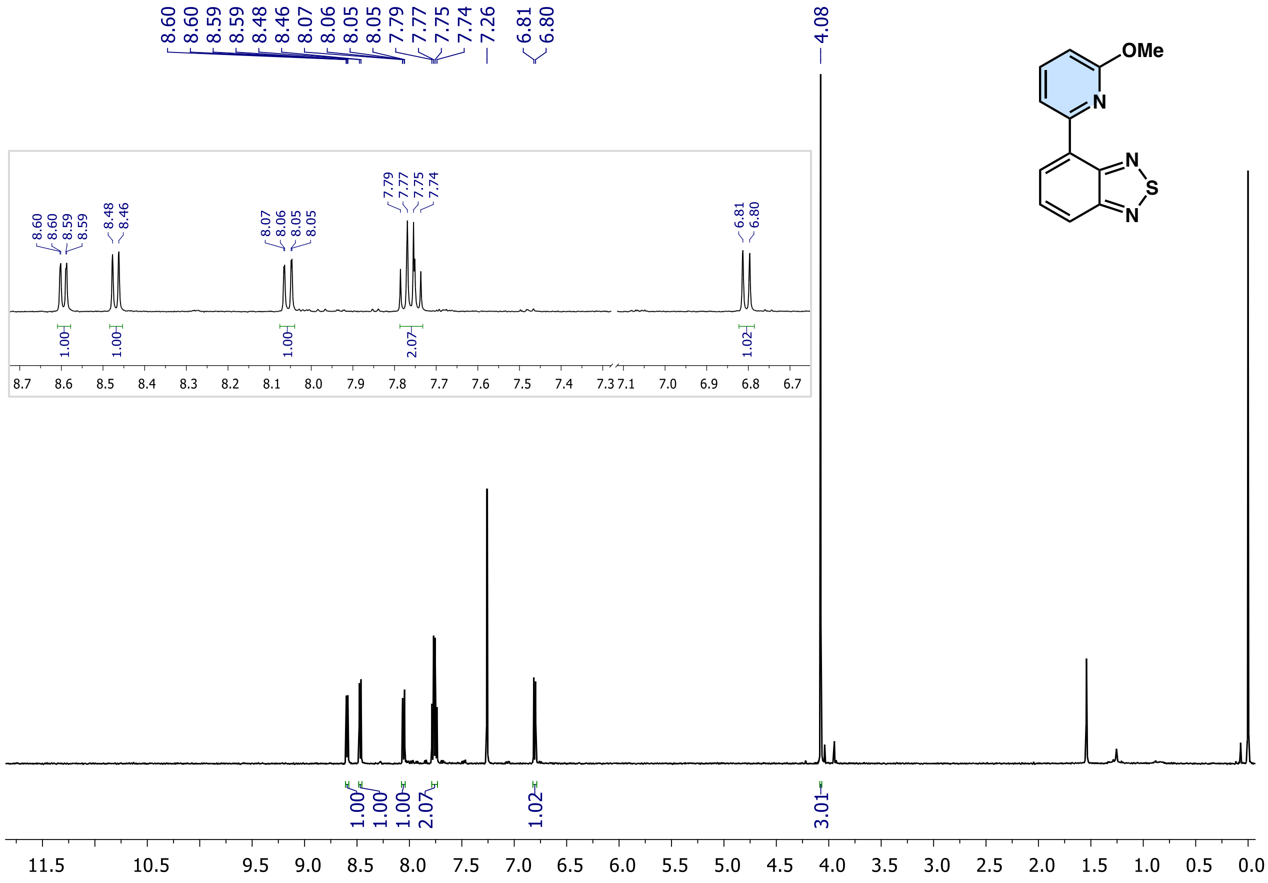

Figure S16. 500 MHz ^{1}H NMR spectrum of **15a** in $CDCl_3$, (insets) expansion of selected regions.

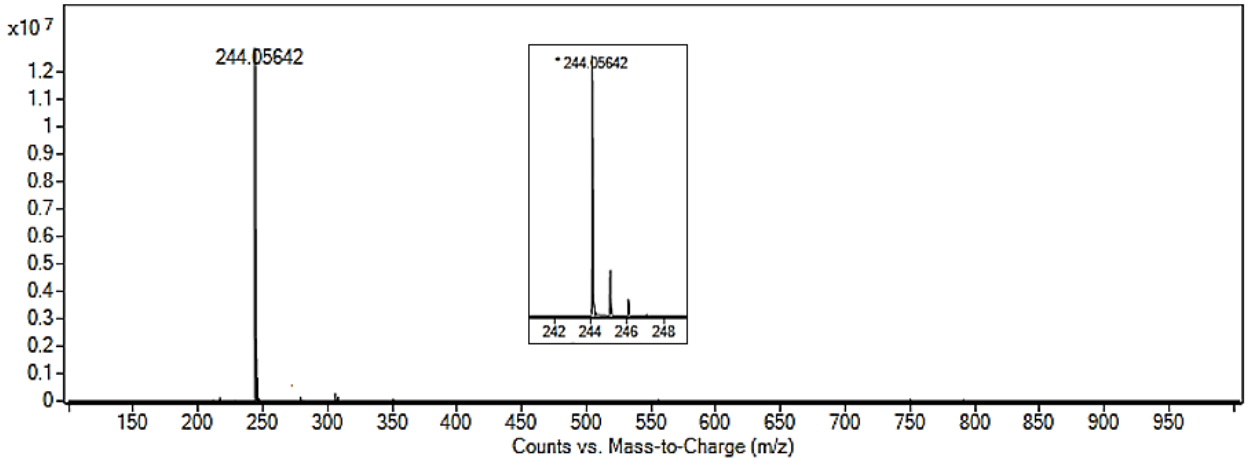

Figure S17. High-resolution mass spectrum of **15a**.

Compound **15b**

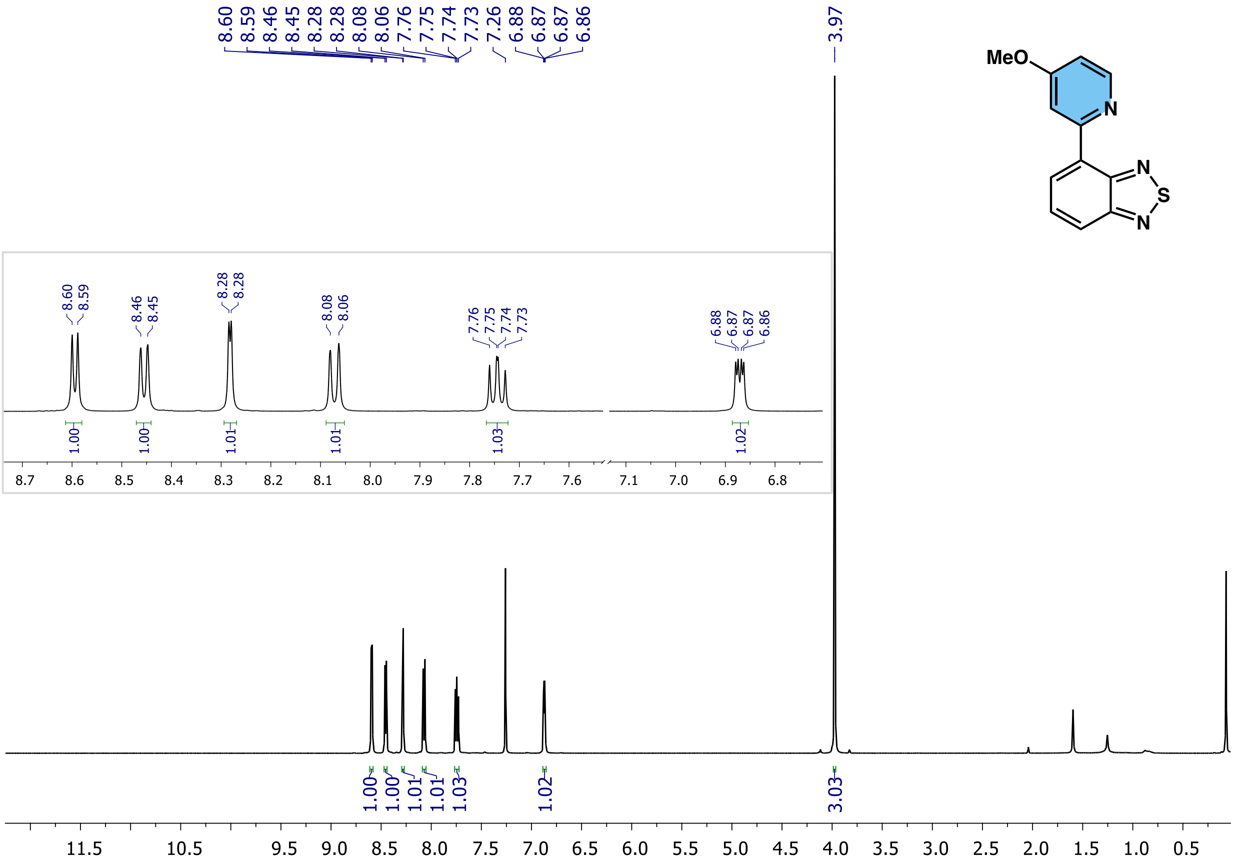

Figure S18. 500 MHz ^{1}H NMR spectrum of **15b** in $CDCl_3$, (insets) expansion of selected regions.

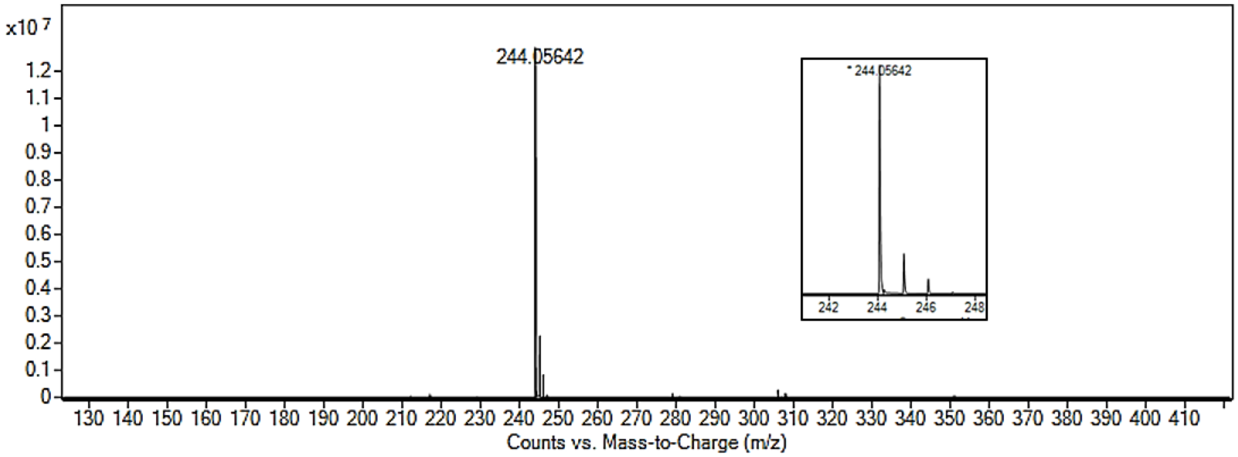

Figure S19. High-resolution mass spectrum of **15b**.

Compound **15c**

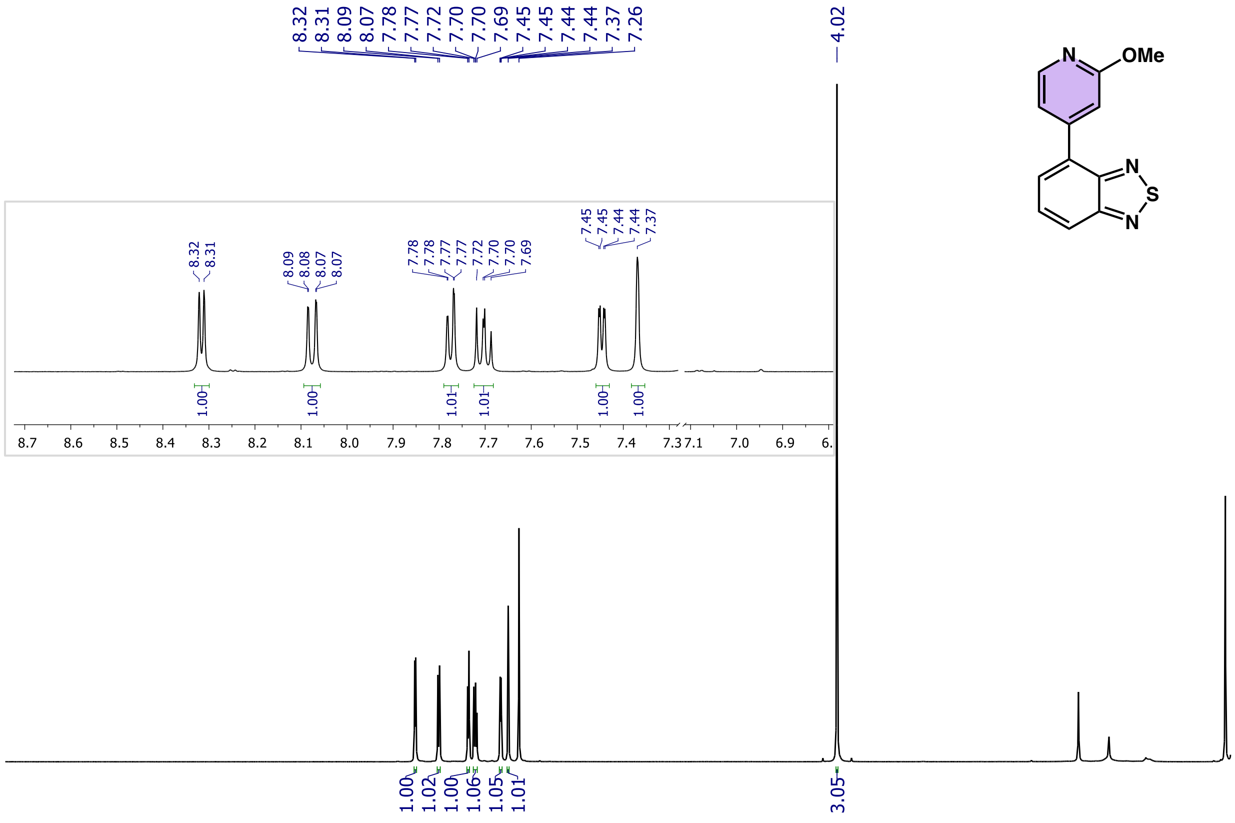

Figure S20. 500 MHz ^{1}H NMR spectrum of **15c** in $CDCl_3$, (insets) expansion of selected regions.

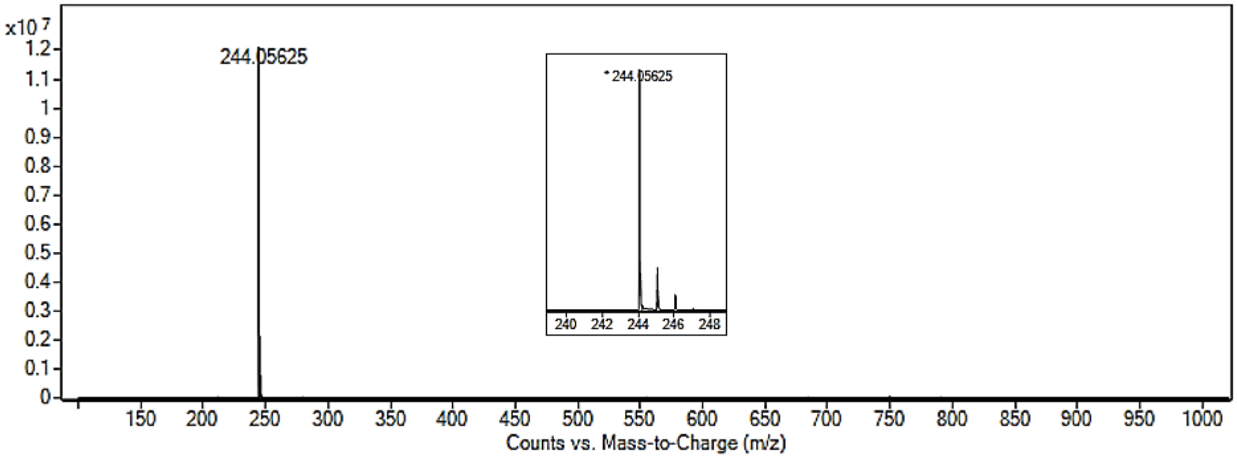

Figure S21. High-resolution mass spectrum of **15c**.

Compound **3**

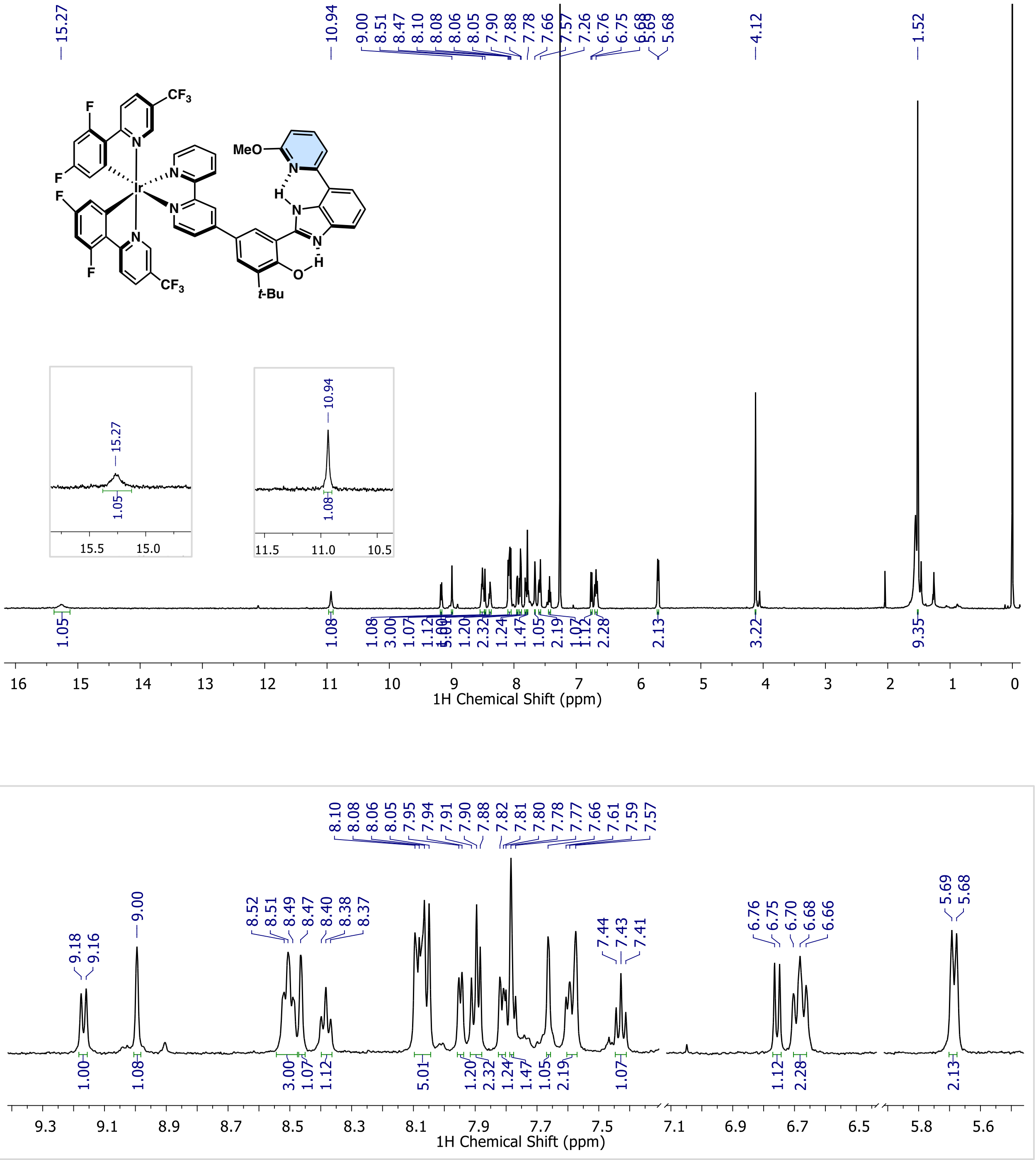

Figure S22. (top) ^{1}H NMR (500 MHz, $CDCl_3$) full spectrum of **3** and (bottom) expansion of selected regions.

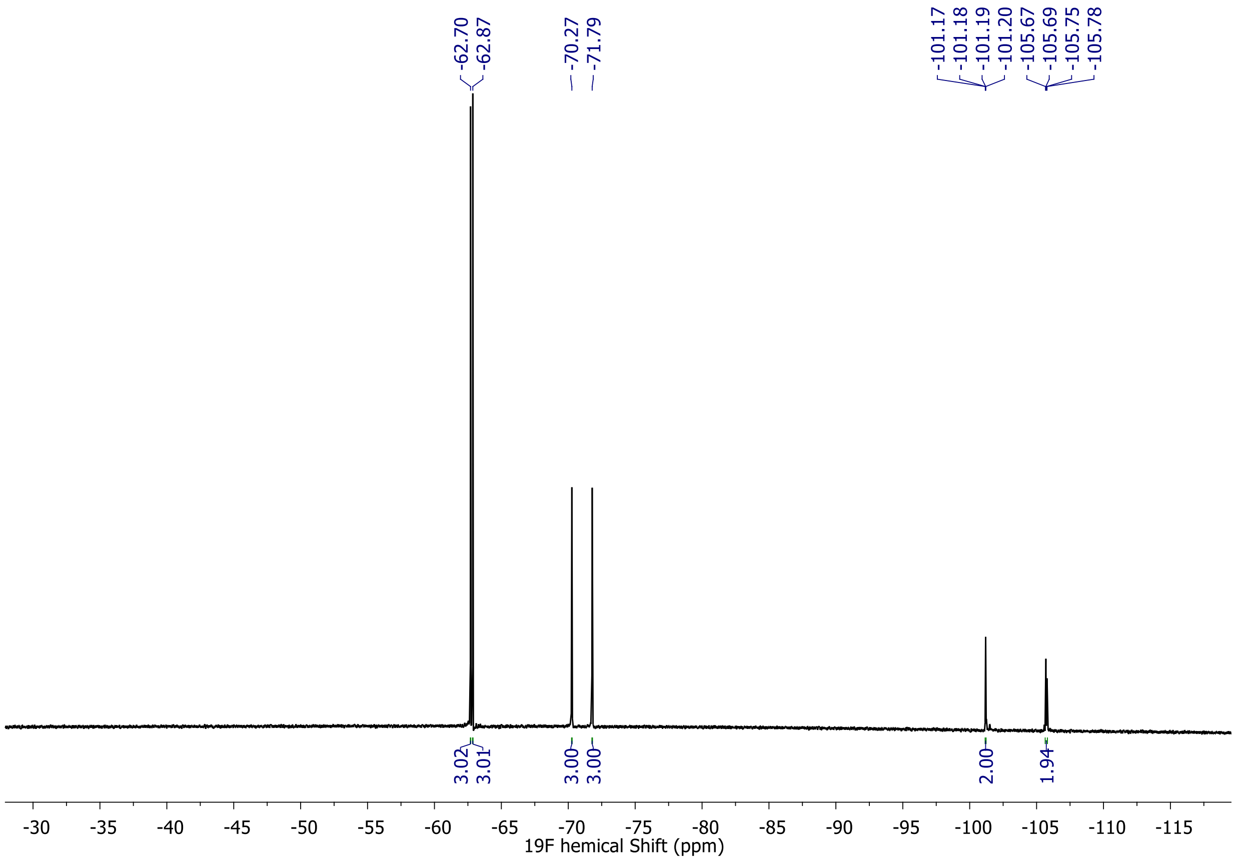

Figure S23. 500 MHz ^{19}F NMR spectrum of **3** in $CDCl_3$.

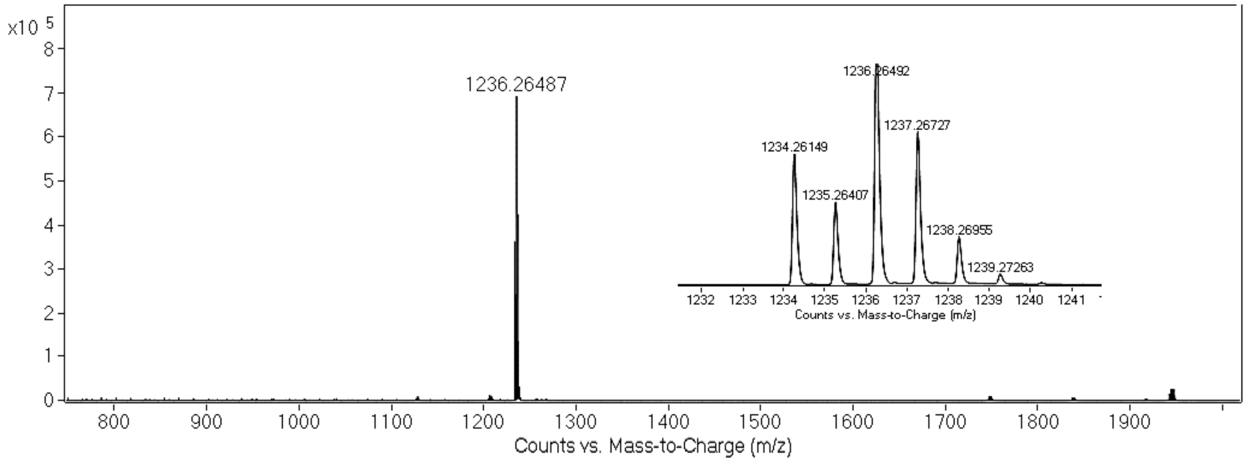

Figure S24. High-resolution mass spectrum of **3**.

Compound **4**

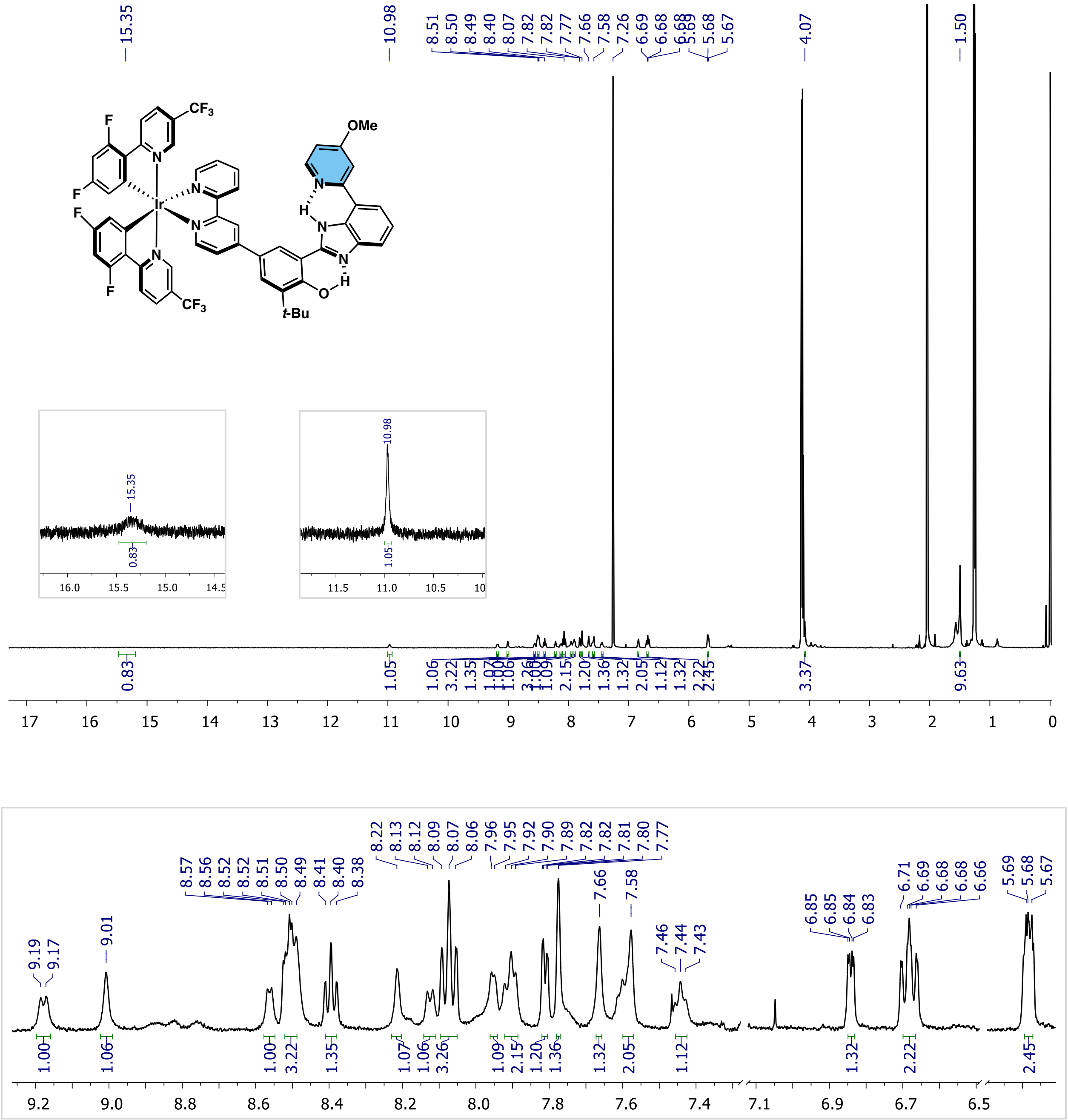

Figure S25. (top) ^{1}H NMR (500 MHz, $CDCl_3$) full spectrum of **4** and (bottom) expansion of selected regions.

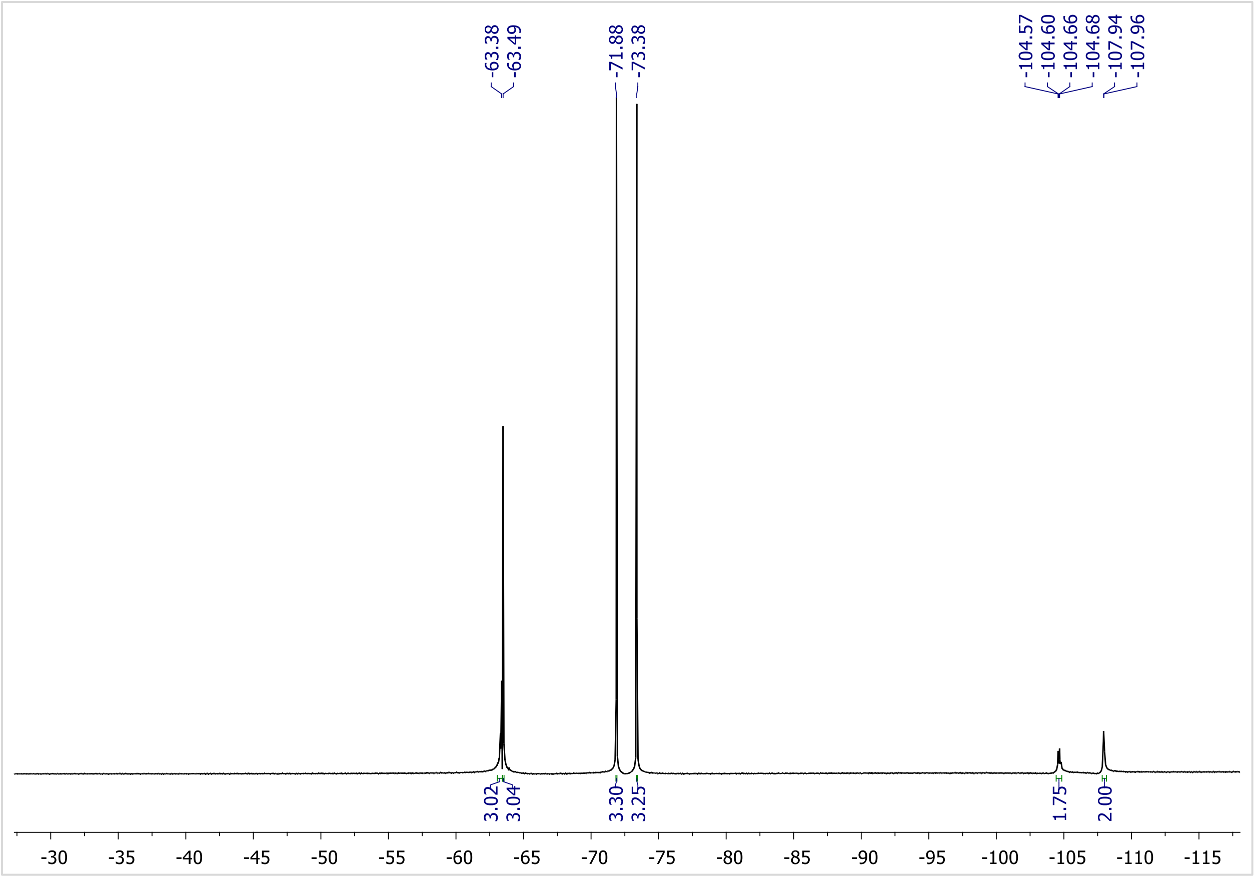

Figure S26. 500 MHz ^{19}F NMR spectrum of **4** in $CDCl_3$.

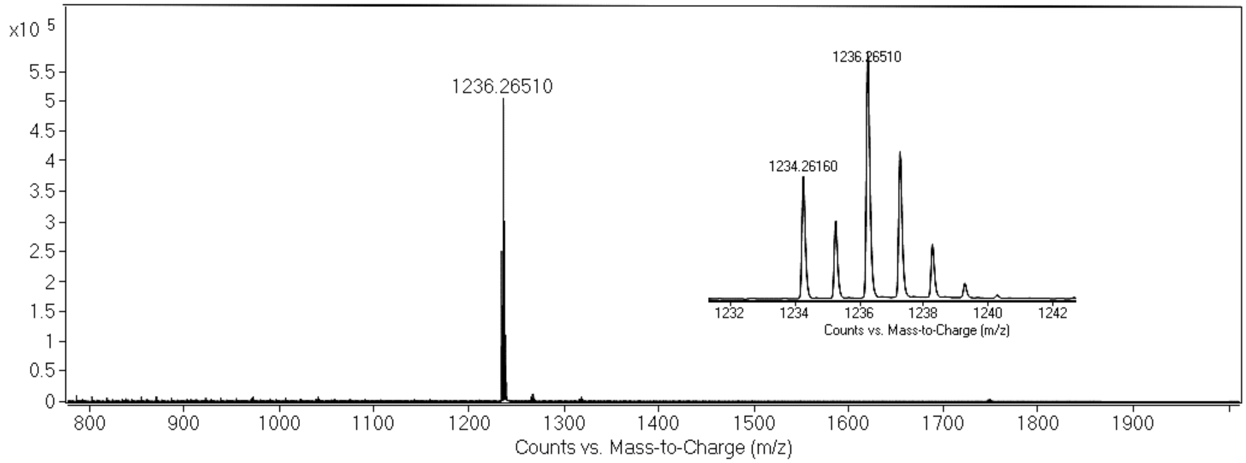

Figure S27. High-resolution mass spectrum of **4**.

Compound **5**

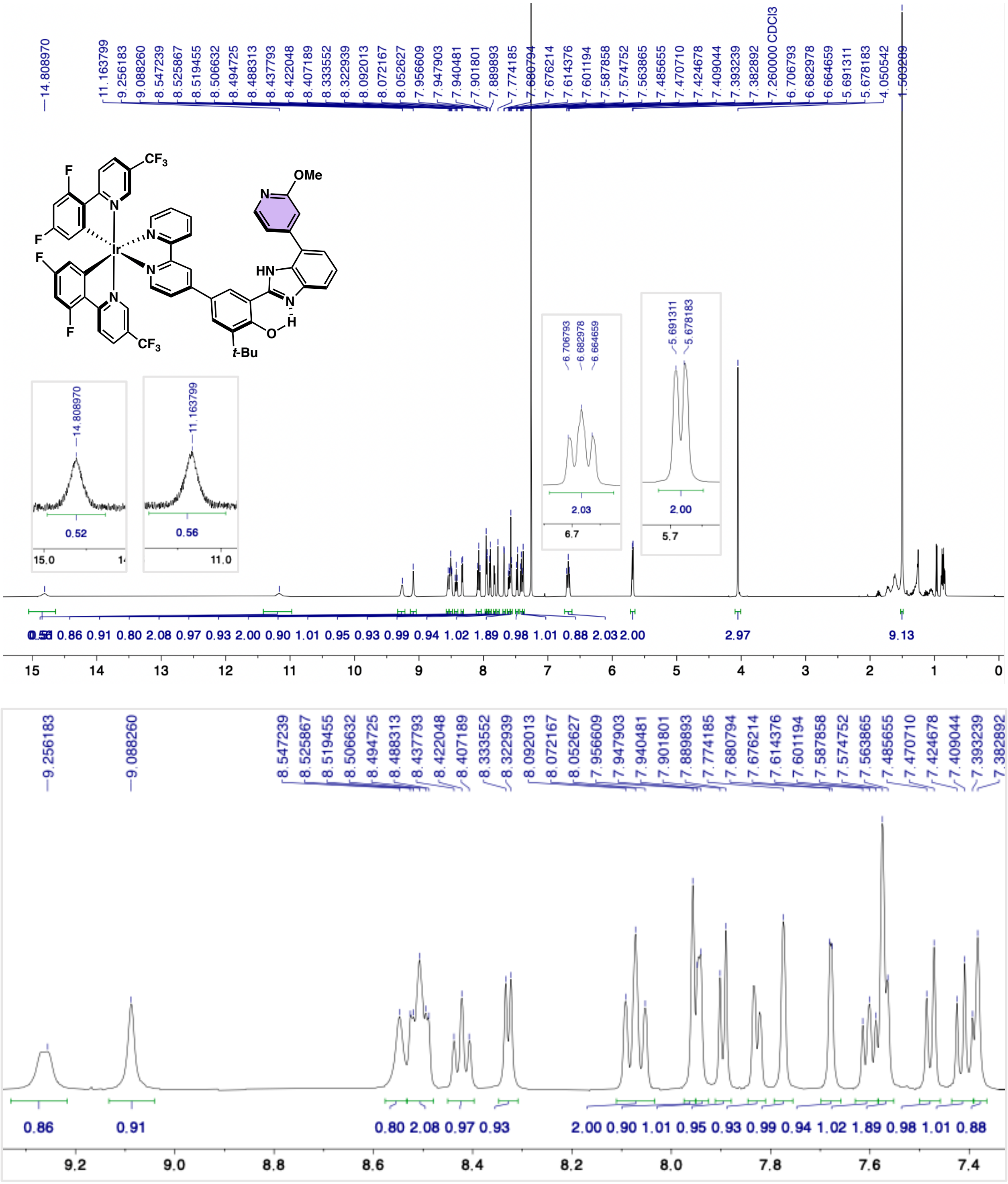

Figure S28. (top) ^{1}H NMR (500 MHz, $CDCl_3$) spectrum of **5** and (bottom) expansion of selected regions.

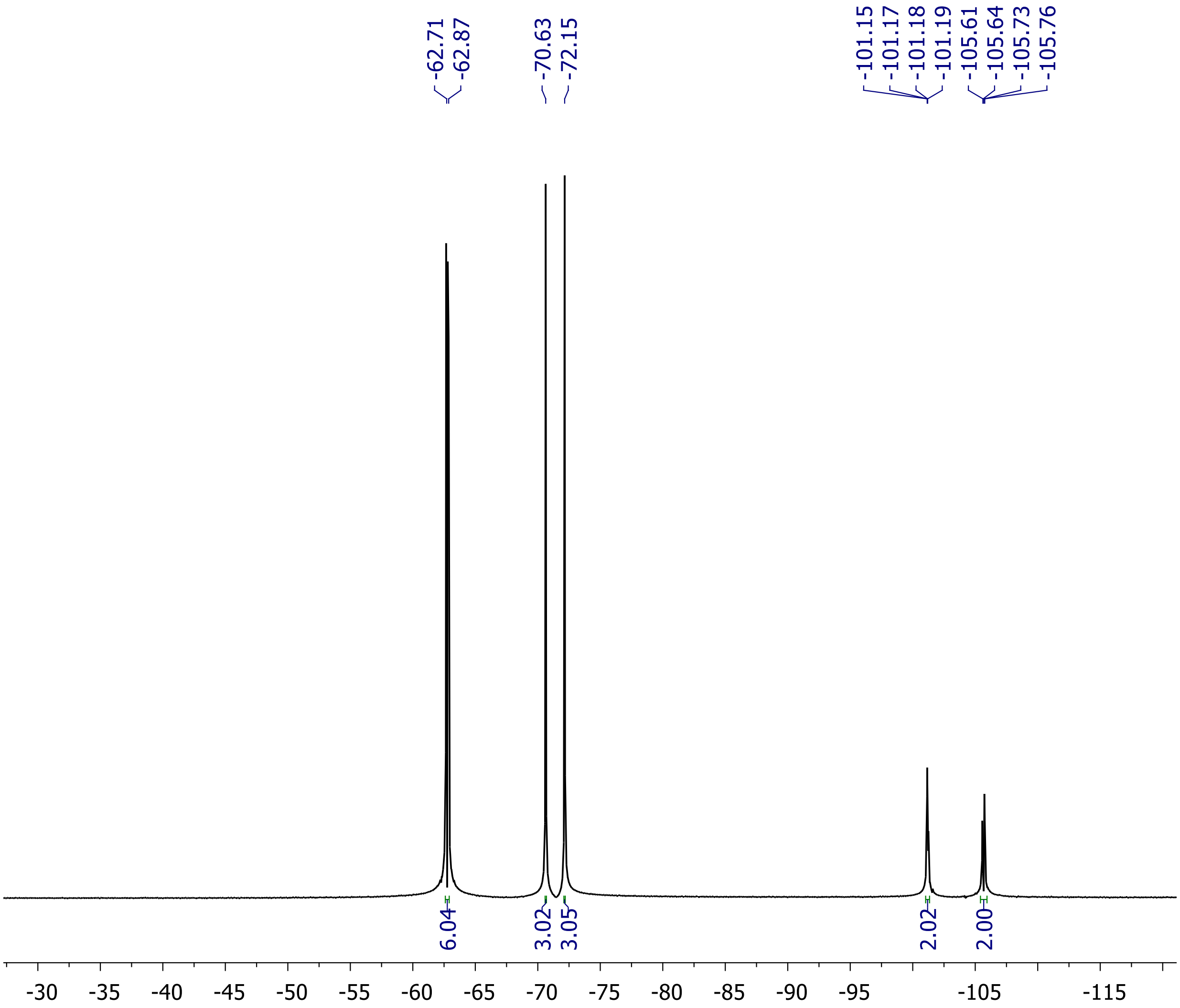

Figure S29. 500 MHz ^{19}F NMR spectrum of **5** in $CDCl_3$.

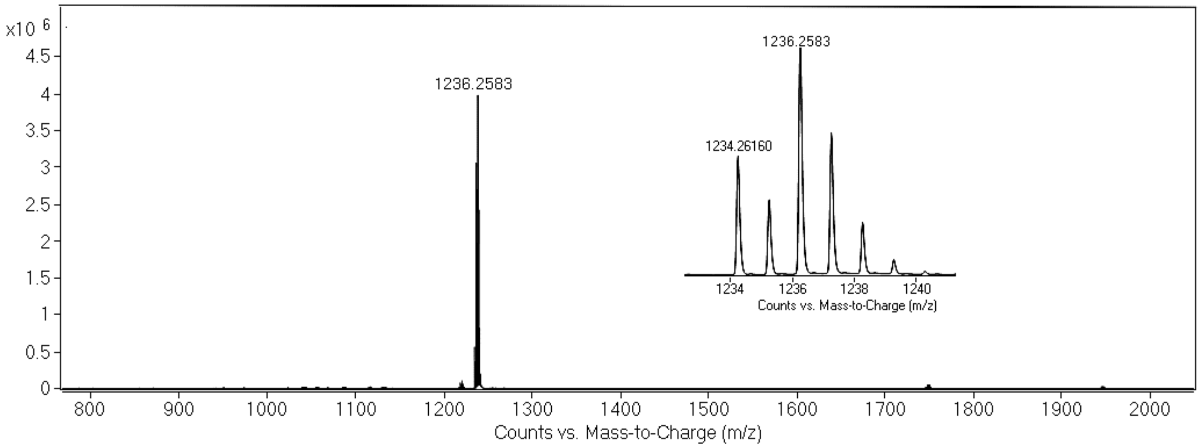

Figure S30. High-resolution mass spectrum of **5**.

1.4. Steady-State Absorption Spectroscopy

Steady-state absorption spectra of photocatalysts **3-5** and their references (**1** and **2**) were recorded on a Shimadzu UV-2550 spectrophotometer using a 1 cm quartz cuvette in anhydrous THF as a solvent. To determine molar extinction coefficients, stock solutions of compounds **1-5** were prepared in THF at a concentration of 0.2 mg/mL. Subsequent dilutions to concentrations ranging from 1 to 10 μM were performed to obtain sample spectra for analysis.

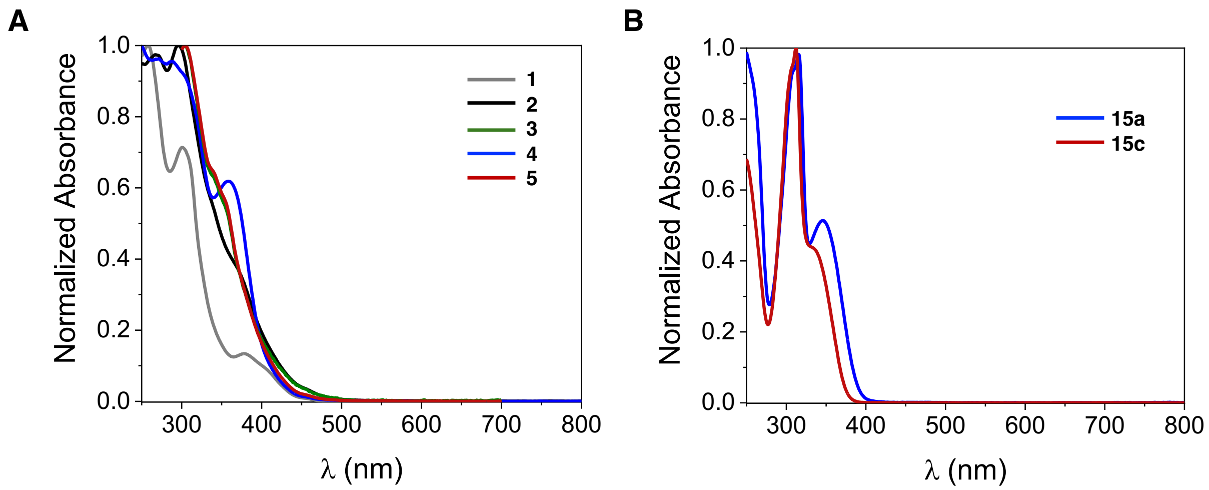

Figure S31. A) Absorption spectra of iridium complexes **1-5** in anhydrous THF. **B)** Normalized absorption spectra of **15a** and **15c** (**Py-BTD**) intermediates showing distinctive electronic transitions at 360 and 350 nm, respectively.

1.5. Steady-State Emission Spectroscopy

Steady-state fluorescence spectra of photocatalysts **3-5** were recorded on a Photon Technology International (PTI) fluorometer equipped with an LPS-220B Lamp Power Supply, a 75 W xenon arc lamp, and a single grating monochromator, providing excitation at 400 nm. Emission spectra were collected at a 90-degree angle to the excitation beam using an 820 Photomultiplier Detection System and an R928 photomultiplier tube operating in single-photon counting mode. Samples were prepared in anhydrous MeCN and THF using 1 cm quartz cuvettes and excited at 400 nm and photocatalysts **3-5** were absorbance-matched with **1** and **2** at the excitation wavelength.

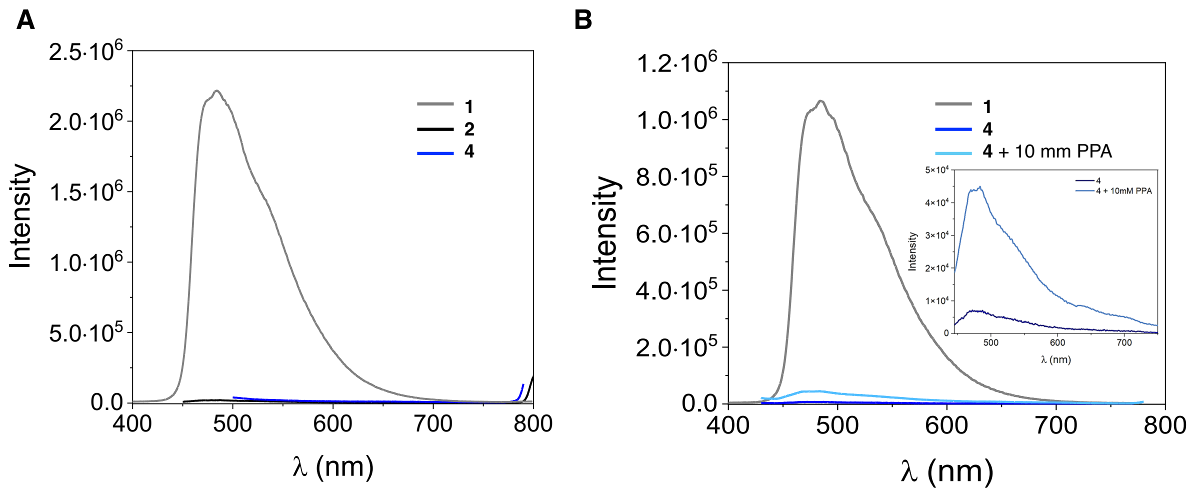

Figure S32. A) Steady-state emission spectra of photocatalysts **4** and **2** relative to **1** in MeCN at room temperature, $\lambda^{irr} = 400$ nm. **B)** Steady-state emission spectra of photocatalyst **4** compared to reference compound **1** in MeCN at room temperature, $\lambda^{ex} = 400$ nm. Complexes **3** and **5** exhibited similar emission quenching behavior. **Inset:** Expanded spectra of **4** upon addition of 10 mM phenylphosphoric acid.

1.6. Electrochemistry Data

Cyclic voltammetry measurements were conducted using a Biologic potentiostat with a three-electrode cell configuration. A glassy carbon working electrode (WE) of 3 mm diameter, a platinum wire counter electrode (CE), and a silver wire pseudo-reference electrode (RE) were employed. All cyclic voltammograms (CV) were collected in anhydrous acetonitrile, purified by distillation, and kept over molecular sieves and K_2CO_3, containing 1 mM of the target compound. Experiments were conducted at room temperature under an argon atmosphere, with tetrabutylammonium hexafluorophosphate (nBu_4NPF_6, 0.1 M in MeCN) as the supporting electrolyte and the scan rate of 1000 mV/s. The glassy carbon working electrode was prepared for each measurement by polishing with an aluminum slurry on a microcloth pad, followed by rinsing with solvent and drying with nitrogen. The potential of the pseudo-reference electrode was calibrated against the ferrocenium/ferrocene redox couple as an internal standard and adjusted to the saturated calomel electrode (SCE) scale ($E_{1/2}$ taken to be 0.40 V vs SCE). [7]

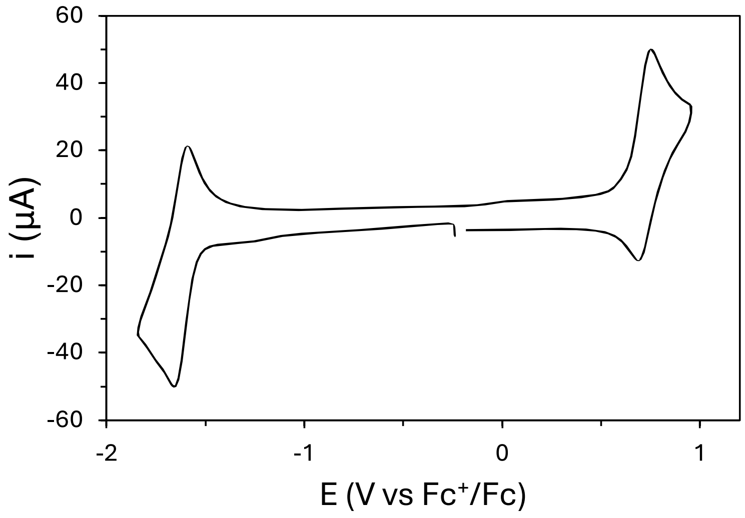

Figure S33. Cyclic voltammogram of **2**. Experimental conditions: 1 mM of the **2**, 0.1 M nBu_4NPF_6 supporting electrolyte in dry MeCN. WE: glassy carbon. Pseudo RE: Ag wire (ferrocene as internal reference). CE: Pt wire. Scan rate, 1000 mV s^{-1}.

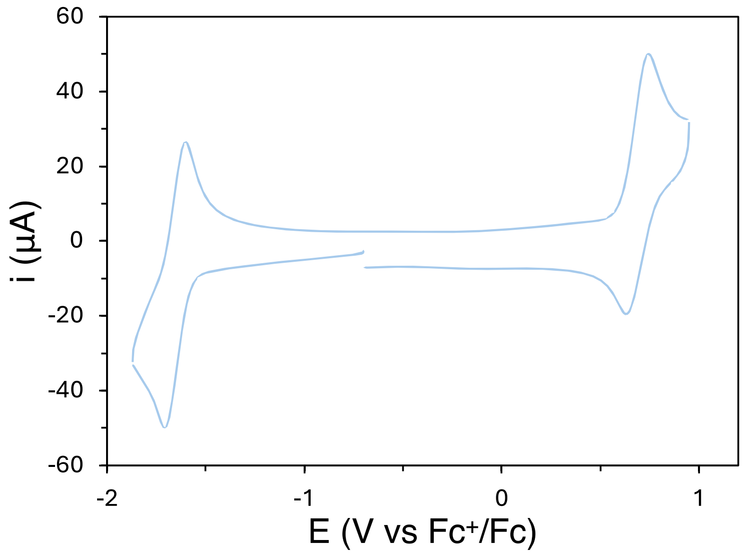

Figure S34. Cyclic voltammogram of **3**. Experimental conditions: 1 mM of the **3**, 0.1 M nBu_4NPF_6 supporting electrolyte in dry MeCN. WE: glassy carbon. Pseudo RE: Ag wire (ferrocene as internal reference). CE: Pt wire. Scan rate, 1000 mV s^{-1}.

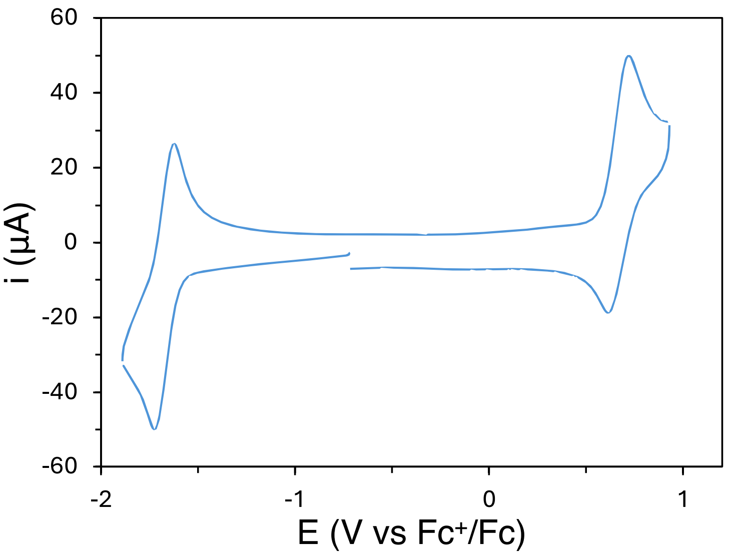

Figure S35. Cyclic voltammogram of **4**. Experimental conditions: 1 mM of the **4**, 0.1 M nBu_4NPF_6 supporting electrolyte in dry MeCN. WE: glassy carbon. Pseudo RE: Ag wire (ferrocene as internal reference). CE: Pt wire. Scan rate, 1000 mV s^{-1}.

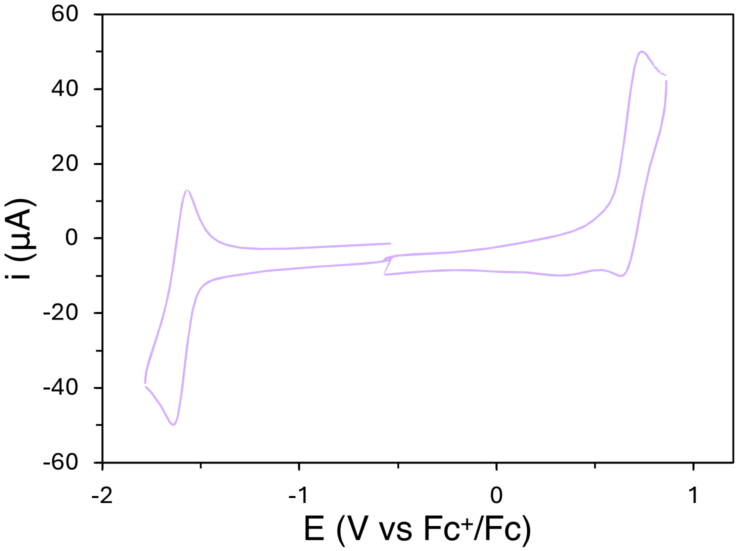

Figure S36. Cyclic voltammogram of **5**. Experimental conditions: 1 mM of the **5**, 0.1 M nBu_4NPF_6 supporting electrolyte in dry MeCN. WE: glassy carbon. Pseudo RE: Ag wire (ferrocene as internal reference). CE: Pt wire. Scan rate, 1000 mV s^{-1}.

1.7. Infrared Spectroelectrochemistry

IRSEC measurements were carried out using a Bruker Vertex 70 spectrometer equipped with a GloBar MIR source, broadband KBr beamsplitter, and a liquid nitrogen cooled MCT detector. Spectra were collected in absorption mode under a dry nitrogen purge with 2 cm^{-1} resolution. Each spectrum represented an average of 64 scans of the photocatalyst (8 mM) in anhydrous deuterated MeCN containing 0.1 M nBu_4NPF_6 as supporting electrolyte. A Biologic potentiostat was coupled to an optically transparent thin-layer electrochemical cell (Spectroelectrochemistry Reading RT OTTLE cell) equipped with NaCl windows (path length 0.2 mm). The cell contained a Pt mesh counter electrode, an Ag wire pseudo-reference electrode, and a Pt mesh working electrode strategically placed in the light path of the IR spectrophotometer. A detailed description of the spectroelectrochemical technique can be found in the literature. [7,8]

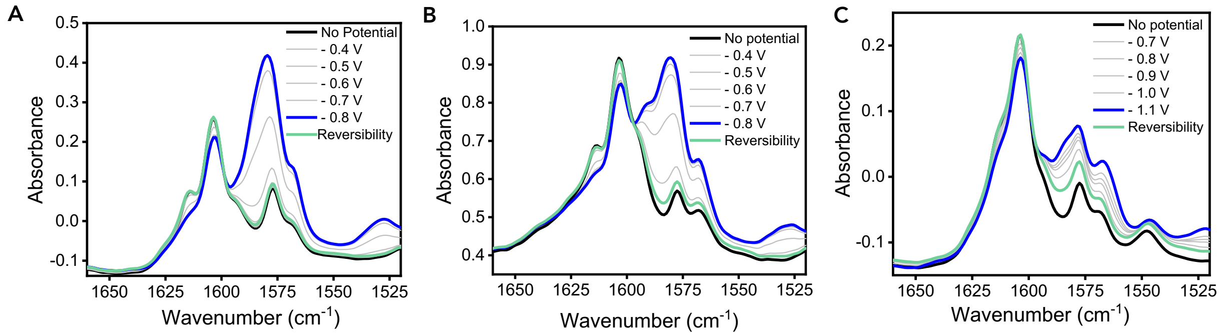

Figure S37. Panels **A**, **B**, and **C**: Reduction species of photocatalysts **3-5** (1650-1520 cm^{-1}). Black line: resting potential (no polarization); blue line: reduced species; green line: reversibility; gray lines: spectral evolution during polarization.

1.8. Visible Spectroelectrochemistry

Visible spectroelectrochemical measurements were performed using a Biologic SP-200 potentiostat coupled with a Pine Research platinum honeycomb spectroelectrochemical electrode and a Shimadzu SolidSpec-3700 Vis-NIR spectrometer. A silver wire served as the pseudo-reference electrode. Experiments were conducted in 0.1 M nBu_4PF_6 in CD_3CN. Reported redox potentials were referenced to the silver wire.

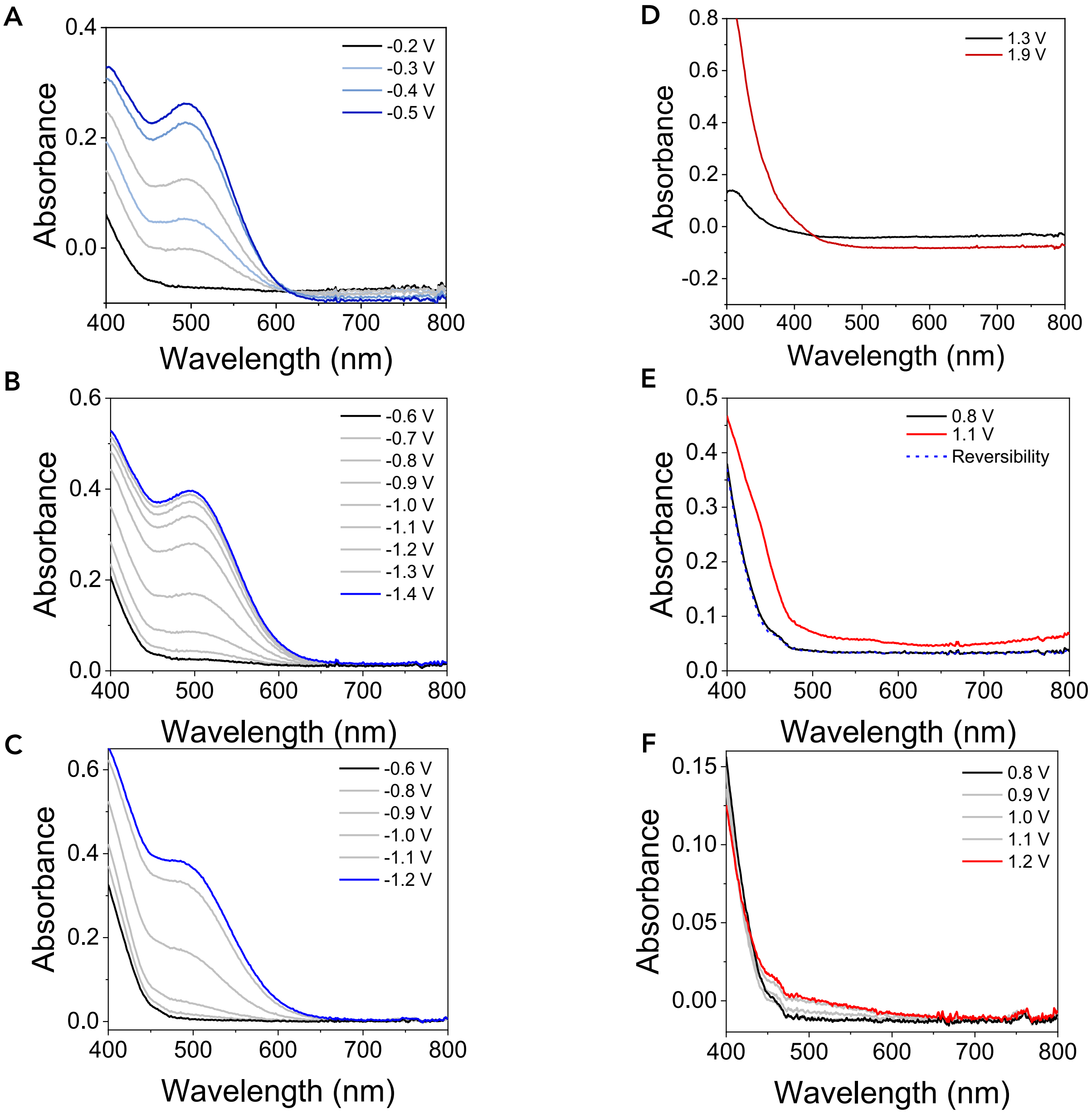

Figure S38. VIS-SEC spectra of photocatalysts **Ir-BIP-Py 3-5** (0.6 mM) in CD_3CN solution containing 0.1 M nBu_4PF_6 as supporting electrolyte. Panels **A-C** display the spectra under reductive conditions (blue lines), while panels **D-F** correspond to oxidative conditions (red lines) for photocatalysts **3**, **4**, and **5**, respectively.

The black line represents the spectrum at resting potential (no polarization) and gray lines depict spectral evolution during polarization.

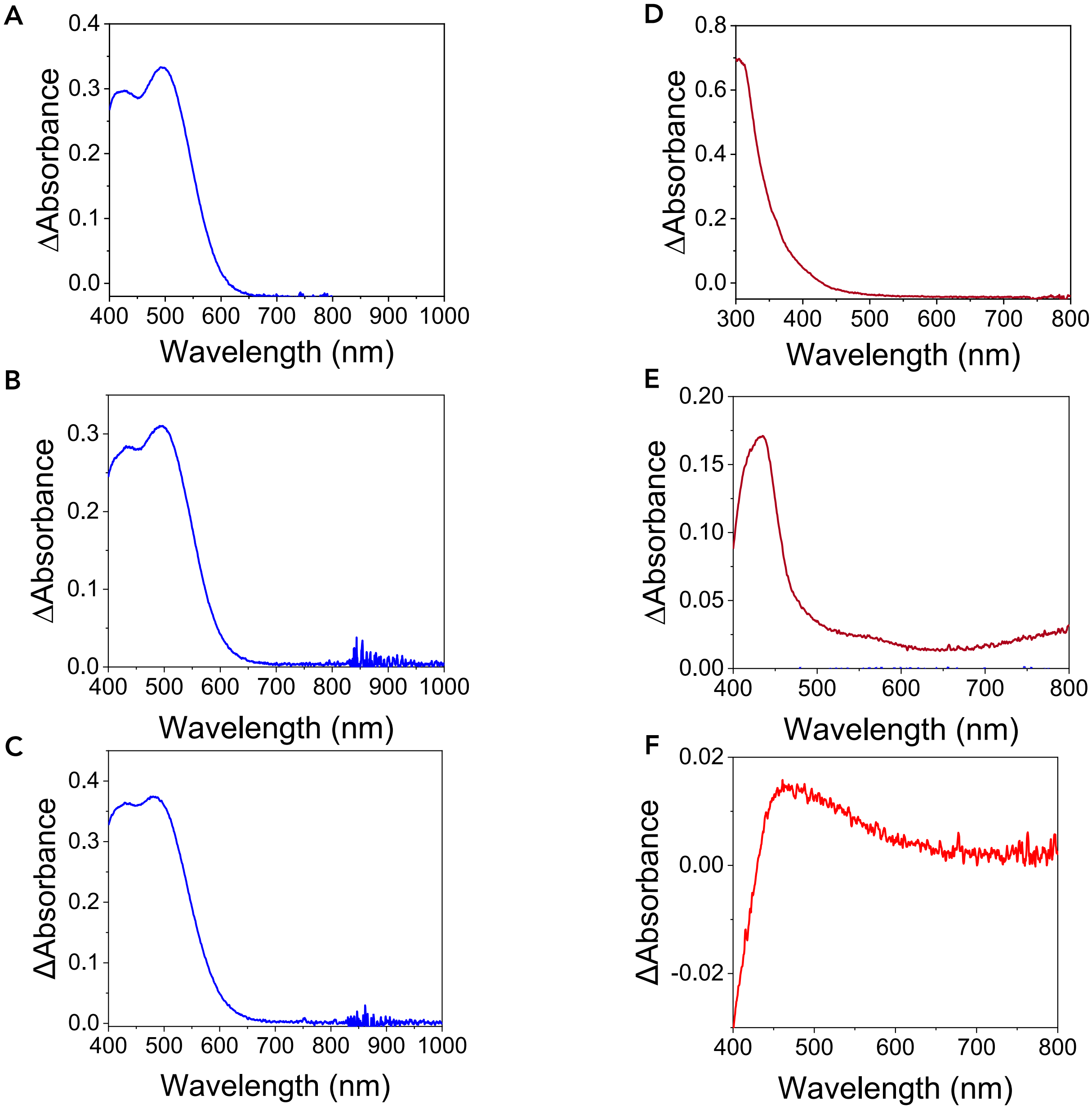

Figure S39. VIS-SEC spectra of photocatalysts **Ir-BIP-Py 3-5** (0.6 mM) in CD_3CN solution containing 0.1 M nBu_4PF_6 as supporting electrolyte. Panels **A-C** display the spectra under reductive conditions (blue lines), while panels **D-F** correspond to oxidative conditions (red lines) for photocatalysts **3**, **4**, and **5**, respectively.

1.9. Ultrafast and Nanosecond Visible Transient Absorption Spectroscopy

Ultrafast transient absorption (TA) spectra were collected using a commercial femtosecond transient absorption spectrometer (Helios Fire, Ultrafast System). The 800 nm fundamental pulses were generated from a Ti: Sapphire laser system (Astrella, Coherent Inc., <100 fs, 7 mJ/pulse, 1 kHz repetition rate) and were split into two beams. One beam was used to generate a white-light (WL) supercontinuum inside Helios Fire as the probe. The second beam was used to generate 440 nm pump pulses via an optical parametric amplifier (OPerA Solo, Coherent Inc.). The pump power was then reduced to 70–20 nJ/pulse using a neutral density filter. The pump pulses were depolarized before focusing on the sample, which was loaded into a quartz cuvette with an optical path length of 1 mm and continuously stirred during the experiment. The instrument response function (IRF) of this TA system was modeled by fitting it to a Gaussian curve, with a full width at half maximum of 150 fs. Samples for nanosecond TA measurements were prepared in a MBraun glovebox and run in a specially designed 2 mm quartz cuvette that is sealed for the duration of the transient absorption study. Each sample is absorbance-matched to an optical density of 0.2 at an excitation wavelength of 440 nm. To minimize sample degradation, the solution was continually stirred and translated during the experiment. Each sample was excited with a 100 nJ pulse centered at 440 nm (Figure S40) generated with Ekspla PT403 tunable picosecond laser and probed using a white-light supercontinuum spectrum generated by focusing 800 nm light from a Coherent Astrella (45 fs, two 2.5 mJ outputs) in a continuously vertically translating calcium fluoride crystal. The electronic delay between the two pulses is measured using a commercial setup from Ultrafast Systems. The lifetimes of the species were obtained by fitting using the Glotaran software package.[9] An evolutionary-associated difference spectra model was employed (EADS) to capture the dynamics and time components.

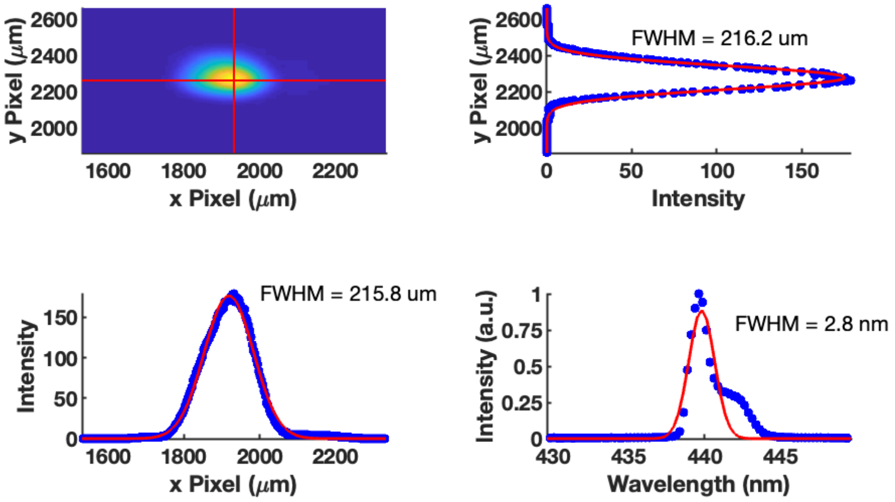

Figure S40. 440 nm Excitation Pulse Characterization

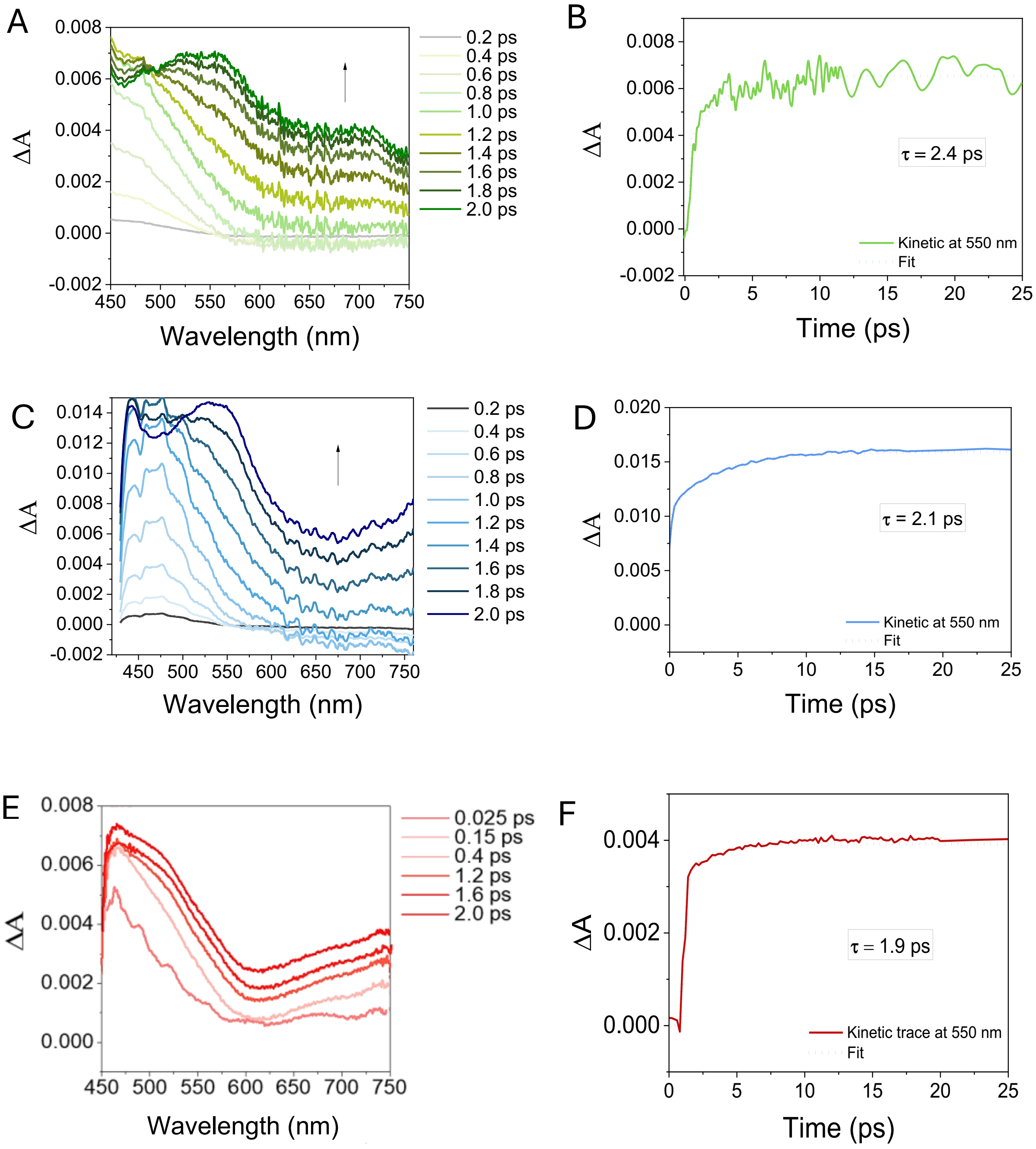

Figure S41. Ultrafast visible transient absorption spectra (fs-TAS) of photocatalysts **Ir-BIP-Py 3-5** (OD_{400nm} = 0.2) in MeCN solution, λ^{irr} = 400 nm. Panels **A**, **C**, and **E** display the time-resolved spectra (TRS), while panels **B**, **D**, and **F** correspond to the kinetic trace of photocatalysts **3**, **4**, and **5**, respectively.

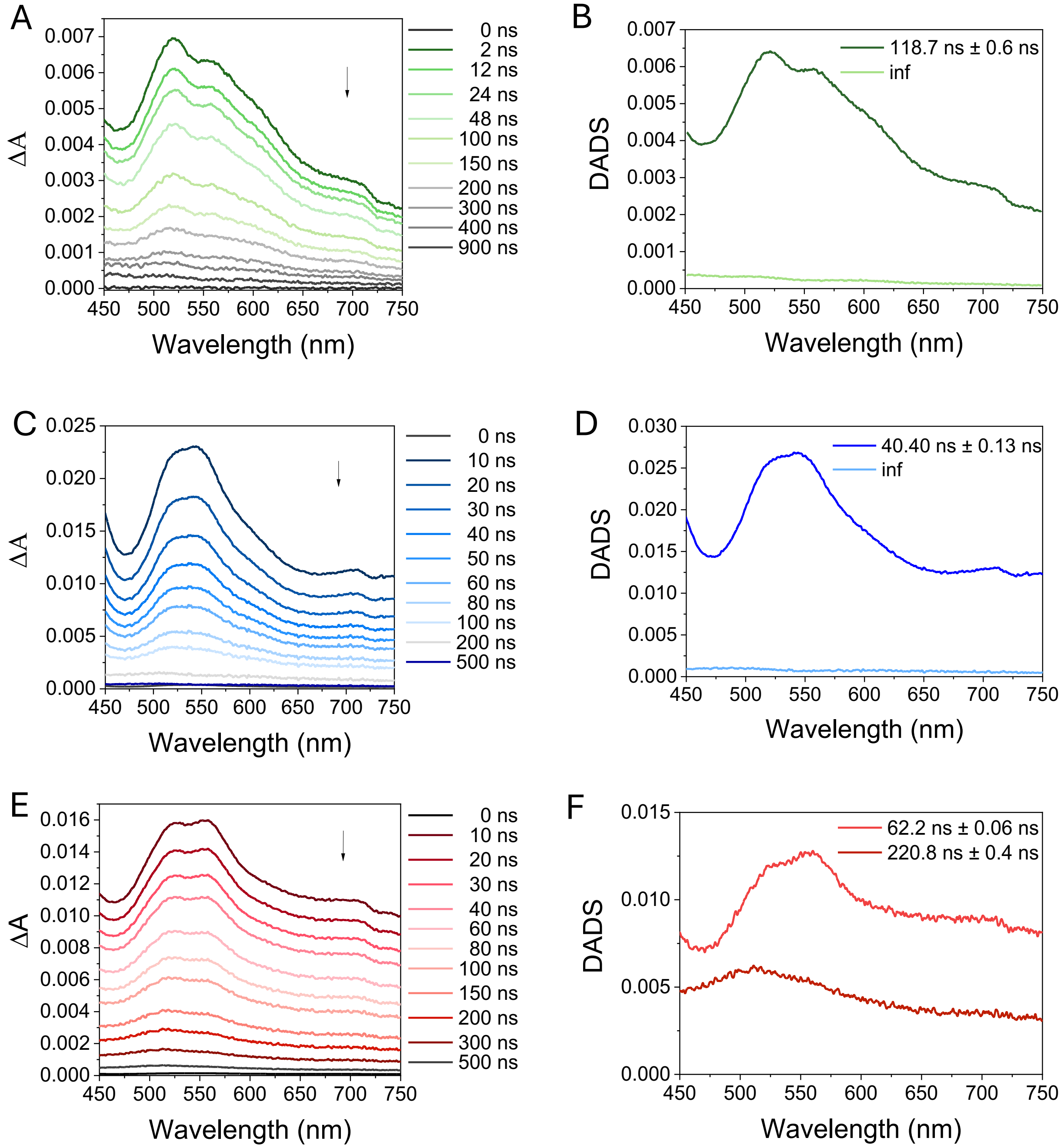

Figure S42. Nanosecond visible transient absorption spectra (ns-TAS) of photocatalysts **Ir-BIP-Py 3-5** (OD_{400nm} = 0.2) in MeCN solution, λ^{irr} = 400 nm. Panels **A**, **B**, and **C** display the time-resolved spectra (TRS), while panels **D**, **E**, and **F** correspond to the kinetic trace of photocatalysts **3**, **4**, and **5**, respectively.

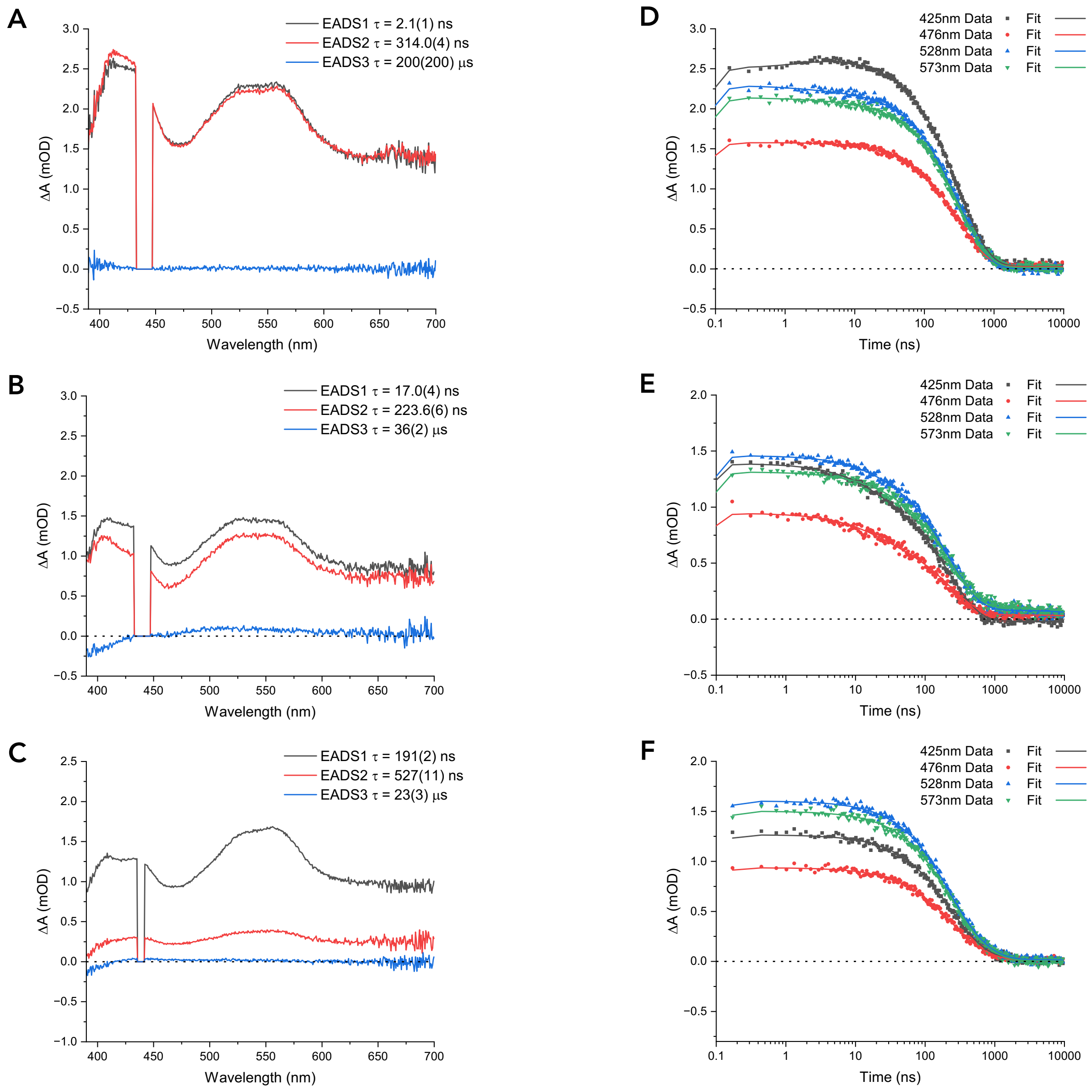

Figure S43. Global analysis of nanosecond visible transient absorption spectra (ns-TAS) of photocatalysts **Ir-BIP-Py 3-5** (OD_{400nm} = 0.2) in THF solution, λ^{irr} = 440 nm. Panels **A**, **B**, and **C** display the evolution-associated difference spectra (EADS), and panels **D**, **E**, and **F** show kinetic traces and global analysis fits at selected wavelengths for photocatalysts **3**, **4**, and **5**, respectively.

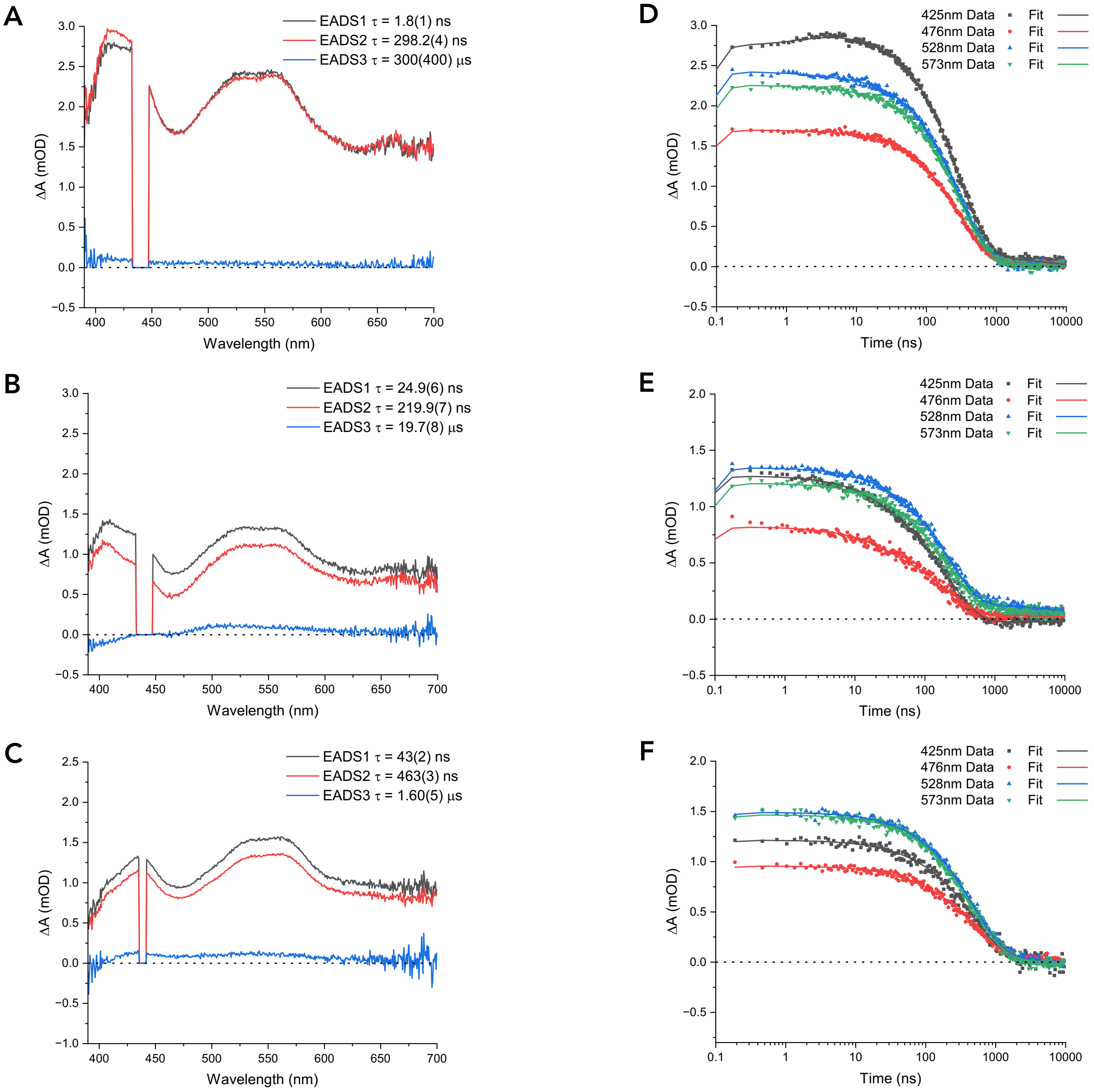

Figure S44. Global analysis of nanosecond visible transient absorption spectra (ns-TAS) of photocatalysts **Ir-BIP-Py 3-5** (OD_{400nm} = 0.2) with 0.1 M phthalimide ester **S** in THF, λ^{irr} = 440 nm. Panels **A**, **B**, and **C** display the evolution-associated difference spectra (EADS), and panels **D**, **E**, and **F** show kinetic traces and global analysis fits at selected wavelengths for photocatalysts **3**, **4**, and **5**, respectively.

The observed rate constant for electron transfer, k_{obs}, from the iridium photocatalysts to the phthalimide ester substrate (**S**) was determined from the excited state absorption lifetimes of compounds **2-5** in the absence and presence of 0.1 M of **S** in THF. The analysis considered k_{obs} instead of the electron transfer rate constant, k_{ET}, as the process was saturated with **S**. Excited-state absorption in the presence of 0.1 M **S** was fitted using Global Analysis (Glotaran).[12] The model assumed a monoexponential decay for all associated decay spectra. In the absence of **S**, the lifetime of the photocatalyst, τ_N, is the inverse of the sum of radiative and non-radiative decay rate constants and any other processes that depopulate the excited state (Equation 1). In the presence of 0.1 M **S**, the lifetime, τ_{N+S}, is determined by the inverse of the sum of radiative, non-radiative decay and electron transfer rate constants (Equation 2), and the pseudo-first-order rate constant for ET, k_{obs}, was calculated as the difference between the inverse of τ_{N+S} and the inverse of τ_N (Equation 3), with N = **1-5**.

$$\tau_N = \frac{1}{k_r + k_{nr}} \qquad \text{Equation 1.}$$

$$\tau_{N+S} = \frac{1}{k_r + k_{nr} + k_{obs}} \qquad \text{Equation 2.}$$

$$k_{obs} = \frac{1}{\tau_{N+S}} - \frac{1}{\tau_N} \qquad \text{Equation 3.}$$

Using compound **1** as a reference (τ_1 = 2.2 μs without **S**, 1.3 μs with 0.1 M **S**; $k_{obs} = 3.15 \times 10^5$ s^{-1}), compound **3** exhibited a k_{obs} value of 1.69×10^5 s^{-1} (τ_3 = 314.0 ns; τ_3+**S** = 298.2 ns). Similarly, compound **4**, (τ_4 = 223.6 ns; τ_4+**S** = 219.9 ns) yielded a smaller k_{obs} of 7.52×10^4 s^{-1}. **5** exhibited a decay lifetime of 527 ns and 463 ns, in the absence and presence of **S**, respectively, yielding a k_{obs} of 2.62×10^5 s^{-1} (see Table S1, Figures S49 and S50).

Table S1. Decay Lifetimes and k_{obs} values of Photocatalysts **1-5** with and without **S**.

**Photocatalyst**	τ_N	τ_{N+S}	k_{obs}
**1**	2.2 μs	1.3 μs	3.15 x 10^5 s^{-1}
**2**	290.0 ns	270 ns	2.55 x 10^5 s^{-1}
**3**	314.0 ns	298.2 ns	1.69 x 10^5 s^{-1}
**4**	223.6 ns	219.9 ns	7.52 x 10^4 s^{-1}
**5**	527 ns	463 ns	2.62 x 10^5 s^{-1}

2. Preparative Scale Photoredox Catalysis

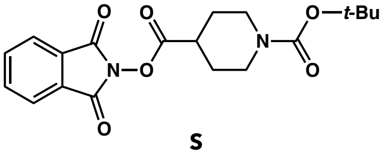

S

1-(*Tert*-Butyl)-4-(1,3-dioxoisoindolin-2-yl)piperidine-1,4-dicarboxylate (**S**). 1-(*tert*-butoxycarbonyl)piperidine-4-carboxylic acid (3.44 g, 15 mmol, 1.0 equiv.) was coupled with *N*-hydroxyphthalimide (2.69 g, 16.5 mmol, 1.1 equiv.) using *N*-(3-dimethylaminopropyl)-*N*′-ethylcarbodiimide hydrochloride (EDC, 3.01 g, 15.75 mmol, 1.05 equiv.) and 4-dimethylaminopyridine (DMAP, 0.183 g, 1.5 mmol, 0.1 equiv.) in anhydrous THF (75 mL) at room temperature overnight. The crude reaction mixture was extracted with water and DCM; the combined organic layers were dried over sodium sulfate and concentrated under reduced pressure. Finally, **S** was purified by flash silica gel column chromatography (0 – 30% EtOAc/hexane) as a white solid in 85% yield. ^{1}H NMR (500 MHz, $CDCl_3$): δ 7.88 (dd, J = 5.4, 3.1 Hz, 2H), 7.79 (dd, J = 5.5, 3.1 Hz, 2H), 4.03 (s, 2H), 3.01 (t, J = 11.1 Hz, 2H), 2.91 (ddd, J = 10.4, 6.4, 4.1 Hz, 1H), 2.06 (dd, J = 13.3, 3.0 Hz, 2H), 1.89 – 1.81 (m, 2H), 1.46 (s, 9H). Spectral data for compound F was consistent with literature reports[10].

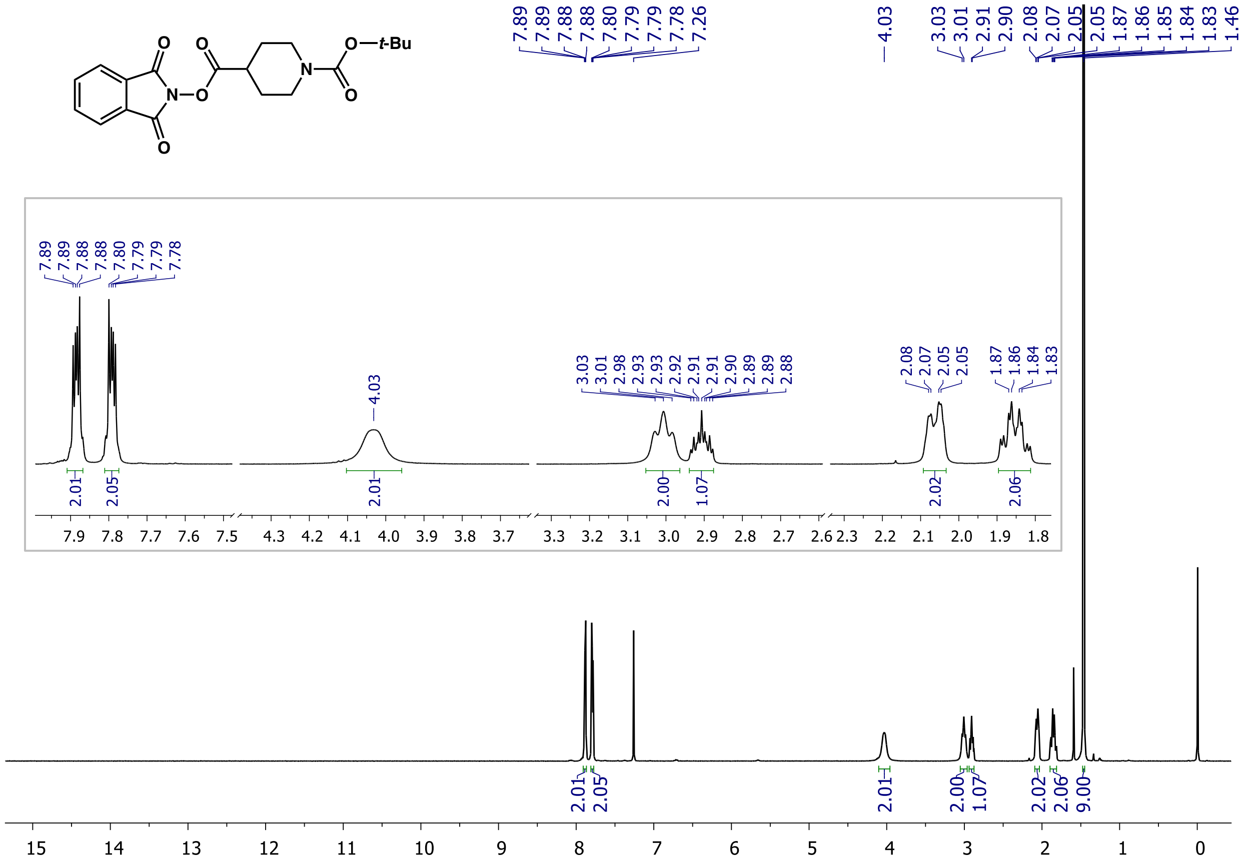

Figure S45. 500 MHz ^{1}H NMR spectrum of **S** in $CDCl_3$, (insets) expansion of selected regions.

2.1. Quantum Yield Determination

2.1.1. Measurement of Photon Flux using Ferrioxalate Actinometry

The photon flux of the Kessil lamp used in a typical photoredox reaction was determined using a standard ferrioxalate actinometer[11]. For the actinometry, the following materials are needed: potassium ferrioxalate trihydrate ($K_3Fe(C_2O_4)•3H_2O$) purchased from Alfa Aesar used without further purification; two sulfuric acid stock solutions with concentration of 0.05 and 0.5 M; 1,10-phenanthroline and sodium acetate (anhydrous). In a 100 mL volumetric flask, 0.27 mL of concentrated sulfuric acid (18.4 M) was added to 90 mL deionized water and then diluted to 100 mL mark to reach a final concentration of 0.05 M. A 0.15 M solution of potassium ferrioxalate was then prepared by dissolving potassium ferrioxalate trihydrate (1.84 g, 3.75 mmol) with the 0.05 M sulfuric acid solution prepared in a 25 mL volumetric flask. The potassium ferrioxalate was weighed as quickly as possible, along with taking every precaution to prepare and store the solution in the dark. A developer solution was prepared by dissolving 27.2 g of sodium acetate (anhydrous) into 200 mL of 0.5 M sulfuric acid. Then, 2.0 g of 1,10-phenanthroline was added to this solution, and the solution was also stored in the dark.

A typical experiment for measuring photon flux with this actinometer is as follows. A 1 cm by 1 cm quartz cuvette was charged with 3 mL of 0.15 M aqueous potassium ferrioxalate solution. Two sides of the cuvette were coated with black electrical tape to ensure a minimum pathway of the light of 1 cm. The ferrioxalate solution was stirred with a micro stir bar. While stirring, the solution was irradiated with the light source used in the preparative scale photoredox reaction at a distance of 4 cm from a Kessil lamp with λ_{max} = 440 nm equipped with a 440 nm bandpass interference filter with FWHM of 10 nm. The cuvette was irradiated for a set time period (0, 15, 30, 45 or 60 seconds), and then a 10 μL aliquot was removed from the irradiated sample. This aliquot was immediately added to 5 mL of the developer solution in a 2-dram (7.4 mL) vial wrapped in aluminum foil. Concurrently, a "blank" sample was prepared by diluting 10 μL of the stock solution (kept in the dark) into 5 mL of developer solution. The solutions were left in the dark for 30-45 min, becoming bright red. Then, 3 mL aliquots of the solutions were transferred to a cuvette and the absorbance spectrum of the $Fe(phen)_3^{2+}$ complex was obtained. The absorbance at 510 nm (ε = 11,100 $M^{-1}cm^{-1}$) was measured and used in the calculations.

2.1.2. Data Analysis for Photon Flux Measurement

Photon flux was calculated using the following equations. First, the number of moles of Fe^{2+} produced after an irradiation of *t* seconds are calculated:

$$\text{moles } Fe^{2+} = \frac{\Delta A_{510\text{ nm}}}{\varepsilon_{510\text{ nm}} \times l} \times \frac{V_1 V_3}{V_2} \qquad \text{Equation 4.}$$

where $\Delta_{A510\text{ nm}}$ is the difference in absorbance at 510 nm between the sample and the blank; $\varepsilon_{510\text{ nm}}$ is the extinction coefficient of $Fe(phen)_3$ complex at 510 nm (ε = 11,100 M^{-1} cm^{-1}); *l* is the path length of the cuvette (1 cm); and V_1, V_2, and V_3 are the dilution factors used when preparing the samples [V_1 = total volume of irradiated solution (3 mL), V_2 = volume of aliquot taken from V_1 (10 µL), V_3 = volume that V_2 is diluted into (5 mL)].

The photon flux is then determined using the calculated number of moles of Fe^{2+} produced at each timepoint:

$$\text{photon flux} = \frac{\text{moles } Fe^{2+}}{\phi_\lambda \times F \times t} \qquad \text{Equation 5.}$$

where φ_λ is the quantum yield of the ferrioxalate decomposition at λ (436 nm), *F* is the mean fraction of light absorbed by the ferrioxalate solution at λ, and *t* is the irradiation time in seconds. At 436 nm, the quantum yield of the actinometer is reported as 1.01 and the fraction of light absorbed is 0.997. These values were used for the calculations with the 440 nm Kessil lamp.

Table S2. Photon flux determination for 440 nm Kessil lamp with potassium ferrioxalate actinometer.

Time (sec)	ΔA, Trial 1	ΔA, Trial 2
0	0	0
15	-	0.1030
30	-	0.1667
45	0.2600	0.2494
60	0.3074	0.3016
**ΔA/s**	5.36×10^{-3}	5.31×10^{-3}
**mol Fe^{2+}/s**	7.26×10^{-7}	7.24×10^{-7}
**Photon flux (einstein/s)**	7.17×10^{-7}	7.15×10^{-7}
**Average photon flux (einstein/s)**	$(7.16 \pm 0.01) \times 10^{-7}$	

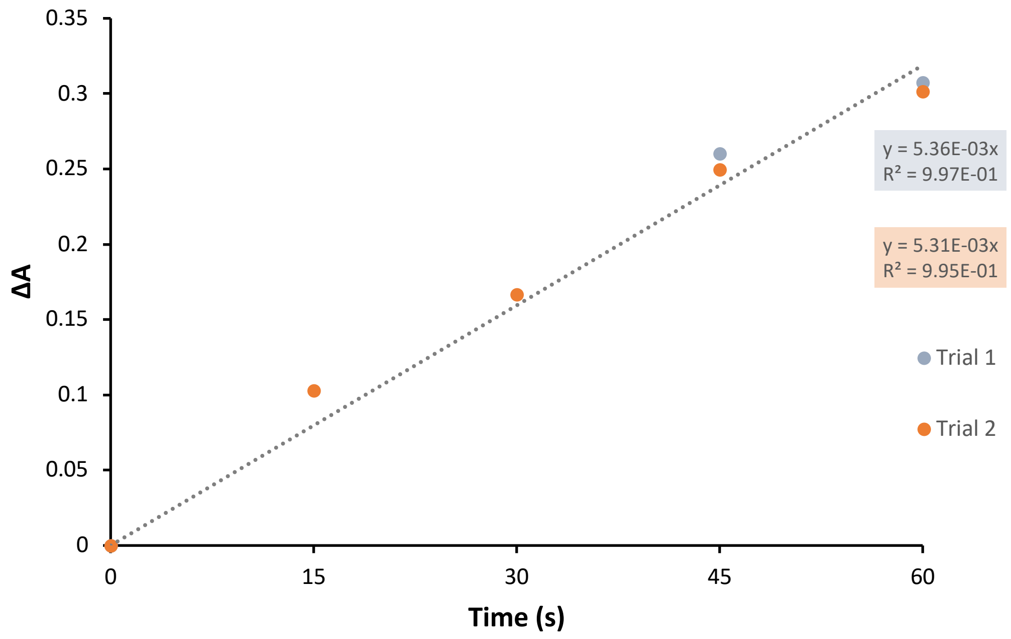

Figure S46. Changes in absorbance at 440 nm versus time with the potassium ferrioxalate actinometer.

2.1.3. Initial Rates Kinetics for Quantum Yield Determination at 440 nm

A 1 cm × 1 cm quartz cuvette with micro stir bar was charged with **S** (112 mg, 0.3 mmol), photocatalyst ($OD_{440\ nm}$ = 1.1, see absorption spectra below for photocatalyst specific loading concentration values), diphenyl ether (23.7 µL, 0.15 mmol – internal standard for GC analysis), and tetrahydrofuran (3.0 mL, 0.1 M). The cuvette was sealed in a glovebox with a screw-on cap and septum, and then a nitrogen inlet needle was inserted through the septum. Two sides of the cuvette were blacked out with electrical tape to ensure a path length of at least 1 cm. After injecting thiophenol (30.6 µL, 0.30 mmol) through the septum, the cuvette was placed inside a water bath a distance of 4 cm from the lamp, and with an overhead fan for cooling to 25 °C. The reaction was irradiated with a 40 W Kessil lamp with maximum wavelength of 440 nm, fitted with a 440 nm bandpass interference filter with a FWHM of 10 nm. Reaction aliquots of 10 µL were removed at various timepoints and yields were determined by GC.

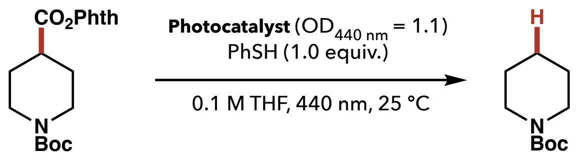

S **P**

Scheme S2. Photocatalytic phthalimide ester reduction.

Table S3. Initial rates of N-hydroxyphthalimide ester **S** reduction with **1** at 440 nm.

**Time (s)**	**[*N*-Boc-piperidine] M**	
	**Trial 1**	**Trial 2**
0	0	0
3600	0.0009	0.0011
7200	0.0018	0.0020
10800	0.0027	0.0029
14400	0.0037	0.0039
18000	0.0048	-
**Rate (M/s)**	2.60×10^{-7}	2.73×10^{-7}
**Average rate (M/s)**	$(2.67 \pm 0.07) \times 10^{-7}$	

Table S4. Initial rates of N-hydroxyphthalimide ester **S** reduction with **2** at 440 nm.

**Time (s)**	**[*N*-Boc-piperidine] M**	
	**Trial 1**	**Trial 2**
0	0	0
3600	0.0011	0.0011
7200	0.0025	-
10800	0.0038	0.0035
14400	0.0048	0.0049
18000	-	0.0060
**Rate (M/s)**	3.41×10^{-7}	3.33×10^{-7}
**Average rate (M/s)**	$(3.37 \pm 0.04) \times 10^{-7}$	

Table S5. Initial rates of *N*-hydroxyphthalimide ester **S** reduction with **3** at 440 nm.

**Time (s)**	**[*N*-Boc-piperidine] M**	
	**Trial 1**	**Trial 2**
0	0	0
3600	0.0021	0.0023
7200	0.0043	0.0042
10800	0.0062	0.0062
14400	0.0082	0.0083
18000	0.0107	-
**Rate (M/s)**	5.83×10^{-7}	5.77×10^{-7}
**Average rate (M/s)**	$(5.80 \pm 0.03) \times 10^{-7}$	

Table S6. Initial rates of *N*-hydroxyphthalimide ester **S** reduction with **4** at 440 nm.

**Time (s)**	**[*N*-Boc-piperidine] M**	
	**Trial 1**	**Trial 2**
0	0	0
3600	0.0022	-
7200	0.0052	0.0051
10800	0.0078	0.0076
14400	0.0100	0.0097
18000	0.0120	0.0121

**Rate (M/s)**	6.88 x 10^{-7}	6.81 x 10^{-7}
**Average rate (M/s)**	(6.85 ± 0.04) x 10^{-7}	

Table S7. Initial rates of N-hydroxyphthalimide ester **S** reduction with **5** at 440 nm.

**Time (s)**	**[*N*-Boc-piperidine] M**	
	**Trial 1**	**Trial 2**
0	0	0
3600	0.0006	0.0007
7200	0.0007	0.0009
10800	0.0009	0.0011
14400	0.0011	0.0015
18000	0.0014	0.0016
**Rate (M/s)**	9.89 x 10^{-8}	8.26 x 10^{-8}
**Average rate (M/s)**	(9.08 ± 0.08) x 10^{-8}	

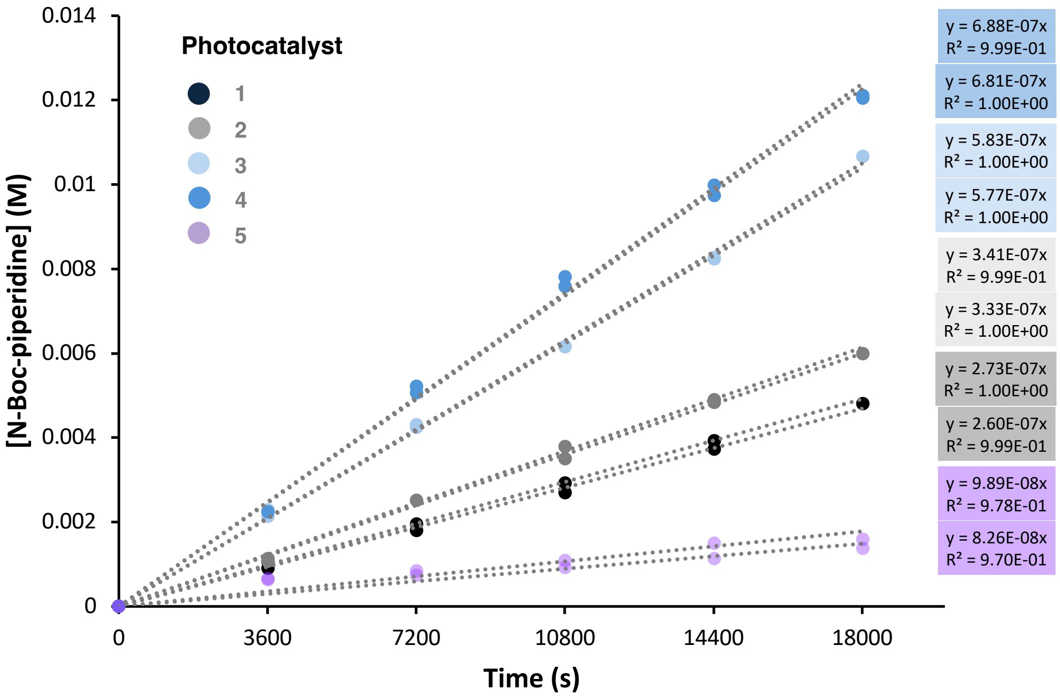

Figure S47. Concentration of *N*-hydroxyphthalimide ester **S** formed versus time with photocatalysts **1-5**.

2.1.4. UV/VIS Absorption spectra of Photocatalysts at Preparative Scale Concentrations

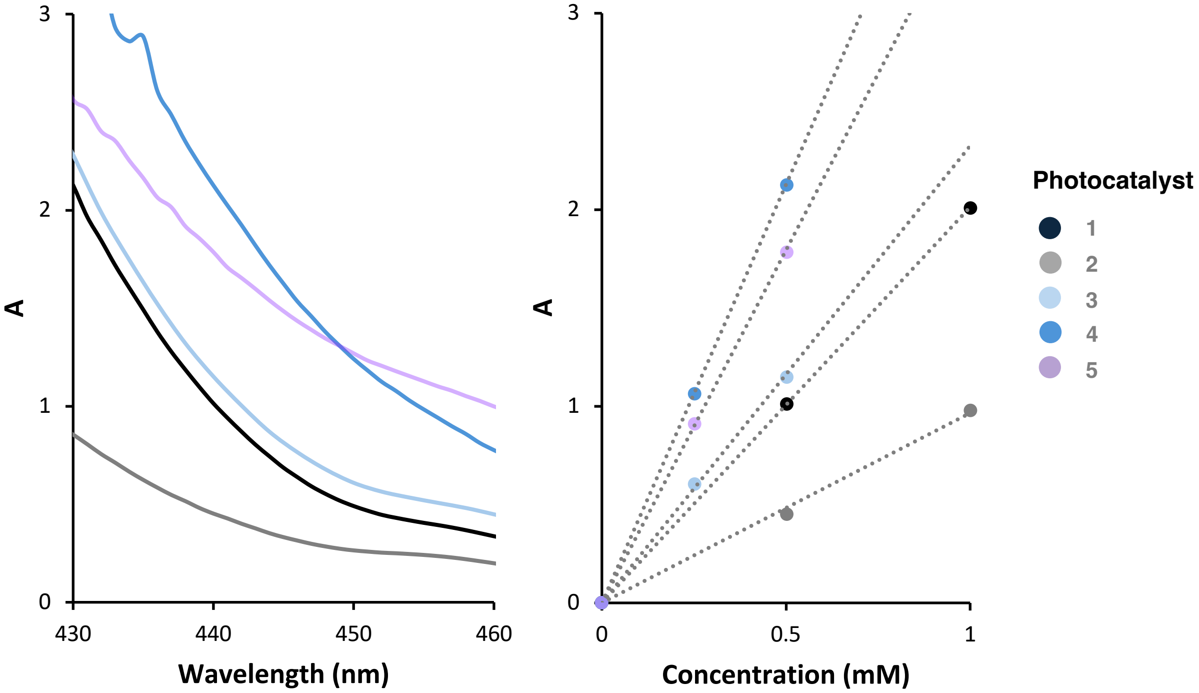

Figure S48. *Left:* Absorption spectra of **1-5**, 0.5 mM in THF. *Right:* Absorbance at 440 nm versus concentration of **1-5**. Preparative scale reactions were absorbance matched at an O.D of 1.1, which translates to [**1**] = 1.15 mM, [**2**] = 0.55 mM, [**3**] = 0.47 mM, [**4**] = 0.26 mM, and [**5**] = 0.30 mM.

2.1.5. Calculation of Internal Quantum Yield

After the initial rate of the reaction of interest has been determined, the quantum yield of the reaction is calculated in the following manner:

$$\text{Quantum Yield } (\Phi) = \frac{\text{Average rate } (\text{mol L}^{-1}s^{-1}) \times \text{Reaction volume (L)}}{\text{Photon flux } s^{-1} \times F} \qquad \text{Equation 6.}$$

Multiplying the rate (in M/s) by the reaction volume (in L) gives the moles of product produced in the reaction each second. Dividing this number by the photon flux gives the overall external quantum yield of the reaction. Because the photocatalysts are absorbance-matched in the initial rates experiments using the 440 nm lamp ($OD_{440\ nm}$ = 1.1), the quantum yield calculation is adjusted with the fraction of photons absorbed by the photocatalysts, where *F* is defined as the fraction of light absorbed by the photocatalyst at 440 nm. With an OD = 1.1, the percent transmittance is 8%, so *F* = 0.92.

Table S8. Average internal quantum yields for **1-5** at 440 nm.

Photocatalyst	Average Internal Quantum Yield (10^{-3})
**1**	1.2
**2**	1.5
**3**	2.6
**4**	3.1
**5**	0.4

2.2. Catalytic Cycle

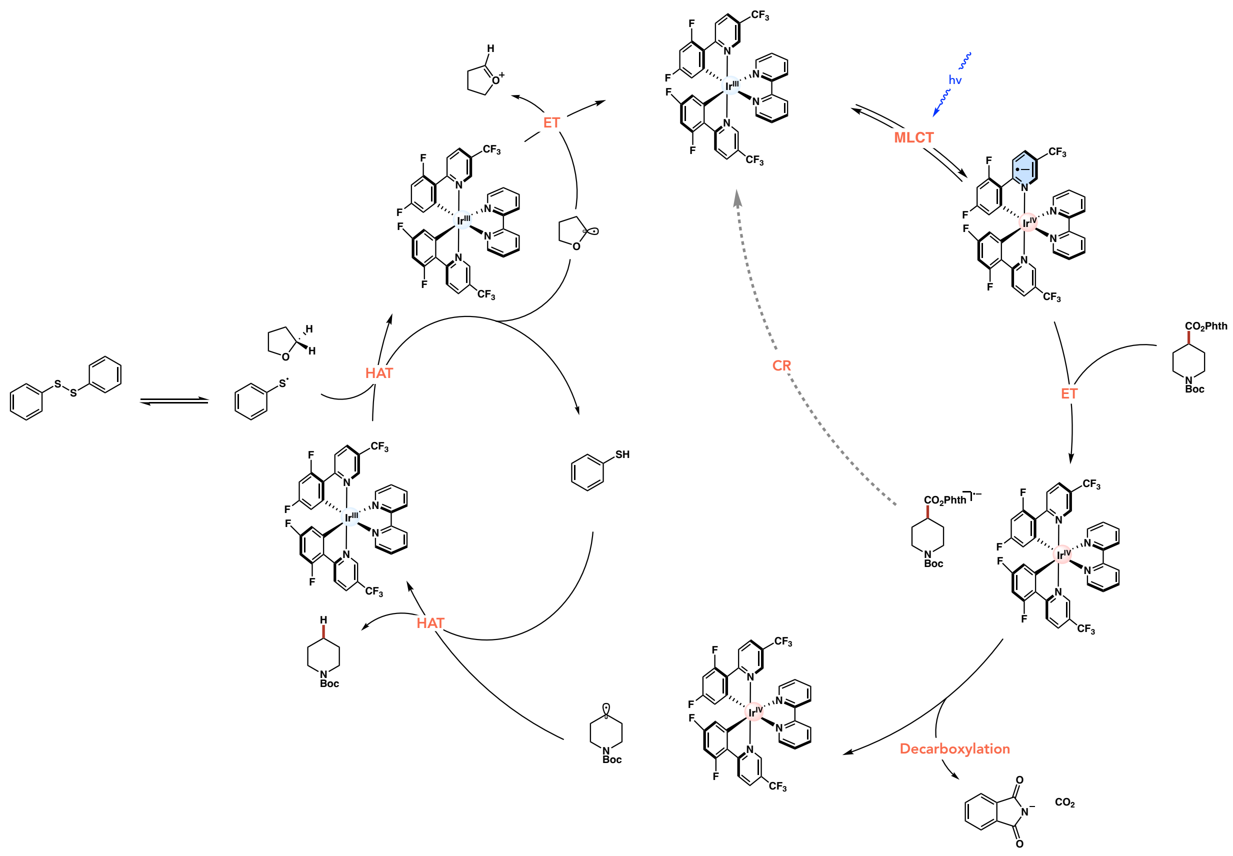

Scheme S3. Proposed catalytic cycle of *N*-hydroxyphthalimide ester reduction with photocatalyst **1.**

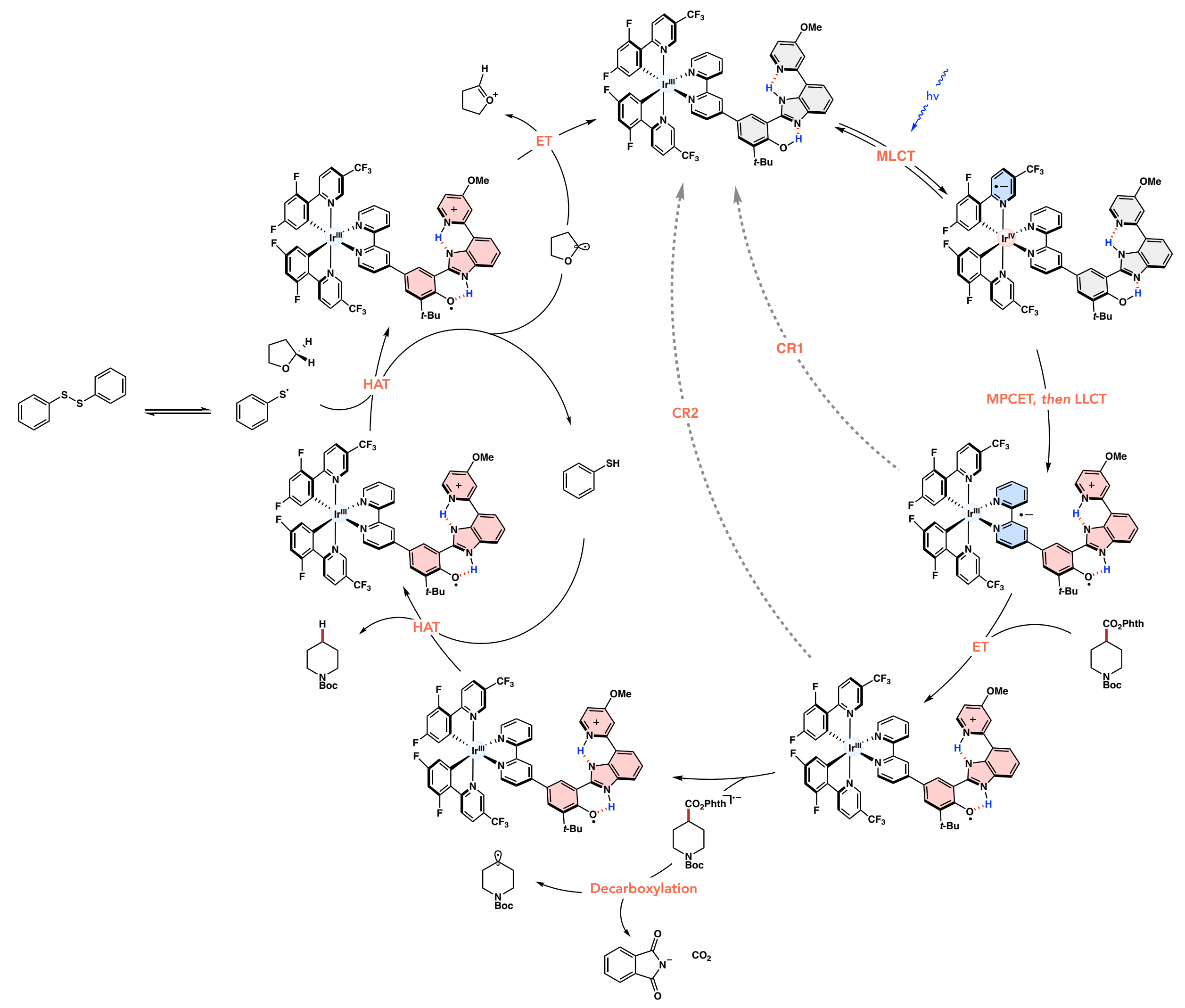

Scheme S4. Proposed catalytic cycle of *N*-hydroxyphthalimide ester reduction with photocatalyst **3-5.**

2.3. Determination of Charge Recombination Ratios

The ratio for charge recombination with **1-5** was determined utilizing the experimentally determined quantum yields and excited state lifetimes in the presence and absence of 10 mM phthalimide ester (**S**).

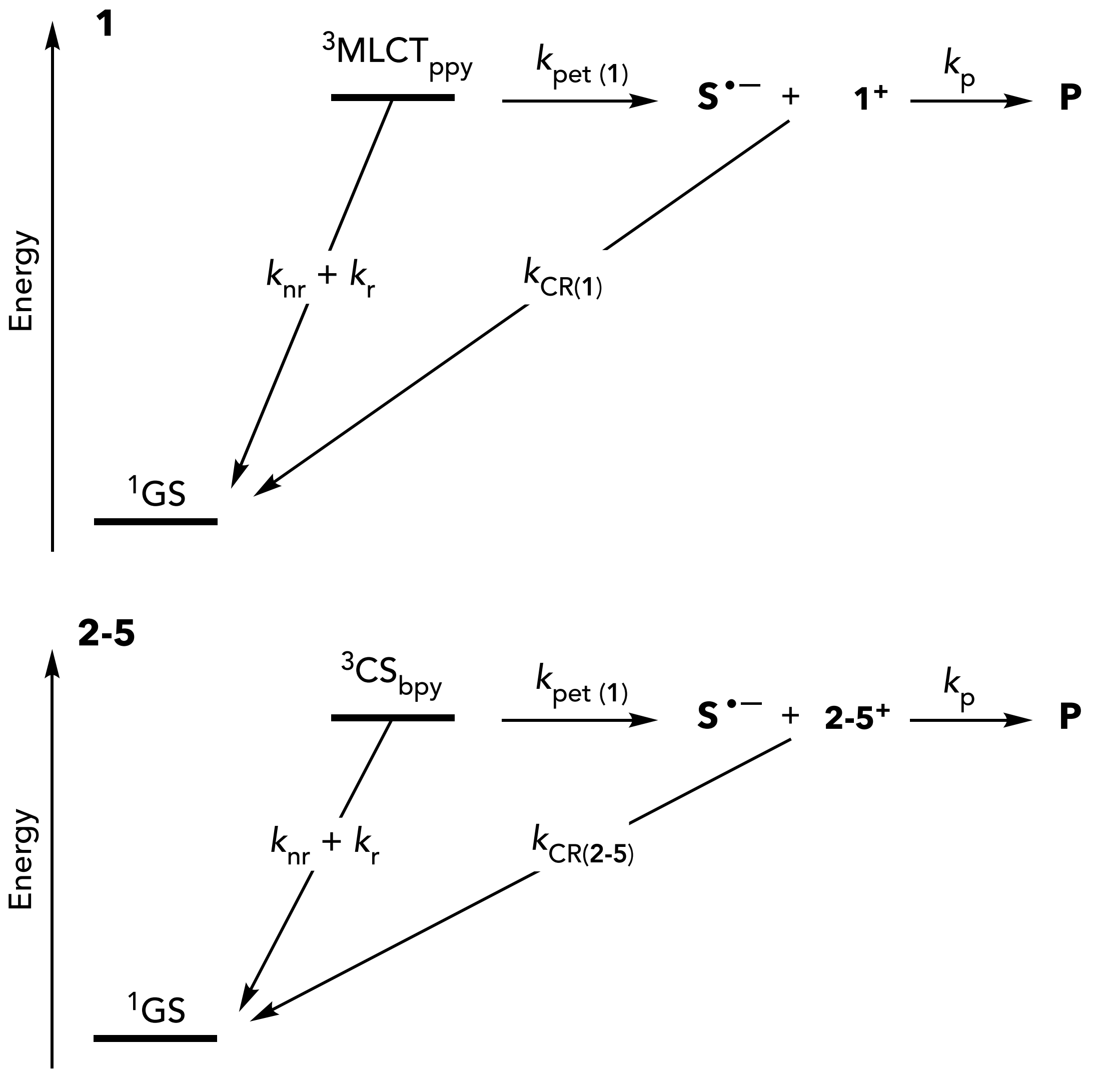

Figure S49. Kinetic scheme for the photochemical reaction with either **1*** and **S** or **2-5*** and **S** to produce product **P**.

$$\frac{\Phi_{\mathbf{1}}}{\Phi_{\mathbf{2-5}}} = \frac{\Phi_{3_{MLCT\,ppy}}}{\Phi_{3_{CS\,bpy}}} \times \frac{Y_{\mathbf{1}}}{Y_{\mathbf{2-5}}} \times \frac{V_{\mathbf{1}}}{V_{\mathbf{2-5}}}$$

$$\frac{\Phi_{3_{MLCT\,ppy}}}{\Phi_{3_{CS\,bpy}}} \cong 1$$

$$Y_{\mathbf{1}} = \frac{k_{pet(\mathbf{1})}}{k_{pet(\mathbf{1})} + k_{nr} + k_r} = \frac{\tau_{\mathbf{1}} - \tau_{\mathbf{1}+S}}{\tau_{\mathbf{1}+S}} = \frac{\Delta\tau_{\mathbf{1}}}{\tau_{\mathbf{1}+S}}$$

$$Y_{\mathbf{2-5}} = \frac{k_{pet(\mathbf{2-5})}}{k_{pet(\mathbf{2-5})} + k_{nr} + k_r} = \frac{\tau_{\mathbf{2-5}} - \tau_{\mathbf{2-5}+S}}{\tau_{\mathbf{2-5}+S}} = \frac{\Delta\tau_{\mathbf{2-5}}}{\tau_{\mathbf{2-5}+S}}$$

$$V_{\mathbf{1}} = \frac{k_P[\boldsymbol{S}^{\cdot-}]}{k_P[\boldsymbol{S}^{\cdot-}] + k_{CR(\mathbf{1})}[\mathbf{1}^+][\boldsymbol{S}^{\cdot-}]} = \frac{k_P}{k_P + k_{CR(\mathbf{1})}[\mathbf{1}^+]}$$

$$V_{\mathbf{2-5}} = \frac{k_P[S^{\cdot-}]}{k_P[\boldsymbol{S}^{\cdot-}] + k_{CR(\mathbf{2-5})}[\mathbf{2-5}^+][\boldsymbol{S}^{\cdot-}]} = \frac{k_P}{k_P + k_{CR(\mathbf{2-5})}[\mathbf{2-5}^+]}$$

$$\frac{\Phi_{\mathbf{1}}}{\Phi_{\mathbf{2-5}}} \cong \frac{\Delta\tau_{\mathbf{1}}/\tau_{\mathbf{1}+S}}{\Delta\tau_{\mathbf{2-5}}/\tau_{\mathbf{2-5}+S}} \times \frac{\frac{k_P}{k_P + k_{CR(\mathbf{1})}[\mathbf{1}^+]}}{\frac{k_P}{k_P + k_{CR(\mathbf{2-5})}[\mathbf{2-5}^+]}}$$

Assuming $k_{CR} >> k_P$:

$$\frac{\Phi_{\mathbf{1}}}{\Phi_{\mathbf{2-5}}} \cong \frac{\Delta\tau_{\mathbf{1}}/\tau_{\mathbf{1}+S}}{\Delta\tau_{\mathbf{2-5}}/\tau_{\mathbf{2-5}+S}} \times \frac{k_{CR(\mathbf{2-5})}[\mathbf{2-5}^+]}{k_{CR(\mathbf{1})}[\mathbf{1}^+]}$$

$$\frac{k_{CR(\mathbf{2-5})}[\mathbf{2-5}^+]}{k_{CR(\mathbf{1})}[\mathbf{1}^+]} \cong \frac{\Phi_{\mathbf{1}}}{\Phi_{\mathbf{2-5}}} \times \frac{\Delta\tau_{\mathbf{2-5}}/\tau_{\mathbf{2-5}+S}}{\Delta\tau_{\mathbf{1}}/\tau_{\mathbf{1}+S}}$$

$\frac{k_{CR(\mathbf{2})}[\mathbf{2}^+]}{k_{CR(\mathbf{1})}[\mathbf{1}^+]} \cong 8.4 \times 10^{-2}$	$\frac{k_{CR(\mathbf{1})}[\mathbf{1}^+]}{k_{CR(\mathbf{2})}[\mathbf{2}^+]} \cong 12$
$\frac{k_{CR(\mathbf{3})}[\mathbf{3}^+]}{k_{CR(\mathbf{1})}[\mathbf{1}^+]} \cong 3.5 \times 10^{-2}$	$\frac{k_{CR(\mathbf{1})}[\mathbf{1}^+]}{k_{CR(\mathbf{3})}[\mathbf{3}^+]} \cong 28$
$\frac{k_{CR(\mathbf{4})}[\mathbf{4}^+]}{k_{CR(\mathbf{1})}[\mathbf{1}^+]} \cong 9.4 \times 10^{-3}$	$\frac{k_{CR(\mathbf{1})}[\mathbf{1}^+]}{k_{CR(\mathbf{4})}[\mathbf{4}^+]} \cong 106$
$\frac{k_{CR(\mathbf{5})}[\mathbf{5}^+]}{k_{CR(\mathbf{1})}[\mathbf{1}^+]} \cong 0.6$	$\frac{k_{CR(\mathbf{1})}[\mathbf{1}^+]}{k_{CR(\mathbf{5})}[\mathbf{5}^+]} \cong 1.7$

2.4. Static vs. Dynamic Quenching of the Excited State of 4

To investigate the possibility of static quenching, where complex formation between compound **4** and quencher **S** occurs, ^{1}H NMR spectra were acquired for photocatalyst **4**, substrate **S**, and a mixture of both in $CDCl_3$. The absence of chemical shift changes in the NMR spectra of either compound upon mixing indicates that no appreciable complexation occurs between **4** and **S** in solution. Therefore, the observed quenching of compound **4**'s excited state by compound **S** is attributed to a dynamic quenching mechanism.

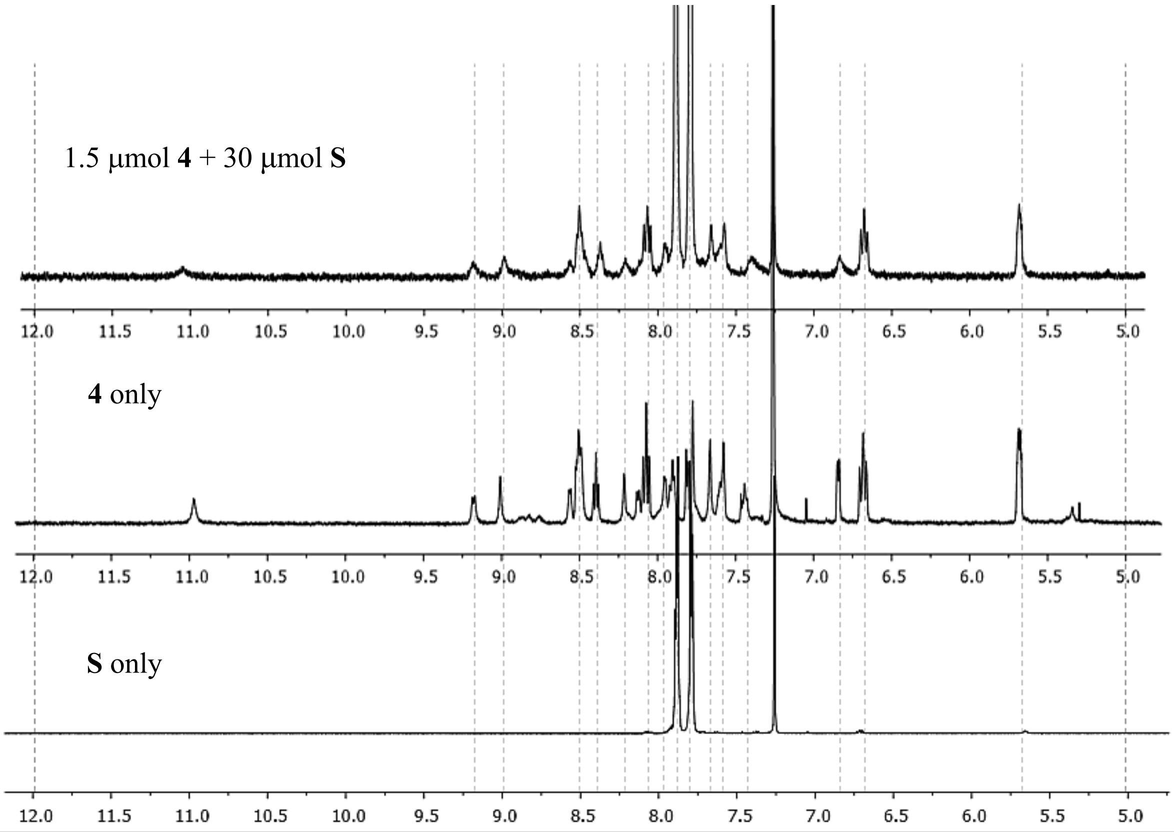

Figure S50. ^{1}H NMR (500 MHz, $CDCl_3$) of **S** (bottom), **4** (middle), and 1.5 µmol **4** + 30 µmol **S** (top).

3. References

1. Singh, A. *et al.* Facile Synthesis and Complete Characterization of Homoleptic and Heteroleptic Cyclometalated Iridium(III) Complexes for Photocatalysis. *J Organomet Chem* 2015, **776**, 51–59.
2. Sayre, H. *et al.* PCET-Based Ligand Limits Charge Recombination with an Ir(III) Photoredox Catalyst. *J. Am. Chem. Soc.* 2021, **143**, 13034–13043.
3. Guerra, W. D. *et al.* Role of Intact Hydrogen-Bond Networks in Multiproton-Coupled Electron Transfer. *J. Am. Chem. Soc.* 2020 **142**, 21842–21851.
4. Odella, E. *et al.* Managing the Redox Potential of PCET in Grotthuss-Type Proton Wires. *J. Am. Chem. Soc.* 2022, **144**, 15672–15679.
5. Huynh, M. T. *et al.* Concerted One-Electron Two-Proton Transfer Processes in Models Inspired by the Tyr-His Couple of Photosystem II. *ACS Cent. Sci.* 2017, **3**, 372–380.
6. Odella, E. *et al.* Proton-coupled electron transfer across benzimidazole bridges in bioinspired proton wires. *Chem. Sci.* 2020, **11**, 3820–3828.
7. Connelly, N. G. & Geiger, W. E. *Chemical Redox Agents for Organometallic Chemistry.* https://pubs.acs.org/sharingguidelines (1996).
8. Odella, E. *et al.* Controlling Proton-Coupled Electron Transfer in Bioinspired Artificial Photosynthetic Relays. *J. Am. Chem. Soc.* 2018, **140**, 15450–15460.
9. Snellenburg, J. J., Laptenok, S. P., Seger, R., Mullen, K. M. & Van Stokkum, I. H. M. Glotaran: A Java-Based Graphical User Interface for the R Package TIMP. *JSS Journal of Statistical Software* 2012, **49**, 1–22.
10. Yu, L. *et al.* Zinc-Mediated Decarboxylative Alkylation of Gem-difluoroalkenes. *Org. Lett.* 2018 **20**, 4579–4583.
11. Hatchard, C. G. & Parker, C. A. A new sensitive chemical actinometer II. Potassium ferrioxalate as a standard chemical actinometer. *Phys. Sci.* 1956, **235**, 518–536.
12. Snellenburg, J. J.; Laptenok, S.; Seger, R.; Mullen, K. M.; Stokkum, I. H. M. van. Glotaran: A Java-Based Graphical User Interface for the R Package TIMP. *J. Stat. Softw.* 2012, **49**, 1–22.
